# Targeting the tumor mutanome for personalized vaccination in a TMB low non-small cell lung cancer

**DOI:** 10.1136/jitc-2021-003821

**Published:** 2022-03-30

**Authors:** Katy McCann, Adrian von Witzleben, Jaya Thomas, Chuan Wang, Oliver Wood, Divya Singh, Konstantinos Boukas, Kaidre Bendjama, Nathalie Silvestre, Finn Cilius Nielsen, Gareth Thomas, Tilman Sanchez-Elsner, Jason Greenbaum, Stephen Schoenberger, Bjoern Peters, Pandurangan Vijayanand, Natalia Savelyeva, Christian Ottensmeier

**Affiliations:** 1 Cancer Research UK Southampton Experimental Cancer Medicine Centre, Cancer Sciences Unit, University of Southampton Faculty of Medicine, Southampton, UK; 2 Department of Otorhinolaryngology, Head & Neck Surgery, University of Ulm, Ulm, Germany; 3 Head and Neck Centre, Institute of Systems, Molecular and Integrative Biology, University of Liverpool, Liverpool, UK; 4 Research and Development Department, Transgene, Illkirch-Graffenstaden, France; 5 Center for Genomic Medicine, Rigshospitalet, Kobenhavn, Denmark; 6 University Hospital Southampton NHS Foundation Trust, Southampton, UK; 7 Clinical and Experimental Sciences, University of Southampton Faculty of Medicine, Southampton, UK; 8 Center for Infectious Disease and Vaccine Research, La Jolla Institute for Immunology, La Jolla, California, USA; 9 Laboratory of Cellular Immunology, La Jolla Institute for Immunology, La Jolla, California, USA; 10 La Jolla Institute for Immunology, La Jolla, California, USA

**Keywords:** Immunogenicity, Vaccine, Lung Neoplasms, Lymphocytes, Tumor-Infiltrating, T-Lymphocytes

## Abstract

**Background:**

Cancer is characterized by an accumulation of somatic mutations, of which a significant subset can generate cancer-specific neoepitopes that are recognized by autologous T cells. Such neoepitopes are emerging as important targets for cancer immunotherapy, including personalized cancer vaccination strategies.

**Methods:**

We used whole-exome and RNA sequencing analysis to identify potential neoantigens for a patient with non-small cell lung cancer. Thereafter, we assessed the autologous T-cell reactivity to the candidate neoantigens using a long peptide approach in a cultured interferon gamma ELISpot and tracked the neoantigen-specific T-cells in the tumor by T-cell receptor (TCR) sequencing. In parallel, identified gene variants were incorporated into a Modified Vaccinia Ankara-based vaccine, which was evaluated in the human leucocyte antigen A*0201 transgenic mouse model (HHD).

**Results:**

Sequencing revealed a tumor with a low mutational burden: 2219 sequence variants were identified from the primary tumor, of which 23 were expressed in the transcriptome, involving 18 gene products. We could demonstrate spontaneous T-cell responses to 5/18 (28%) mutated gene variants, and further analysis of the TCR repertoire of neoantigen-specific CD4^+^ and CD8^+^ T cells revealed TCR clonotypes that were expanded in both blood and tumor tissue. Following vaccination of HHD mice, de novo T-cell responses were generated to 4/18 (22%) mutated gene variants; T cells reactive against two variants were also evident in the autologous setting. Subsequently, we determined the major histocompatibility complex restriction of the T-cell responses and used in silico prediction tools to determine the likely neoepitopes.

**Conclusions:**

Our study demonstrates the feasibility of efficiently identifying tumor-specific neoantigens that can be targeted by vaccination in tumors with a low mutational burden, promising successful clinical exploitation, with trials currently underway.

## Introduction

The accumulation of genetic alterations represents a key driver of cancer. Landmark studies have demonstrated that human cancers harbor 10s to many 100s of somatic mutations and that the burden of such mutations varies according to cancer type, with lung cancer exhibiting one of the highest mutational loads –up to 400 mutations per Mb.^1^ The majority of mutations observed are unique to an individual patient’s tumor and can result in alterations to translated gene products (non-synonymous mutations), which may create novel epitopes, termed neoepitopes, that can be presented on major histocompatibility complex (MHC) molecules for recognition by T cells.^2^ Neoepitopes represent attractive immunotherapy targets as they are not subject to central tolerance that would limit both the frequency and function of T cells specific for self-antigens. Indeed, a proportion of neoepitopes have been shown to be immunogenic, eliciting spontaneous T-cell responses that are both MHC class I and II restricted, as has been previously described in patients with melanoma and lung cancer.^3–7^ However, the number of CD8^+^ and CD4^+^ neoantigen-specific T cells detectable within tumor-infiltrating lymphocytes (TIL) is low, even though the number of potential neoepitopes predicted by in silico algorithms is relatively high; reports suggest that 1%–2% of tumor mutations result in neoantigens that are presented and spontaneously recognized by T cells.^8^ Circulating neoantigen-specific T cells can also be found in the peripheral blood of patients.^9^ Multiple studies emphasize a critical role for neoantigen-specific T cells, with clear clinical benefit associated with the presence of neoantigen-specific T cells in the blood and tumor within the context of immune checkpoint blockade, for example, anti-PD-1, anti-PD-L1 and anti-CTLA4 antibodies.^10–13^ In general, cancers with a high tumor mutational burden (TMB) and high numbers of predicted neoantigens exhibit better objective responses to checkpoint inhibitors.^11 12^ However, neoantigens can also be potentially identified in low TMB cancers, where mutational frameshifts, splice variants or gene fusions can provide a source for potent immunogenic neoantigens.

Next-generation sequencing (NGS) technology has enabled high-throughput DNA and RNA sequencing and provides a tool to comprehensively map the somatic mutations in an individual’s tumor (‘tumor mutanome’) quickly and at a reasonable cost. Several resources, including the Immune Epitope Database and Analysis Resource (IEDB; www.iedb.org), are freely available to predict and analyze T cell epitopes,^14^ although there is no standard universal workflow for neoantigen prediction. Collectively, it is possible to identify neoantigen candidates as potential biomarkers or therapeutic targets for individual patients, paving the way for therapeutic vaccination approaches. Preclinical studies using murine tumor models have shown the efficacy and feasibility of neoantigen-targeted cancer vaccines and have expedited the development of human trials.^15^ To date, a number of phase I/II clinical trials of neoantigen vaccines utilizing viral vectors, DNA, RNA, synthetic long peptides and dendritic cells are underway in various cancer types, many in combination with immune checkpoint blockade to further enhance the anti-tumor effect.^16 17^ Two significant studies in 2017 confirmed the potent role of neoantigen-based vaccination in melanoma, demonstrating that neoantigen vaccination in combination with checkpoint blockade can significantly enhance vaccine-induced immune responses and lead to lasting clinical effect in some patients; Ott *et al* and Sahin *et al* demonstrated that 4/6 and 8/13 patients receiving neoantigen vaccine remained recurrence-free during follow-up, respectively.^6 13^ Furthermore, a phase 1 trial using a personalized neoantigen peptide vaccine in glioblastoma, typically an immune ‘cold’ tumor, reported that vaccination induced predominately CD4^+^ T-cell responses of T helper 1 type against predicted neoepitopes, with favorable median overall survival (OS) and progression-free survival (PFS).^18^

The tracking non-small cell lung cancer (NSCLC) evolution through therapy study, or TRACERx, demonstrates that a high clonal neoantigen load exhibits significantly improved disease-free survival,^19^ while there is also evidence suggesting autologous neoantigen-specific TILs play an important role in tumor control.^2 20–22^ In this study, we examine the feasibility of personalized neoantigen vaccination for patients with TMB low lung cancer, from identification of the tumor mutanome by NGS, selection and validation of candidate neoantigens and generation and preclinical testing of a personalized neoantigen vaccine, MyVac.^23^ Furthermore, we assess immune visibility of the tumor by T-cell receptor sequencing (TCR-seq), evaluating the presence of neoantigen-specific T cells in the blood and tumor.

## Materials and methods

### Patient details

A male in his mid-60s and never-smoker was diagnosed in 2016 with non‐small‐cell adenocarcinoma of the lung: Stage IIIB; EGFR mutation negative; ALK translocation negative; PD-L1 positive (>1%). Following diagnosis and prior to receiving treatment, the patient underwent surgical resection of the primary lung tumor in June 2016 (TP01). Treatment commenced in July 2016 with two courses of Cisplatin/Pemetrexed chemotherapy (standard dosing) without benefit, followed by two doses of nivolumab (3 mg/kg) in August 2016; an apheresis product was collected post-first dose of Nivolumab. The patient experienced disease progression, evident by CT, and a second biopsy was collected from a parasternal metastasis in Sept 2016 (TP02). The patient received a further dose of nivolumab and one cycle of docetaxel chemotherapy (75 mg/m^2^) in September 2016, followed by radiotherapy to the sternal/parasternal mass (8 Gy in 1 fraction) in October 2016. Death from progressive disease was in January 2017.

### Immunohistochemistry

Immunohistochemistry (IHC) was performed on formalin-fixed, paraffin-embedded primary tumor tissue using the antibodies: CD3 (clone IR503; DAKO), CD8a (clone: C8/144B; DAKO), CD4 (clone 4B12; DAKO), CD103 (clone EPR4166(2); Abcam), PD-1 (clone NAT105; Abcam), PD-L1 (clone E1L3N; CST), MHCI ABC (clone EMR8-5; Abcam) and MHCII DR+DP+ DQ (clone: CR3/43; Abcam). Stained slides were scanned using a Zeiss Axio Scan.Z1 Digital Slide Scanner and Zeiss ZEN software (V.2.6) and positive cells were enumerated using the QuPath bioimage analysis software (V.0.2.9-m9); automated positive cell counting was on three independent regions, with the diaminobezidine mean as the score compartment.^24^

### NGS

DNA and RNA were extracted from fresh tumor tissue stored in RNAlater stabilization reagent (ThermoFisher Scientific) using AllPrep DNA/RNA Extraction Kit (Qiagen), according to the manufacturer’s instructions. DNA was isolated from whole blood using the liquid handling automated station (Tecan). Whole exome sequencing (WES) of tumor and germline DNA used SureSelect Clinical Research Exome with fragmentation to 300 bp, Covaris S2 (Agilent) and KAPA HTP Library Preparation Kit (Roche). RNA sequencing (RNA-seq) used TruSeq Stranded Total RNA Library Prep Kit (Illumina). WES and RNA-seq libraries were paired-end sequenced on Illumina NextSeq500 or HiSeq2500 platforms to yield an average coverage of 50–100X. Variant calling was as described in the online supplemental material. Sequencing data have been deposited in the Gene Expression Omnibus (GEO) under accession number GSE179879. The immune profile of the primary tumor and metastasis was contextualized using RNA-seq data from (1) a cohort of 47 consecutively collected head and neck squamous cell carcinomas (HNSCC)–data available in GEO under accession number GSE72536,^25^ and (2) a cohort of 18 lung tumor samples, data available in GEO under accession number GSE179879.

10.1136/jitc-2021-003821.supp1 Supplementary data

### Peptides for immunogenicity testing

Non-synonymous mutations identified by NGS were studied in the context of respective 20 amino acid peptide pairs, with the mutant (MUT) or wild type (WT) amino acid at position 6 or position 15; however, this was ultimately determined by the overall position of the MUT amino acid in the gene transcript and, as in some cases, the existence of multiple gene isotypes, and was therefore subject to variation. In the case of a DEL/frameshift MUT gene, multiple overlapping 20mer peptides were generated for the non-templated amino acids up to the length of the native sequence. Peptides were synthesized to >95% purity by Peptide Protein Research, Fareham, UK. All peptides generated for a particular MUT gene were assessed either as individual peptides or as a pool of 2 (n=12), 3 (n=3), 4 (n=1) or 8 (n=1) peptides (online supplemental table S1).

### Spontaneous neoantigen-specific T-cell responses

Pre-existing immunity to the MUT and control WT peptides in the autologous setting was assessed by cultured interferon gamma (IFN-γ)/interleukin-5 (IL-5) ELISpot as previously described (see online supplemental material)^26^; up to five independent experiments were performed. Unstimulated peripheral blood mononuclear cells (PBMC) and PBMC expanded with peptide (reactive) were phenotypically assessed by flow cytometry. Staining used the antibodies (BioLegend): CD45-FITC (clone HI30), CD3-BV786 (clone SK7), CD4-PE (clone OKT4), CD8-PerCP-Cy5.5 (clone SK1), CD19-PerCP-Cy5.5 (clone HIB19), CD20-PerCP-Cy5.5 (clone 2H7), CD14-APC-Cy7 (clone HCD14) and human leucocyte antigen (HLA)-DR-APC (clone L243), plus CD137-PE-Cy5 (clone 4B4-1), PD1-PE/Dazzle594 (clone EH12.2H7) and CD69-PE/Dazzle594 (clone FN50) where appropriate; live/dead cell discrimination used DAPI (Sigma).^27^ Cells were acquired on a BD FACSAria Fusion and analyzed in FlowJo (v10.4.1). Bulk sorting of CD8^+^ and CD4^+^ T-cell populations (1512 to 74 875 cells) and CD8^+^ and CD4^+^ T cells with an activated phenotype (442 to 2354 cells) was as previously described.^27^

### TCR repertoire analysis

Peptide-expanded PBMC were bulk sorted directly into lysis buffer RLT (Qiagen) for subsequent RNA extraction, according to the manufacturer’s instructions. TCR-seq was performed as described previously (see online supplemental material).^28^ Sequencing data was mapped and analyzed using MIGEC software (V.1.2.9). Downstream analysis was performed using R (V.3.6.3) within RStudio (V.1.2.5033) using the tcR and immunarch packages.^29 30^ Successful TCRαβ reconstruction was achieved for all samples analyzed with (1) 94%–98% of the reads mapped and at least 0.20 million PE reads of length >150 bp for CD8^+^ and CD4^+^ T cells and (2) 98%–99% of the reads mapped with at least 0.15 million PE reads of length ≥300 bp for activated CD8^+^ and CD4^+^ T cell populations.

### MyVac vaccine

A personalized Modified Vaccinia Ankara (MVA)-based neoantigen vaccine, MyVac, was generated by Transgene, Strasbourg, France.^23^ Eighteen mutated gene sequences, each separated by a five amino acid linker (GSGSG, SGSGS, GSTSG, or SGTGS), were assembled into three fusion cassettes; fragments encoded peptides that were 29 (n=13), 20 (n=1) 30 (n=1), 31 (n=31) and 76 (n=1) amino acids in length. The fragment containing all three expression cassettes was cloned into the MVA-based vector, referred to as MVA TG19111; empty vector control was MVA TGN33.1. The expression of each polyepitope containing cassette was confirmed by Western blot analysis following infection of chicken embryo fibroblasts.

### De novo neoantigen-specific T-cell responses

The HLA-A*0201 transgenic mouse model (HHD) was used to evaluate de novo neoantigen-specific T-cell responses following vaccination with MyVac. Groups of six 8–12 week-old HHD mice were injected intravenously with 10^7^ plaque-forming units of MyVac on day 1 and 8; animals were randomly assigned to receive neoantigen vaccine (MVA TG19111) or empty vector control (MVA TGN33.1). Mice were sacrificed on day 15 and splenocytes were harvested for immunogenicity assessment by ex vivo IFN-γ ELISpot (BD Biosciences), according to the manufacturer’s instructions; ELISpots were performed with and without prior depletion of CD8^+^ T cells and/or blocking of MHCII (see online supplemental material).

### In silico prediction of MHC Class I and II-restricted neoepitopes

The patient’s four‐digit‐typed HLA class I and II alleles were identified by NHS-BT H&I (http://hospital.blood.co.uk/diagnostic-services/hi/). IEDB NetMHCpan (BA V.4.0) and NetMHCIIpan (V.3.1) were used to predict MHC class I (A/B) and Class II (DR/DQ) binding, respectively: 8–11mer epitopes with a predicted IC_50_ value ≤500 nM and 15mer epitopes with an IC_50_ value ≤1000 nM were considered high affinity binders and were regarded as candidate neoepitopes.

### Statistical analyses

Calculations were performed with GraphPad Prism V.8 software; differences between two groups used t-test for normally distributed data and significance was at 95% confidence level (p<0.05). Exact p values are provided; ns indicates not significant.

## Results

### Tumor characterization

**Figure 1:**
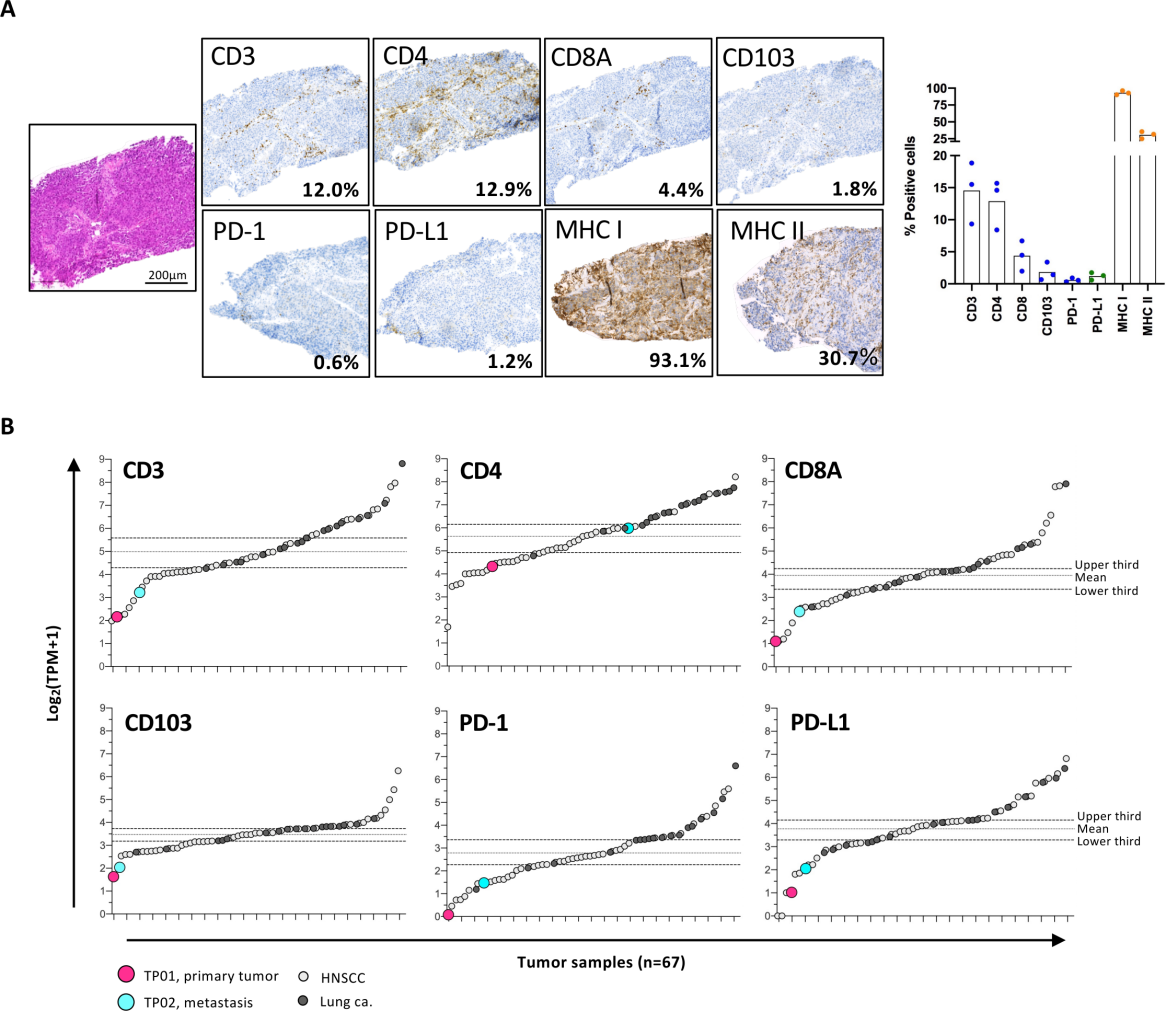
Tumor characterization. (A) Immunohistochemical staining for CD3D, CD8A, CD4, CD103, PD-1, PD-L1 and MHC class I and II was performed on primary lung tumor. Positive cells were enumerated from three independent regions using a Zeiss Axio Scan. Z1 digital slide scanner and Zeiss Zen software (V.2.6) and the QuPath bioimage analysis software (V.0.2.9-m9). (B) RNA transcripts levels (Log2 normalized TPM) in the bulk RNA-seq of primary tumor and metastasis were assessed for matched genes (as IHC above). Immune gene contextualisation used a cohort of HNSCC (n=47) and lung cancer (n=18). HNSCC, head and neck squamous cell carcinomas; IHC, immunohistochemistry; MHC, major histocompatibility complex; TPM, transcripts per million.

Immunohistochemical assessment of the primary tumor revealed a low TIL density (figure 1A); microscopically, T cells were observed distributed along the connective tissue septa and did not infiltrate the main tumor nests, consistent with an immune excluded tumor phenotype.^31^ Further, PD-1 (0.6%) and PD-L1 (1.2%) expression was low. We observed minimal loss of MHC Class I on tumor cells, while MHC Class II was predominately expressed on stromal macrophages; morphological assessment of the tumor tissue was conducted by an experienced histopathologist (GT). In parallel, we evaluated the matched gene RNA transcript levels (Log2 normalized transcripts per million, TPM) within the context of a cohort of HNSCC and lung cancer (figure 1B).^25^ For the primary tumor, TPM for CD3D, CD4 and CD8A, PD-1 and PD-L1 fell in the bottom tertile of the cohort, consistent with IHC data. Intriguingly, the RNA transcript levels detected in the metastasis, taken after Cisplatin/Pemetrexed chemotherapy and two doses of nivolumab, are slightly higher for all markers, but remain in the lowest tertile, with the exception of CD4 transcripts, which fell into the median tertile (figure 1B). Compared with other lung cancer and HNSCC cases, the expression of MHC class I and II alleles at the transcriptomic level by both the primary tumor and metastasis was low in spite of the apparent protein expression detected by IHC (online supplemental figure S1); we note a relatively higher number of RNA transcripts, particularly for MHCII, in the metastasis compared with the primary tumor.

### Genomic evaluation and tumor mutational landscape

**Figure 2:**
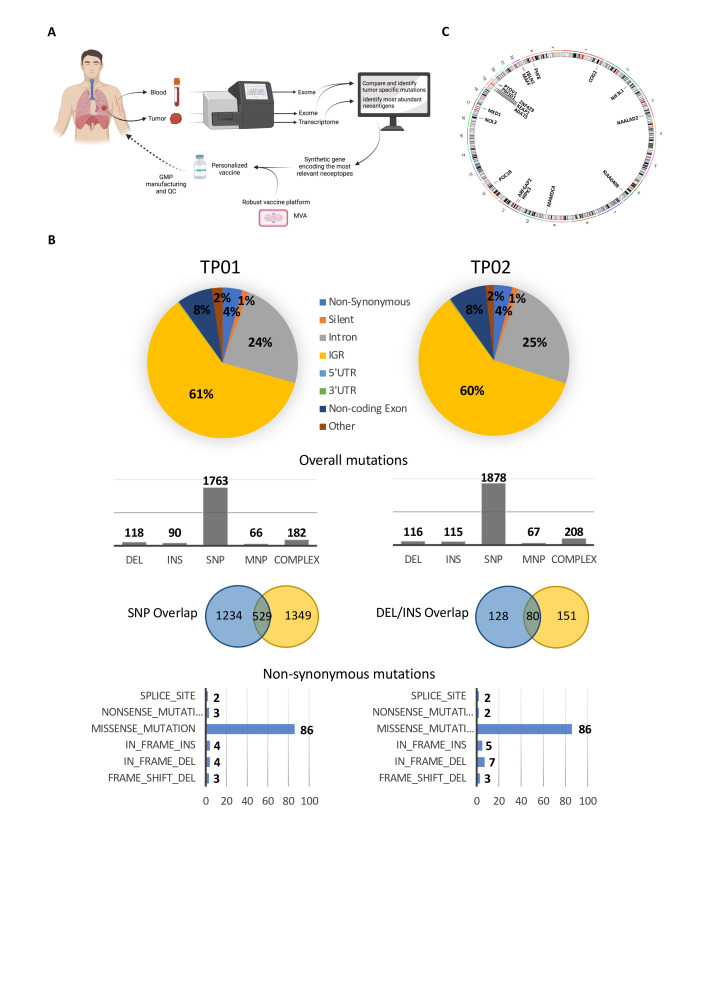
Neoantigen discovery and vaccine generation. (A) Schematic illustration of neoantigen discovery and the generation of a personalized neoantigen vaccine. Created with BioRender.com (B) WES of tumor tissue—primary lung tumor at diagnosis (TP01) and a parasternal metastasis collected +3 months following diagnosis (TP02)—and peripheral blood was performed to identify tumor-specific mutations. (C) Genomic circos plot to illustrate the chromosomal location of the 18 expressed mutated gene variants that were selected for functional testing and vaccine generation. WES, whole exome sequencing. DEL, deletion; INS, insertion; SNP, single nucleotide polymorphism; MNP, multiple nucleotide polymorphism.

WES was performed on primary lung tumor (TP01) and the parasternal metastatic tumor (TP02), together with matched whole blood to identify tumor-specific mutations as the first step towards neoantigen discovery (figure 2A). Overall, 2219 somatic mutations were identified from the primary tumor, with 2384 mutations detected from the metastasis; the mutational overlap between the two tumor sites was 17% and 22% for single bp mutations and INDELs, respectively (figure 2B). For the primary tumor and metastasis, 102 and 105 non-synonymous mutations were observed, respectively, of which approx. 50% were shared –44 missense, 9 INDELS, including three frameshifts (figure 2B and online supplemental table S2). A low TMB was demonstrated at 2.68 and 2.76 mutations per MB for TP01 and TP02, respectively. No mutations or genomic rearrangements were observed in known NSCLC driver genes, including EGFR and ALK—consistent with the diagnostic workup—KRAS, ROS1, HER2, RET, or BRAF.^32^ A MET exon 14 splice site mutation (c.3028G>A) was observed in both the primary tumor and metastasis and was associated with MET amplification. MET exon 14 mutations have been reported in 3% of non-squamous NSCLC, often in the absence of other activating mutations or gene rearrangements, and can cause exon 14 skipping, resulting in a mutant receptor leading to increased c-Met signaling and oncogenic potential.^33^

10.1136/jitc-2021-003821.supp2 Supplementary data

Selection of candidate neoantigens from the primary tumor followed filtering of the total number of non-synonymous mutations to yield mutations that were expressed at the RNA level. Twenty-three variants (1 DEL/frameshift and 22 missense mutations) were observed in 18 gene products—in four cases, the variant was observed in multiple gene isoforms—and included genes that were associated with transcription regulation, cell proliferation and apoptosis (summarized in table 1; figure 2C). A number of genes are known (eg, KEAP1—Cancer Gene Census gene, tier 1) or strongly indicated (eg, ZNF429—putative cancer gene, tier 2) to have a role in cancer, or have been implicated in cancer using mouse models, according to the Catalog of Somatic Mutations in Cancer database; similarly, mutations in NIF3L1 (c344G>T) and KIAA0408 (c2063G>A) have been previously observed in samples from endometrioid carcinoma and colorectal adenocarcinoma, respectively. All 18 mutated gene sequences were selected for immunogenicity testing and vaccine construction.

**Table 1:** Gene mutations selected for functional validation

Gene symbol	Entrez gene name	Somatic mutation		Effect	Subcellular localisation	GO biological process*
		BP	AA			
*COG2*	Component of oligomeric golgi complex 2	c.257T>C	p.Leu86Ser	Missense	G, C	Protein transport
*HIPK3†*	Homeodomain interacting protein kinase 3	c.83T>C	p.Val28Ala	Missense	N, C	Transcription; Transcription regulation; Apoptosis
*ARFGAP2*†	ADP ribosylation factor GTPase activating protein 2	c.14C>G	p.Pro5Arg	Missense	PM, G, C	Protein transport; ER-Golgi transport
*POC1B*†	POC1 centriolar protein B	c.29G>A	p.Arg10Lys	Missense	CS	Cell proliferation; Cilium biogenesis/degradation
*NOL3*	Nucleolar protein 3	c.23C>T	p.Pro8Leu	Missense	N, C, M	Apoptosis; mRNA processing; mRNA splicing
*MED1*†	Mediator complex subunit 1	c.2099C>A	p.Pro700Gln	Missense	N	Transcription; Transcription regulation
*ADAT3*	Adenosine deaminase, tRNA specific 3	c.832C>G	p.Leu278Val	Missense	N	tRNA processing
*KEAP1*†	Kelch like ECH associated protein 1	c.1824delC	p.Val608fs	Frameshift	N, C, CS	Transcription; Transcription regulation; Ubl conjugation pathway
*ZNF429*†	Zinc finger protein 429	c.220C>A	p.Pro74Thr	Missense	N	Transcription; Transcription regulation
*PIH1D1*	PIH1 domain containing 1	c.459G>A	p.Met153Ile	Missense	N	Transcription; Transcription regulation
*PTOV1*	PTOV1, extended AT-hook containing adaptor protein	c.47C>T	p.Ser16Leu	Missense	N, PM	Transcription; Transcription regulation
*NIF3L1*	NGG1 interacting factor 3 like 1	c.344G>T‡	p.Arg115Leu	Missense	N, M	Transcription; Transcription regulation; Neuron differentiation
*MAFF*	MAFF bZIP transcription factor F	c.98C>T	p.Ser33Leu	Missense	N, M	Transcription; Transcription regulation; Stress response
*FBLN1*†	Fibulin 1	c.213G>T	p.Met71Ile	Missense	E	Host-virus interaction
*NAALADL2*†	N-acetylated alpha-linked acidic dipeptidase like 2	c.64G>A	p.Asp22Asn	Missense	PM	Carboxypeptidase; Dipeptidase; Hydrolase; Metalloprotease; Protease
*KIAA0408*	KIAA0408	c.2063G>A‡	p.Arg688Gln	Missense	Unknown	Uncharacterized
*MAMDC4*	MAM domain containing 4	c.1486G>C	p.Glu496Gln	Missense	PM, N, E	Protein transport
*PHF8*	PHD finger protein 8	c.115G>C	p.Glu39Gln	Missense	N	Cell cycle; Transcription; Transcription regulator

*As defined by Uniprot, https://www.uniprot.org.

†Genes associated with cancer phenotypes, as reported by the COSMIC database.

‡Mutations observed previously in cancer phenotypes.

C, cytosol; COSMIC, Catalog of Somatic Mutations in Cancer; CS, cytoskeleton; E, extracellular space; G, golgi apparatus; M, mitochondrion; N, nucleus; PM, plasma membrane.

NGS data were interrogated to determine the patient’s HLA class I and class II alleles to four-digits (online supplemental table S3).

### Validation of candidate neoantigens in the autologous setting

**Figure 3:**
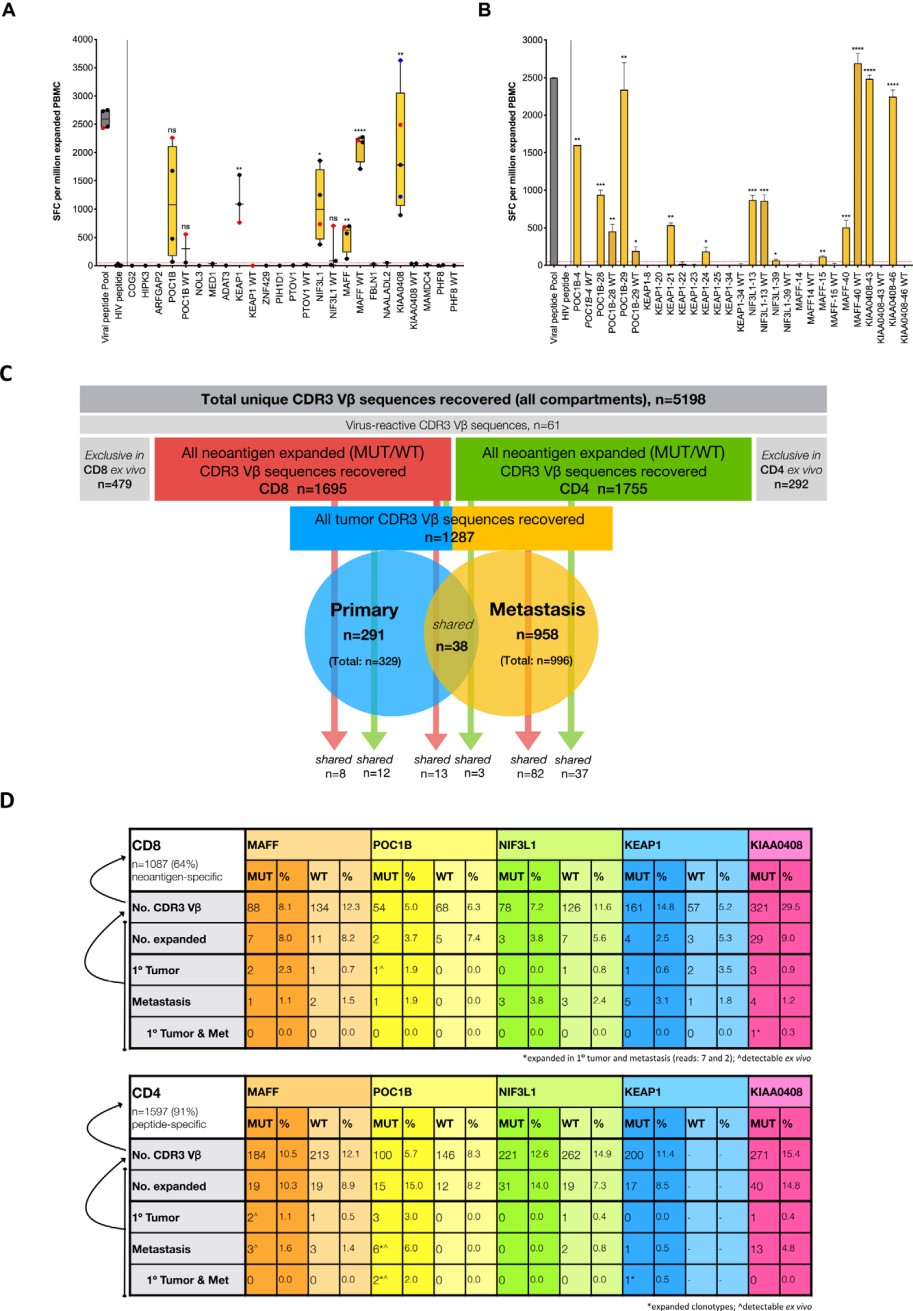
Evaluation of spontaneous neoantigen-specific T-cell responses and tracking TCR clonotypes in blood and tumor tissue. (A) Autologous T-cell responses were assessed by a 13-day cultured IFN-γ ELISpot against MUT and equivalent WT long peptide pools. Data are shown for up to five assay replicates. An HLA-A*02-restricted viral peptide pool and HIV peptide served as positive and negative control, respectively. Assays denoted by a red dot represent those used for individual peptide screening (panel B) and FACS sorting of CD8^+^ and CD4^+^ T cells for subsequent TCR-seq (panel C, D). Assays denoted by a blue dot represent those used for FACS sorting of CD8^+^ and CD4^+^ T cells with an activated phenotype (CD137, CD69 and PD1) for subsequent TCR-seq. Red dashed lines denote the cut-off for a positive IFN-γ response based on mean SFC plus 2 x StDev for irrelevant (HIV) peptide control. An unpaired t-test was performed for each test group (neoantigen MUT or WT with positive IFN-γ response) against irrelevant HIV peptide control; significant p values: **p=0.0026 (KEAP1), *p=0.0175 (NIF3L1), **p=0.0093 (MAFF), ****p<0.0001 (MAFF WT) and **p=0.0087 (KIAA0408). (B) T-cell responses to individual long peptides were assessed. An unpaired t-test was performed for each test group (neoantigen MUT or WT with positive IFN-γ response) against irrelevant HIV peptide control; significant p values: **p=0.0024 (POC1B-4), ***p=0.0007 (POC1B-28), **p=0.0085 (POC1B-28 WT), **p=0.0031 (POC1B-29), *p=0.0283 (POC1B-29 WT), **p=0.0018 (KEAP1-21), *p=0.0325 (KEAP1-24), ***p=0.0002 (NIF3L1-13), ***p=0.0006 (NIF3L1-13 WT), *p=0.0288 (NIF3L1-39), **p=0.0096 (MAFF-15), ***p=0.0010 (MAFF-40), ****p<0.0001 (MAFF-40 WT), ****p<0.0001 (KIAA0408-43) and ****p<0.0001 (KIAA0408-46). (C) Schematic to illustrate the total CDR3 Vβ sequences recovered by TCR-seq from the primary tumor, the metastasis and the blood, both of unstimulated PBMC ex vivo and of PBMC after stimulation and expansion with virus-specific and neoantigen-specific peptide. (D) Schematic to illustrate expansion and sharing (with tumor) for neoantigen-specific CD8^+^ and CD4^+^ TCR clonotypes. The curved arrows represent the basis of percentage calculation. Expanded TCR clonotypes are marked with an asterix (*); TCR clonotypes that were also detected directly ex vivo are marked with a caret (^∧^). PBMC, peripheral blood mononuclear cells; MUT, mutated; WT, wild type; SFC, spot forming cells.

We assessed spontaneous T-cell reactivity to candidate neoantigens using a cultured IFN-γ ELISpot; autologous PBMC were stimulated with neoantigen-specific long peptides (online supplemental table S1) in the presence of IL-2 for 13 days. Five of 18 (28%) MUT gene variants were found to be reactive: POC1B, KEAP1, NIF3L1, MAFF and KIAA0408 (figure 3A). Of note, T-cell responses were not detectable by cultured IL-5 ELISpot nor by direct ex vivo IFN-γ ELISpot (data not shown). T-cell responses were exclusively directed towards the MUT gene sequence for KEAP1 and KIAA0408, while the WT sequence elicited responses in the remaining cases to a lesser (POC1B and NIF3L1) or greater (MAFF) magnitude. Further evaluation revealed that reactive neoepitopes were most likely to be contained within KEAP1-21/24, NIF3L1-13 and MAFF-40 peptides (figure 3B); for KEAP1, T-cell reactivity was focused on the non-templated sequence arising through the frameshift deletion. Reactivity to each of the associated peptides was observed for POC1B (−4/28/29) and KIAA0408 (−43/46).

### Assessment of TCR repertoire

We performed TCR-seq on whole tumor (snap frozen tissue): (1) primary tumor and (2) metastasis, and on PBMC from the blood, on bulk sorted CD8^+^ and CD4^+^ T cells: (3) unstimulated PBMC directly ex vivo, and on PBMC expanded with (4) positive control viral peptides and (5) an independent assessment of PBMC expanded with MUT (reactive) synthetic peptides and corresponding WT synthetic peptides, with the exception of KIAA0408 WT, where there were not enough cells remaining after ELISpot for TCR-seq analysis. Globally, a total of n=5198 unique CDR3 Vβ sequences were recovered (figure 3C). PBMC stimulated with an HLA-A*02-restricted viral pool was used as a positive control and offered the opportunity to evaluate the frequency of TCRs reactive with epitopes from common viruses; 61 unique CDR3 Vβ sequences were identified. Intriguingly, 41 virus-reactive CDR3 Vβ sequences were also recovered in neoantigen-specific peptide-expanded PBMC across all cultures (MUT and WT peptides), suggesting either clonal expansion in the blood pre-culture, non-specific bystander activation of virus-reactive T cells or, perhaps less likely, cross-reactivity between tumor neoantigens and viral peptides. Virus-reactive TCR clonotypes could also be recovered from the tumor (primary tumor n=1, metastasis n=5 or both sites n=1) and from the blood directly ex vivo (n=6, all CD4, some of which were also observed in the primary tumor (n=1) or metastasis (n=2)). The virus-reactive TCR clonotypes were excluded from further analysis.

As our interest was in defining tumor antigen-reactive TCR clonotypes, we evaluated CDR3 Vβ sequences recovered by TCR-seq from sorted CD8^+^ (total n=1695) and CD4^+^ (total n=1755) T cells from PBMC expanded with neoantigen-specific peptide. Of note, the overlap between CD8 and CD4 TCR clonotypes was n=152 or 4.6%, in keeping with a previous study demonstrating a 4.7% overlap of Vβ chains when assessed independently from their partner Vα chains and may reflect technical aspects of FACS sorting (98% purity), although true interconversion of CD8^+^ and CD4^+^ T cells has been reported.^34–36^ There was evidence of TCR clonotype sharing between cultures stimulated with different neoantigen-specific peptides (online supplemental figure S2). Moreover, if we consider CDR3 Vβ sequences recovered following expansion with MUT peptide and the corresponding WT peptide, we again observe TCR clonotype sharing, which is more pronounced for CD8^+^ T cell populations where ca. 10%–25% of TCRs are shared compared with less than 4% for CD4^+^ T cells (online supplemental table S4); up to 4% of the shared CD8^+^ TCR clonotypes are virus-specific, while no viral TCRs are detected for CD4^+^ T cells. Nevertheless, the majority of unique CDR3s identified were specific to a particular neoantigen stimulation: 64% and 91% for CD8^+^ and CD4^+^ T cells, respectively. We also evaluated CDR3 Vβ sequences recovered from sorted CD8^+^ (total n=499) and CD4^+^ (total n=356) T cells from PBMC ex vivo. We identified a subset of n=479 (CD8^+^ T cells) and n=292 (CD4^+^ T cells) unique CDR3s that were not detected in any of the in vitro stimulation experiments, nor in the tumor tissue (figure 3C). As we cannot allocate antigen-reactivity, we removed these CDR3s from further evaluation and interpretation. Nine CDR3s from CD8^+^ T cells and 23 CDR3s from CD4^+^ T cells ex vivo were observed in PBMC cultured with neoantigen-specific peptides, suggesting the clonal expansion of T cells in the blood, leading to a detectable signal in the neoantigen-stimulated cultures (online supplemental figure S3). Further, 12 CDR3s from CD8^+^ T cells and 23 CDR3s from CD4^+^ T cells ex vivo were also recovered in tumor tissue. Overall, TCR-seq analysis of the primary tumor and metastasis demonstrated n=291 and n=958 unique CDR3s, respectively, with a further n=38 shared across both sites (figure 3C). As these data were generated from bulk analyses of tumor RNA, we could not a priori attribute them to CD8^+^ or CD4^+^ T cells. However, allocation was possible where we recovered the same TCR from CD8^+^ and CD4^+^ T-cell populations sorted by flow cytometry. Overall sharing of TCR clonotypes from neoantigen-specific peptide expanded cultures and tumor tissue was 12% (n=21 CD8 and n=15 CD4) and 14% (n=95 CD8 and n=40 CD4) for primary tumor and metastasis, respectively. A small proportion (<1% CD8 and 2% CD4) of TCR clonotypes observed ex vivo were evident in both expanded cultures and tumor.

A focused analysis of TCR clonotypes was undertaken for each individual neoantigen, both MUT and equivalent WT, to determine the degree of sharing with the primary tumor, metastasis or both (figure 3D). For this purpose, we define neoantigen-specific TCRs as CDR3 Vβ sequences recovered exclusively from PBMC cultures stimulated with an individual neoantigen peptide, and which are not observed in any other culture condition, that is, cultures stimulated with the equivalent WT peptide or any other neoantigen MUT/WT peptides; we acknowledge that we have not demonstrated neoantigen-specificity functionally and therefore the TCRs should be considered ‘potentially’ neoantigen-specific. For expanded CD8^+^ T cells, n=1087 (of 1695 total CDR3 Vβ sequences, 64%) neoantigen-specific TCR clonotypes were observed; for expanded CD4^+^ T cells, n=1597 (of 1755 total CDR3 Vβ sequences, 91%) neoantigen-specific TCR clonotypes were observed. Stimulation with KIAA0408 MUT contributed the largest proportion of neoantigen-specific TCR clonotypes for CD8^+^ (30%) and CD4^+^ (15%) T cells (figure 3D and online supplemental table S5). Sharing of at least one neoantigen-specific TCR clonotype with the primary tumor, metastasis or both, was evident for the majority of neoantigens (online supplemental table S6). This included TCRs specific for WT peptides and did not appear to be dependent on WT reactivity—similar numbers of TCRs were observed in the tumor for WT peptides that induced no/low IFN-γ-secretion in an ELISpot (POC1B, NIF3L1 and KEAP1) compared with MAFF, where reactivity was ca. 4 x greater for WT than MUT peptide (figure 3D). The greatest sharing was also observed for KIAA0408 (CD8 and CD4), with one KIAA0408 MUT-specific CD8 TCR clonotype found expanded in the primary tumor (n=7) and metastasis (n=2); clonotype sharing with both the primary tumor and metastasis was also evident for POC1B MUT (CD4) and KEAP1 MUT (CD4). Furthermore, n=9 neoantigen-specific TCR clonotypes that were shared with tumor tissue were also detected in the blood ex vivo, including n=1 (CD8) and n=6 (CD4) clonotypes for POC1B MUT and n=2 (CD4) clonotypes for MAFF MUT; no WT-specific TCR clonotypes were observed in the blood ex vivo.

10.1136/jitc-2021-003821.supp3 Supplementary data

10.1136/jitc-2021-003821.supp4 Supplementary data

For KIAA0408 neoantigen, we performed additional TCR-seq analysis on cells expanded with the MUT peptide in two separate experiments and then sorted the cells that expressed CD137, and in the second experiment, one or more of the activation markers, CD137, CD69 or PD1. TCR Vβ segments that had been previously identified uniquely for CD8^+^ and CD4^+^ T cells following stimulation with KIAA0408 MUT peptide were evident in both assays, and were more frequent for activated CD8^+^ T cells: n=5 (CD8^+^) and n=1 (CD4^+^) (online supplemental figure S5). Overall, TCR Vβ segments identified in the activated T cell populations were also evident in the primary tumor (n=5 for CD4^+^) and, more commonly, the metastasis (n=5 for CD8^+^ and n=12 for CD4^+^) consistent with our initial findings for KIAA0408-expanded bulk CD8^+^ and CD4^+^ T cell populations; this included two TCRs that were observed in the previous KIAA0408 MUT dataset and the metastasis, n=1 from CD8^+^CD137^+^ T cells (sample 1) and n=1 for CD8^+^CD137^+^ and/or CD69/PD1^+^ T cells (sample 3). Intriguingly, a number of KIAA0408 specific TCRs recovered from activated T cells were also recovered from blood directly ex vivo (n=3 CD8^+^ and n=13 CD4^+^), perhaps suggesting a substantial clone size even without in vitro expansion. Taken together, these data support our assumption that the TCR clonotypes we describe are neoantigen-specific.

### Preclinical testing of a personalized neoantigen vaccine, MyVac

**Figure 4:**
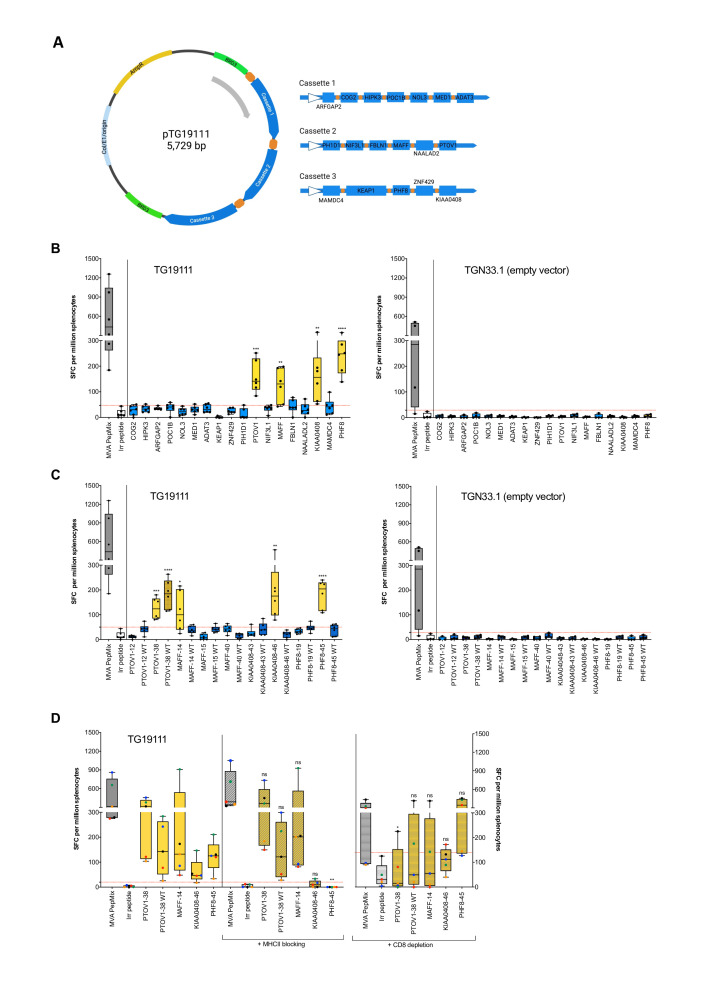
Preclinical testing of a personalized neoantigen vaccine, MyVac. (A) Schematic illustration of the personalized, MVA-based vaccine, MyVac (MVA TG19111), incorporating 18 mutated gene sequences; an empty vector (MVA TGN33.1) served as a negative control. Created with BioRender.com (B) De novo T-cell responses were assessed by ex vivo IFN-γ ELISpot against mut long peptide pools; splenocytes were harvested on day 15 post-1st vaccination. An unpaired t-test was performed for each test group (neoantigen MUT with positive IFN-γ response) against irrelevant peptide control; significant p values: ***P=0.0003 (PTOV1), **p=0.0033 (MAFF); **p=0.0087 (KIAA0408) and ****p<0.0001 (PHF8). (C) T-cell responses to individual long peptides were assessed. An unpaired t-test was performed for each test group (neoantigen MUT or WT with positive IFN-γ response) against irrelevant peptide control; significant p values: ***p=0.0002 (PTOV1-38), ****p<0.0001 (PTOV1-38 WT), *p=0.0146 (MAFF-14), **p=0.0088 (KIAA0408-46) and ****p<0.0001 (PHF8-45). (D) Antibody blocking of MHCII and depletion of CD8^+^ T cells was performed to elucidate MHC class restriction. An unpaired t-test was performed for each test group (neoantigen MUT or WT with positive IFN-γ response) without vs with (I) prior MHCII blocking or (II) prior CD8 depletion; significant p values: **p=0.0022 (PHF8-45, on MHCII blocking) and *p=0.0338 (PTOV1-38, on depletion of CD8^+^ T cells). For panels B, C, data are shown for n=6 (MVA TG19111 neoantigen vaccine, left) or n=4 (empty vector control, right) mice; for panel D, data are shown for n=5 mice, where data for individual mice can be identified by color (red, blue, green, orange and black). Red dashed lines denote the cut-off for a positive IFN-γ response based on mean SFC plus 2 x SD for irrelevant peptide control. MHC, major histocompatibility complex; MVA, Modified Vaccinia Ankara; ns, not significant; SFC, spot forming cells.

A personalized neoantigen vaccine, MyVac, was designed to target the 18 mutated genes (figure 4A). In keeping with the patient’s expression of HLA-A*0201 allele, we vaccinated HLA-A*02-restricted transgenic HHD mice and could demonstrate this was sufficient to induce strong neoantigen-specific T-cell responses, as measured by ex vivo IFN-γ ELISpot against neoantigen-specific long peptides (online supplemental table S1). T-cell reactivity was observed to four of 18 (22%) MUT gene variants: PTOV1, MAFF, KIAA0408, and PHF8 (figure 4B), of which two were also observed in the autologous setting (figure 3A and table 2). Reactivities could be attributed to at least one dominant responding peptide: PTVO1-38, MAFF-14, KIAA0408-46 and PHF8-45 (figure 4C). No responses were observed to the empty control vector (figure 4B and C, right panels). Moreover, T cells were responding exclusively to the MUT peptides, with the exception of PTOV1-38 in which reactivity to the corresponding WT peptide was also observed. In order to determine if the T cells were specific for HLA-A*02 epitopes or murine MHCII epitopes, blocking of MHCII or depletion of CD8^+^ T-cell subsets (in the absence of HHD-specific blocking antibody) was performed (figure 4D). The anti-IA/IE antibody was able to block the activation of CD4^+^ T cells to a well-defined MHC class II epitope from tetanus toxin, p30 (online supplemental figure S6). T-cell responses to PHF8-45 (p=0.0022) and KIAA0408-46 (ns) were reduced on MHCII blocking, while all other peptides remained unaffected, suggesting that responses are CD4-mediated; although not statistically significant, KIAA0408-specific IFN-γ SFC were reduced by 64%–100% at the level of individual mice (n=5). Conversely, an HLA-A*02-restricted CD8-mediated T-cell response for PTOV1-38 (p=0.0338) was confirmed on the depletion of CD8^+^ T cells, where T-cell responses were diminished. Further, responses to the corresponding PTOV1-38 WT and MAFF-14 peptides were also decreased on depletion of CD8^+^ T cells, but this did not reach significance; in both cases, 4 out of 5 mice showed a reduction in IFN-γ SFC by 20%–100% and 36%–100% for PTOV1-38 WT and MAFF MUT, respectively. Taken together, MyVac was able to elicit strong neoantigen-specific CD8^+^ (HLA-A*02-restricted) and CD4^+^ (murine) T-cell responses.

**Table 2:** Summary of neoantigen-specific T-cell responses in the autologous and HHD setting

	Autologous			HHD			
Gene Symbol	Reactivity	MUT specific	Positive peptide	Reactivity	MUT specific	Positive peptide	MHC restriction
*POC1B*	Yes	No	POC1B-4, 28, 29	No	–	–	–
*KEAP1*	Yes	Yes	KEAP1-21, 24	No	–	–	–
*PTOV1*	No	–	–	Yes	No	PTOV1-38	MHCI
*NIF3L1*	Yes	No	NIF3L1-13	No	–	–	–
*MAFF*	Yes	No	MAFF-40	Yes	Yes	MAFF-14	MHCI
*KIAA0408*	Yes	Yes	KIAA0408-43, 46	Yes	Yes	KIAA0408-46	MHCII
*PHF8*	No	–	–	Yes	Yes	PHF8-45	MHCII

HHD, human leukocyte antigen A*0201 transgenic mouse model; MHC, major histocompatibility complex; MUT, mutation.

### In silico neoepitope prediction

Our approach to use long peptides allows for the expansion of both CD8^+^ and CD4^+^ T-cell responses.^37^ Following the identification of the patient’s HLA genotype by NGS (online supplemental table S3), we next applied the in silico epitope prediction algorithm NetMHCpan to identify likely MHC class I binding neoepitopes (online supplemental table S7). In keeping with the observed T-cell reactivity in the HHD mouse and the autologous setting, MAFF contains high affinity MUT-specific HLA-A*02:01 binders within MAFF-14/40 peptides. Further, high affinity MAFF WT-specific HLA-A*02:01 epitopes were predicted, consistent with reactivity in the autologous setting; however, since reactivity was absent in the HHD setting it is probable that the WT reactivity observed originates from an alternative MHCI/II HLA allele, one candidate epitope has been predicted for HLA-B*27:05. HLA-A*02:01 epitopes with a specificity for both MUT and WT PTOV1 were predicted within peptide PTOV1-38, suggesting that although MyVac is able to induce neoantigen-specific CD8^+^ T cells in HHD mice, in the autologous setting these may have been subject to deletion during clonal selection as reflected in a lack of observed T-cell reactivity. A number of potential epitopes were identified for KIAA0408, involving all the patient’s class I HLA alleles; however, data from the HHD model suggests that the response to KIAA0408 is not HLA-A2-restricted and, therefore, if MHCI-mediated, must act through an alternative allele, that is, HLA-A*32:01, HLA-*B15:01 or HLA-*B27:05. A stretch of novel sequence introduced through the DEL/frameshift mutation has generated a number of potential epitopes for KEAP1, including within KEAP1-21 and KEAP1-24 peptides, that are specific for HLA-A*02:01, as well as the patient’s other class I HLA alleles. Similarly, potential epitopes were predicted for POC1B and NIF3L1, consistent with autologous T-cell reactivity for MUT and WT peptides. No MHCI binders were predicted for PHF8 MUT for any of the four HLA-A/B alleles in keeping with a likely murine MHCII restriction. Using the NetMHCIIpan algorithm, a number of high affinity MHC class II binding epitopes were predicted for reactive MUT genes and prediction of H2-IA binding identified murine CD4 epitopes for both KIAA0408 and PHF8, consistent with HHD blocking ELISpot data.

## Discussion

We used exome and RNA-seq to identify tumor-specific neoantigens that could be exploited by vaccination for a patient with lung cancer. In our study, we observed a low TMB for both the primary tumor and the metastasis, and, consequently, identified a low number of potential neoantigens; 23 somatic mutations involving 18 gene products were found to be expressed in the tumor transcriptome. Since smoking is significantly associated with a high TMB in NSCLC, this is consistent with the patient’s known never-smoker status.^38^ It is widely recognized that cancers, including lung cancer, with a high TMB and, therefore, higher numbers of predicted neoantigens, exhibit an improved clinical benefit among patients receiving immunotherapy, and that neoantigen-specific T cells play a vital role, conferring a superior objective response rate and PFS.^2 11 12^ In this patient, treatment with anti-PD-1 did not result in clinical benefit and the patient progressed rapidly; early disease progression within 3 months of starting PD-1 blockade has been observed by ca. 40% of NSCLC patients in clinical trials.^39^ Analysis of the immune cell infiltrate at the protein and RNA level demonstrated that the primary tumor had a low number of TIL, accompanied by a low expression of PD-1 and PD-L1 on T cells and tumor cells, respectively. A low TIL status is recognized as an indicator of poor prognosis in patients with cancer and is one reason for failure of anti-PD-1 and anti-PD-L1 immunotherapies.^40 41^ A low immune cell infiltrate was also evident for the parasternal metastasis, although the RNA transcript levels were slightly raised compared with the primary tumor. One notable exception was the increased number of CD4^+^ T cells in the metastasis. Treatment with chemotherapy and anti-PD-1 may have resulted in the influx of (neoantigen-specific) CD4^+^ T cells to the tumor, suggesting an active, although clinically ineffective, response. Our recent data suggest that anti-PD-1 may also activate an inhibitory population of T follicular regulatory cells,^42^ although the available material in our patient here did not allow us to differentiate the two treatment consequences. While mounting evidence suggests anti-tumor immunity is achieved via the activation of neoantigen-specific cytotoxic CD8^+^ T cells,^12^ it is increasingly recognized that CD4^+^ T cells, particularly Th1 cells, play a critical role to enhance the functions of cytotoxic CD8^+^ T cells via promotion of priming, migratory potential, killing activity and survival.^43^ Endogenous neoantigen-specific CD4^+^ T cells have been observed in patients with cancer, and some studies report that neoantigen-specific peptides induce CD4^+^ T cells more frequently than CD8^+^ T cells.^3 6 44^ Peritumoral CD4^+^ T cells have also been associated with improved prognosis in NSCLC.^45^ Equally, studies in murine models demonstrate that CD4^+^ T-cell responses to neoantigens are more prevalent and potentially more effective in anti-tumor immunity, with peptides predicted to bind MHC Class I in silico having been shown to generate chiefly CD4^+^ T cells in vivo.^15^ The expression of MHC Class II by tumor cells could afford the potential for direct recognition and engagement of tumor cells presenting tumor antigens to CD4^+^ helper T cells. Indeed, expression of MHCII on tumor cells is reported to be associated with increased lymphocytic infiltration and a better response to checkpoint blockade, with increased PFS and OS.^46^ Here, we observe minimal expression of MHC Class II on tumor cells, consistent with a low TIL density and perhaps contributing to the poor response to anti-PD-1 therapy.

A combination of low TIL, low TMB and a restricted pool of candidate neoantigens would imply that the potential for the induction of neoantigen-specific T cells by vaccination is limited for this patient. Nevertheless, using a long peptide approach designed to capture both CD8^+^ and CD4^+^ T cells, we were able to demonstrate spontaneous reactivity of autologous PBMC to 5/18 neoantigens. Reactivities were generally of low frequency and required a period of in vitro stimulation for detection by IFN-γ ELISpot, suggesting the expansion of pre-existing memory CD8^+^ and/or CD4^+^ T cells. Evaluation of immunogenicity of selected neoantigens in the human transgenic HHD mouse model and using a MyVac vaccination approach similarly showed the induction of de novo T-cell responses to 4/18 putative neoantigens, and indicated that reactivity to mutated MAFF in the autologous setting was most likely MHC Class I-mediated and therefore HLA-A*02-restricted; a number of HLA-A*02-restricted epitopes could be predicted in silico. Candidate MHC Class I and Class II epitopes were also identified for the other reactive mutant genes. It is possible that anti-PD-1 treatment augmented the release of neoantigen-specific T cells into the peripheral blood, enabling the capture of these reactivities in our evaluation; PBMC were harvested following one dose of Nivolumab. An increase in PD-1^+^CD8^+^ T cells with an effector-like phenotype has been documented in the peripheral blood of NSCLC patients receiving anti-PD-1 therapy, which was shown to follow the first or second dose. However, this appears to be associated predominantly with those patients experiencing clinical benefit.^47 48^ Similarly, CD4^+^ T cells in the peripheral blood have been reported to correlate with clinical responses to PD-1 blockade in patients with melanoma.^49^ The exchange of T cell clones between the tumor tissue and peripheral blood represents a key correlate of pathologic response to anti-PD-1 therapy in both advanced disease and the neoadjuvant setting.^48 50^ TCR-seq analysis of IFN-γ-positive PBMCs that had been cultured with neoantigen-specific peptides revealed CD8^+^ and CD4^+^ TCR clonotypes that were also detectable, and in some cases expanded, in tumor tissue. Our data suggest further, that in this particular patient, chemotherapy plus anti-PD-1 treatment may have increased the level of anti-tumor immune responses, visible in increases of transcripts for CD3, CD8, CD4 and PD-1, consistent with a downstream increase of expression in both MHC molecules and of PD-L1. Nonetheless treatment was insufficient to trigger clinical benefit. Vaccination with a personalized approach as evaluated here may overcome some of these hurdles and clinical evaluation has been initiated (NCT04183166). We note additionally, that in patients treated with anti-PD-L1, the number of CD8^+^CD103^+^ T cells were predictive for benefit^51^ and it will therefore be important to assess whether vaccination also can expand CD8^+^ tissue-resident memory cells in patients such as ours, where the pre-existing number was low. Finally, it is likely that to allow effector function to occur, a strategy to reverse immune exclusion will be needed. One such option is thorough the modulation of cancer-associated fibroblasts^31^ and a number of strategies are in preclinical and clinical evaluation to achieve this.

In conclusion, we have demonstrated that even from a limited neoantigen pool, and for patients with whom the tumor microenvironment is less favorable, it is possible to identify neoantigens that can stimulate neoantigen-specific T cells in vivo. Targeting such neoantigens by vaccination, for example using an MVA-based vaccine as presented here, may afford an attractive option for personalized therapy in NSCLC, in combination with anti-PD-1/PD-L1 immunotherapies and strategies to overcome immune exclusion of T cells from the tumor.

## Data Availability

Data are available in a public, open access repository. All data relevant to the study are included in the article or uploaded as online supplemental information. Sequencing data have been deposited in the Gene Expression Omnibus under accession number GSE179879.
