# Supplementary material for: Targeting the tumor mutanome for personalized vaccination in a TMB low non-small cell lung cancer
Source: J Immunother Cancer. 2022 Mar 30;10(3):e003821. doi: 10.1136/jitc-2021-003821 (PMC8971766; doi:10.1136/jitc-2021-003821)
Supplement: Supplementary data [file jitc-2021-003821supp001.pdf]

SUPPLEMENTARY MATERIAL

SUPPLEMENTARY METHODS

Gene alignment and variant calling Exome sequencing reads were aligned to the National Center for Biotechnology Information human genome reference sequence (GRCh37/hg19) using SpeedSeq/Burrows-Wheeler Aligner software (v0.7.13). Total mapped reads in WES were 111M (99.5%), 130M (99.5%) and 83M (99.6%) for TP01, TP02 and germline, respectively. RNA-seq reads were aligned using Spliced Transcripts Alignment to a Reference (v2.6); total mapped reads were 91M (94.5%) and 101M (90.9%) for TP01 and TP02, respectively. Putative somatic variants were identified using SpeedSeq Somatic (v0.1.0), annotated with SnpEff (v4.1a) and filtered using the SQLite database. Filtering was according to the following criteria: (i) sequencing depth ≥10, (ii) genotype quality score ≥20, (iii) normal genotype is the homozygous reference, (iv) tumor genotype is NOT the homozygous reference, (v) somatic variant occurs in the coding region, (vi) ≥1 observation of the variant in tumor RNA-seq and (vii) zero observations of the variant in normal exome sequence.

Cultured IFN-γ ELISpot (autologous setting) Mononuclear cells previously isolated from leukapheresis product were stimulated at $2x10^6$/well in 24-well plates with MUT or WT long peptide (individual peptide and/or pools) together with recombinant interleukin-2 (IL-2; R&D Systems Europe Ltd.) at a final concentration of 5µg/mL and 20IU/mL, respectively, and incubated at 37°C with 5% CO_2; final volume was 2mL. Medium containing additional IL-2 (20IU/mL) was refreshed on days 4, 6, 8 and 11 and on day 13 cells were harvested. Expanded cells ($1x10^5$cell/well) were incubated in triplicate with peptide (as per the prior *in vitro* stimulation) at 5µg/mL final concentration for ca. 22 hours at 37°C in 5% CO_2; phytohemagglutinin (PHA; Sigma-Aldrich Company Ltd.) and an HLA-A*02-restricted viral peptide pool consisting of 9mers from CMV [NLVPMVATV], EBV [GLCTLVAML], FLU [GILGFVFTL] and measles [KLWESPQEI] and an irrelevant HLA-A*02-restricted HIV [ILKEPVHGV] peptide were used as positive and negative controls, respectively (Peptide Protein Research Ltd.). IFN-γ/IL-5 ELISpot was performed according to the manufacturer's instructions (MABTECH). Spot forming cells (SFC) were counted using the AID ELISpot plate reader system ELR04 and software (AID Autoimmun Diagnostika GmbH) and positivity calling for ELISpot data used the runDFR(x2) online tool (http://www.scharp.org/zoe/runDFR/).

TCR-seq Five ng of total RNA was reverse transcribed with TCRα and TCRβ gene-specific primers (see table below), followed by PCR amplification. The reaction volume was divided into 2 equal parts and TCRα and TCRβ were amplified separately in a 2nd PCR amplification step. After amplification, libraries were prepared using True-Seq nano DNA library kit (Illumina), pooled and sequenced on the MiSeq Illumina platform to obtain 150-bp paired-end reads.

McCann K, et al. J Immunother Cancer 2022; 10:e003821. doi: 10.1136/jitc-2021-003821

Amplification Step	Primer ID	Sequence
Reverse transcription	TCR-TSO	AAGCAGUGGTAUCAACGCAGAGUNNNNUNNNNUNNNNUCTTrGrGrG
	ACR-RT	GTCTAGCACAGTTTTGTC
	BCR-RT	GTATCTGGAGTCATTGA
1st PCR	M1ss	AAGCAGTGGTATCAACGCA
	ACR-Amp	GTCACTGGATTTAGAGTC
	BCR-Amp	TGCTTCTGATGGCTCAAACAC
2nd PCR	M1s_701	NNNNTAAGGCGACAGTGGTATCAACGCAGAG
	M1s_702	NNNNCGTACTAGCAGTGGTATCAACGCAGAG
	M1s_703	NNNNAGGCAGAACAGTGGTATCAACGCAGAG
	M1s_704	NNNNTCCTGAGCCAGTGGTATCAACGCAGAG
	M1s_705	NNNNGGACTCCTCAGTGGTATCAACGCAGAG
	M1s_706	NNNNTAGGCATGCAGTGGTATCAACGCAGAG
	M1s_707	NNNNCTCTCTACCAGTGGTATCAACGCAGAG
	M1s_710	NNNNCGAGGCTGCAGTGGTATCAACGCAGAG
	M1s_711	NNNNAAGAGGCACAGTGGTATCAACGCAGAG
	M1s_712	NNNNGTAGAGGACAGTGGTATCAACGCAGAG
	M1s_714	NNNNGCTCATGACAGTGGTATCAACGCAGAG
	M1s_715	NNNNATCTCAGGCAGTGGTATCAACGCAGAG
	TCR_A_502	NNNNCTCTCTATGGGTCAGGGTTCTGGATAT
	TCR_A_503	NNNNTATCCTCTGGGTCAGGGTTCTGGATAT
	TCR_A_505	NNNNGTAAGGAGGGGTCAGGGTTCTGGATAT
	TCR_A_506	NNNNACTGCATAGGGTCAGGGTTCTGGATAT
	TCR_A_507	NNNNAAGGAGTAGGGTCAGGGTTCTGGATAT
	TCR_A_508	NNNNCTAAGCCTGGGTCAGGGTTCTGGATAT
	TCR_A_510	NNNNCGTCTAATGGGTCAGGGTTCTGGATAT
	TCR_A_511	NNNNTCTCTCCGGGGTCAGGGTTCTGGATAT
	TCR_B_513	NNNNTCGACTAGACACSTTKTTCAGGTCCTC
	TCR_B_515	NNNNTTCTAGCTACACSTTKTTCAGGTCCTC
	TCR_B_516	NNNNCCTAGAGTACACSTTKTTCAGGTCCTC

HHD mice HHD mice express a transgenic monochain MHC class I molecule in which the C-terminus of human β2-microglobulin is covalently linked to the N-terminus of a chimeric heavy chain consisting of α-1 and α-2 domains of the human HLA-A*0201 molecule fused to the α-3 domain from the mouse H-2D^b molecule. HHD mice are capable only of HLA-A*0201-restricted Class I responses due to disruption of the H-2D^b and mouse β2-microglobulin genes; murine MHC Class II responses remain intact. HHD mice were bred in-house; mice were obtained from Prof. F.A. Lemonnier, Pasteur Institute, Paris, France.[1] All experimental procedures were conducted in accordance with the United Kingdom Home Office Guidelines under the Animals (Scientific Procedures) Act 1986, under Project License P8969333C.

Ex *vivo* IFN-γ ELISpot (HHD setting) HHD mice were humanely euthanized and splenocytes were isolated by density gradient centrifugation using Lymphoprep™ (Axis-Shield, Alere Technologies AS). Splenocytes at 2.5x10^5 cells/well were incubated in triplicate with MUT or WT long peptide (individual peptides and/or pools) at 5µg/ml for ca. 20 hours at 37°C in 5% CO_2; MVA 189R PepMix™ (JTP Peptide Technologies GmbH) at 0.1µg/mL per peptide and an irrelevant peptide at 5µg/mL were used as a positive and negative control,

respectively. IFN-γ ELISpot was performed according to the manufacturer's instructions (BD Biosciences). SFC were counted using the AID ELISpot plate reader system ELR04 and software (AID Autoimmun Diagnostika GmbH). Prior depletion of CD8$^+$ T cells used the CD8 T-cell isolation kit (Miltenyi Biotec GmbH) in accordance with the manufacturer's instructions; CD8 T-cell depletion was examined as greater than 85% by flow cytometry. Blocking of MHCII used anti-IA/IE antibody (clone N22, a kind gift from the Antibody and Vaccine Group, Cancer Sciences, University of Southampton, Southampton, UK) at 10ng/µL per well.[2] Following depletion/blocking, IFN-γ ELISpot conditions were as described above.

[1]Pascolo S, Bervas N, Ure J M, et al. HLA-A2.1-restricted education and cytolytic activity of CD8(+) T lymphocytes from beta2 microglobulin (beta2m) HLA-A2.1 monochain transgenic H-2Db beta2m double knockout mice. *J Exp Med* 1997;185(12):2043-51. doi: 10.1084/jem.185.12.2043

[2]Di Genova G, Savelyeva N, Suchacki A, et al. Bystander stimulation of activated CD4+ T cells of unrelated specificity following a booster vaccination with tetanus toxoid. *Eur J Immunol* 2010;40(4):976-85. doi: 10.1002/eji.200940017 [published Online First: 2010/01/28]

SUPPLEMENTARY FIGURES

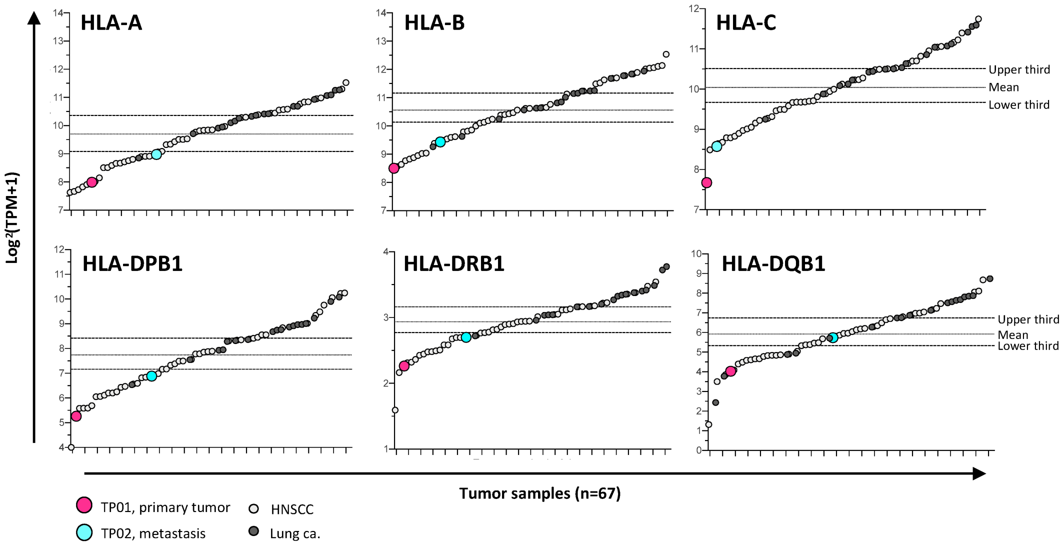

Supplementary Fig. S1. Transcriptomic expression of MHC Class I and II genes in tumour tissue. Assessment of MHC Class I and II gene transcripts (Log2 normalised TPM) in bulk tumour RNA-seq reveals a low level of expression of these genes for both the primary and metastatic sites, which is confined, for the most part, to the lowest tertile. Immune gene contextualisation used a cohort of HNSCC (n=47) and lung cancer (n=18); further details can be found in the "Materials & Methods".

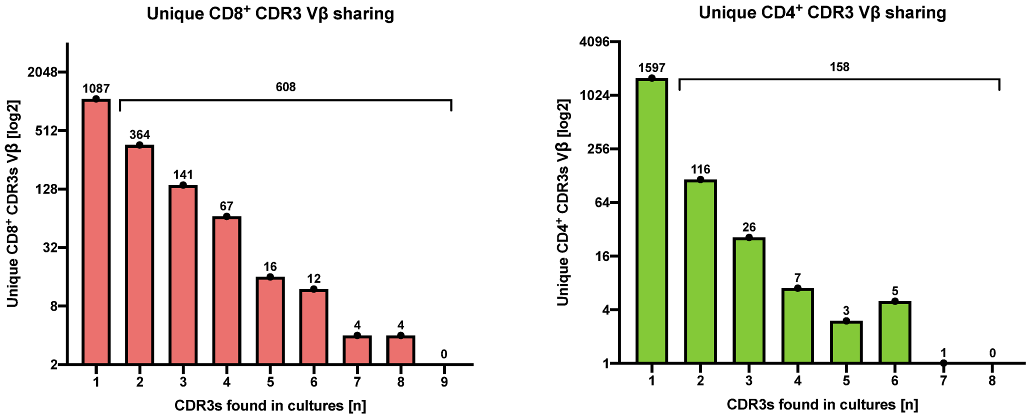

Supplementary Fig. S2. CD8⁺ and CD4⁺ TCR clonotype sharing between PBMC expanded with different neoantigen-specific peptides. Bar graphs to display the degree of sharing of unique $CD8^+$ (red) and $CD4^+$ (green) CDR3 Vβ sequences across cultures expanded with different neoantigen-specific peptides (CD8: 9 cultures, CD4: 8 cultures). The majority of CD8 and CD4 TCR clonotypes are specific to a single neoantigen peptide-expansion: 1087 of 1695 (64%) and 1597 of 1755 (91%) for $CD8^+$ and $CD4^+$, CDR3s respectively.

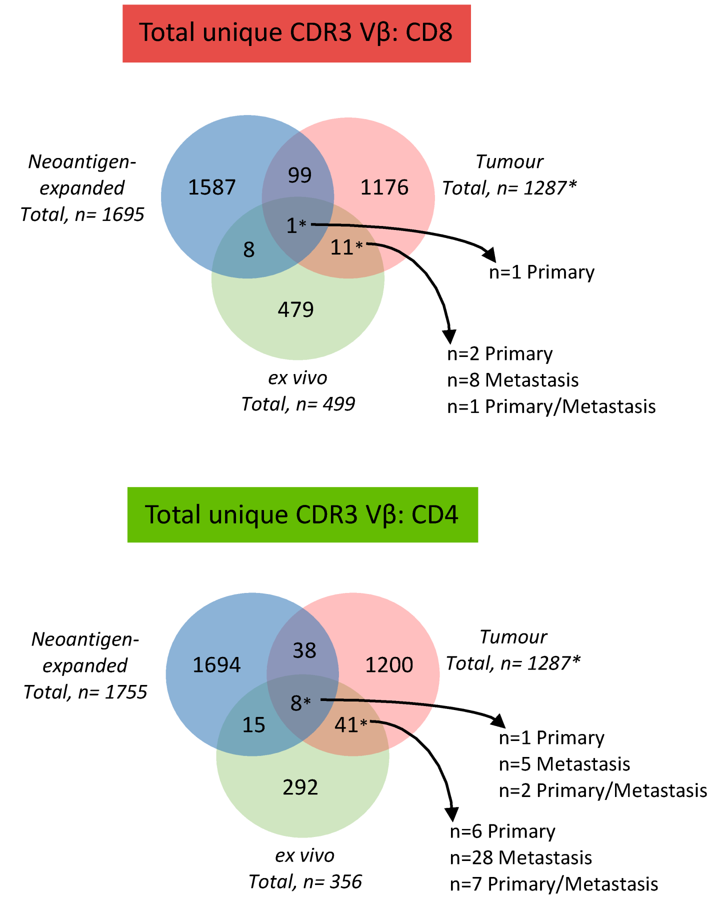

Supplementary Fig. S3. CD8$^+$ and CD4$^+$ TCR clonotype sharing between blood and tumour compartments. Schematic to illustrate the sharing of CD8$^+$ and CD4$^+$ TCR clonotypes recovered from tumour tissue* (primary tumour and metastasis) and the peripheral blood, both from unstimulated PBMC *ex vivo* and PBMC after stimulation with neoantigen-specific peptides. TCR clonotype sharing is evident across all compartments for both CD8$^+$ and CD4$^+$ CDR3 Vβ sequences. *TCR-seq was performed on bulk RNA.

McCann K, *et al. J Immunother Cancer* 2022; 10:e003821. doi: 10.1136/jitc-2021-003821

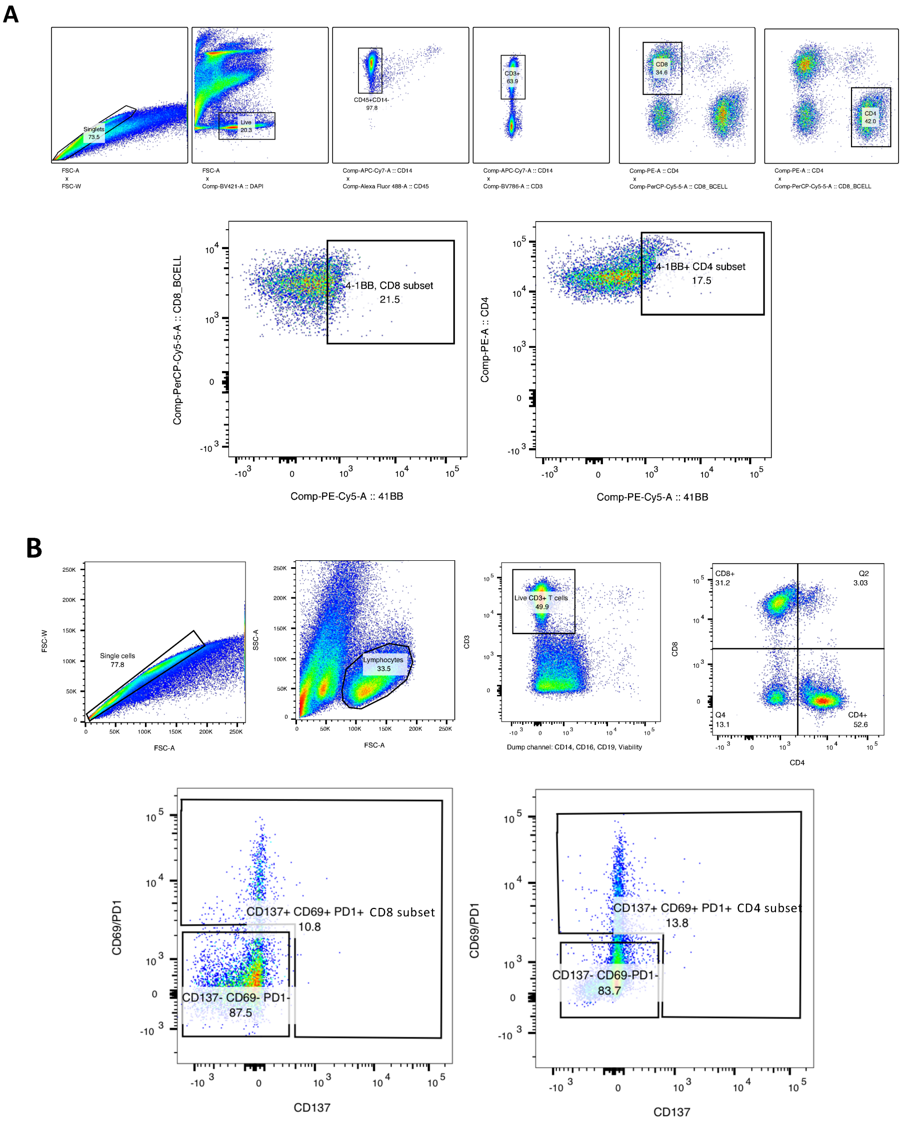

Supplementary Fig. S4. Gating strategy and FACS sorting of CD8⁺ and CD4⁺ T cells with an activated phenotype. Gating strategy used for the sorting of T cells with an activated phenotype for subsequent TCR-seq. **(A)** As defined by CD137 expression, CD137⁺ T cells (sample 1 and 2) and **(B)** As defined by the expression of one or more of the activation markers CD137, CD69 and PD1, CD137⁺ and/or CD69/PD1⁺ T cells (sample 3 and 4).

McCann K, *et al. J Immunother Cancer* 2022; 10:e003821. doi: 10.1136/jitc-2021-003821

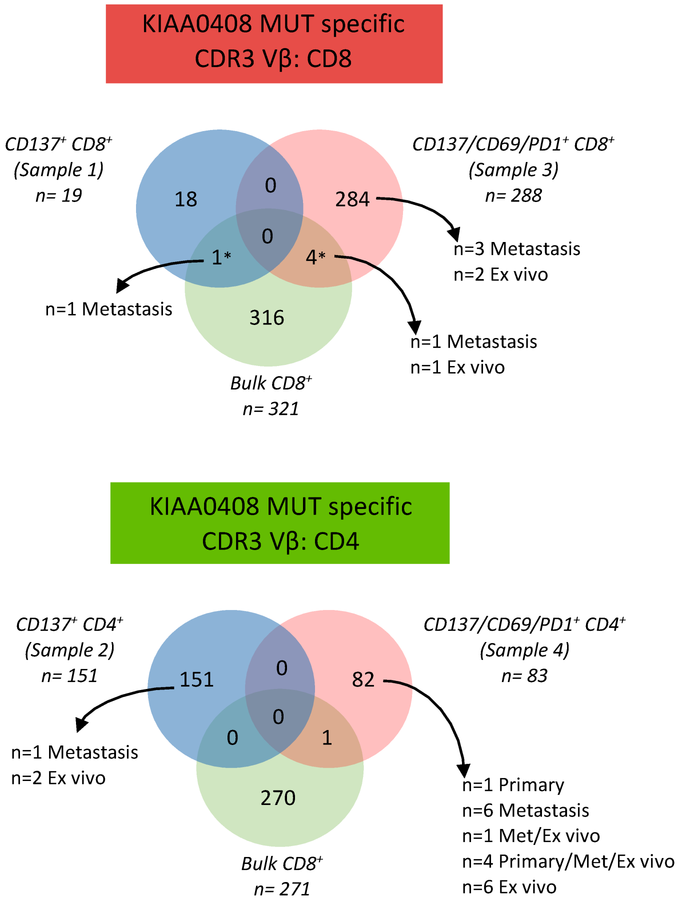

KIAA0408 MUT CDR3 Vβ: CD8		No. sorted cells	Total unique CDR3 Vβ recovered	No. neoantigen-specific CDR3 Vβ	No. shared with previous analysis of bulk CD8+ T cells	No. expanded	Obs. Primary	Obs. Metastasis	Obs. Primary/Met	Obs. Ex vivo
Sample 1	CD8+CD137+	917	31	19 (61%)	1* (5%)	1	0	1	0	0
Sample 3	CD8+CD137+ and/or CD69/PD1+	1250	296	288 (97%)	4*^ (1%)	15	0	4	0	3

KIAA0408 MUT CDR3 Vβ: CD4		No. sorted cells	Total unique CDR3 Vβ recovered	No. neoantigen-specific CDR3 Vβ	No. shared with previous analysis of bulk CD4+ T cells	No. expanded	Obs. Primary	Obs. Metastasis	Obs. Primary/Met	Obs. Ex vivo
Sample 2	CD4+CD137+	442	166	151 (91%)	0 (0%)	4	0	1	0	2
Sample 4	CD4+CD137+ and/or CD69/PD1+	2354	103	83 (81%)	1 (1%)	12	5^	11^	4^	11

*includes TCR clonotypes observed in metastasis
^includes TCR clontypes observed ex vivo

Supplementary Fig. S5. Neoantigen-specific TCR clonotype sharing between activated and bulk CD8+ and CD4+ T cell subsets for KIAA0408 MUT peptide. A schematic to illustrate the sharing of KIAA0408 MUT-specific TCR clonotypes recovered from activated CD8+ and CD4+ T cell subsets with that of bulk CD8+ and CD4+ T cells recovered from the first analysis; sharing with tumor tissue (primary and metastasis) and the peripheral blood directly *ex vivo* is also indicated.

McCann K, *et al. J Immunother Cancer* 2022; 10:e003821. doi: 10.1136/jitc-2021-003821

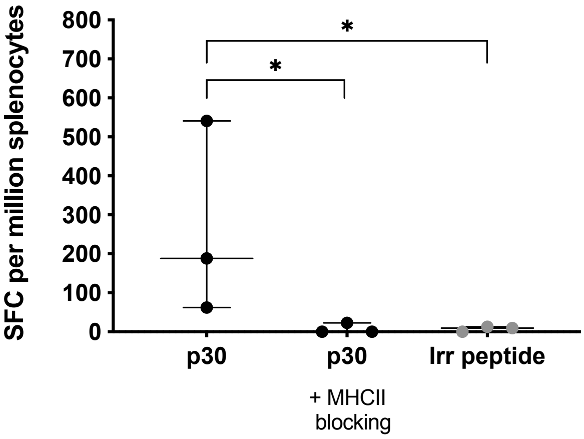

Supplementary Fig. S6. Blocking of MHC class II with anti-IA/IE antibody. MHCII blocking with anti-IA/IE antibody was demonstrated in parallel using a well-defined epitope from tetanus toxin, p30. Mice were vaccinated intramuscularly with 50µg of DNA vaccine encoding domain (DOM) of Fragment C of tetanus toxin; splenocytes were harvested used in an *ex vivo* IFN-γ ELISpot. Two-way ANOVA analysis was performed; significant *P* values: **P*=0.0172 (+MHCII blocking) and **P*=0.0172 (irrelevant peptide). Data are shown for n=3 mice per group.

SUPPLEMENTARY TABLES

Supplementary Table S1. Long peptides used for immunogenicity testing

Gene Symbol	Peptide ID	MUT Long Peptide Sequence	Mutation position	WT Long Peptide Sequence
COG2	COG2-1	EIAGS**S**EAALTDVLEDAPAE	6	NA
	COG2-26	PVYFQIRFREIAGS**S**EAALT	15	
HIPK3	HIPK3-2	VKKLK**A**EPSSCVFQERNYPR	6	NA
	HIPK3-27	QTQSSAFCSVKKLK**A**EPSSC	15	
ARFGAP2	ARFGAP2-3	MAAE**R**NKTEIQTLFKRLRAV	5	NA
POC1B	POC1B-4	FKPHA**K**AYRYVGHKDVVTSV	6	FKPHA**R**AYRYVGHKDVVTSV
	POC1B-28	MLWNFKPHA**K**AYRYVGHKDV	10	MLWNFKPHA**R**AYRYVGHKDV
	POC1B-29	WDTFLMLWNFKPHA**K**AYRYV	15	WDTFLMLWNFKPHA**R**AYRYV
NOL3	NOL3-5	NAQER**L**SETIDRERKRLVET	6	NA
	NOL3-30	GWDRAPTMGNAQER**L**SETID	15	
	NOL3-31	MGNAQER**L**SETIDRERKRLV	8	
MED1	MED1-6	LPPEK**Q**KHQTEDDFQRELFS	6	NA
	MED1-32	KTKKKKSSRLPPEK**Q**KHQTE	15	
ADAT3	ADAT3-7	DGLPY**V**CTGYDLYVTREPCA	6	NA
	ADAT3-33	AVRKLDADEDGLPY**V**CTGYD	15	
KEAP1	KEAP1-8	VGVAV**PWSPAGSRLTSRTVP**	6-	-
	KEAP1-34	TRMTSGRSGVGVAV**PWSPAG**	15-	TRMTSGRSGVGVAV**TMEPCR**
	KEAP1-20	**VPVEALLFLGQKYSPMGSII**	all	-
	KEAP1-21	**GQKYSPMGSIIVFVQKPGLK**	all	-
	KEAP1-22	**EKTALQITHLPGREARMPQC**	all	-
	KEAP1-23	**LKEKTALQITHLPGREARMP**	all	-
	KEAP1-24	**IIVFVQKPGLKEKTALQITH**	all	-
	KEAP1-25	**AGSRLTSRTVPVEALLFLGQ**	all	-
ZNF429	ZNF429-9	EMVDE**T**PDGVSLLLPRLECS	6	NA
	ZNF429-10	EMVDE**T**PVVCSHFAEDFWPE	6	
	ZNF429-35	KEPCKMKRHEMVDE**T**PDGVS	15	
	ZNF429-36	KEPCKMKRHEMVDE**T**PVVCS	15	
PIH1D1	PIH1D1-11	NPEWR**I**MKNRPFMGSISQQN	6	NA
	PIH1D1-37	LEDKYNLQLNPEWR**I**MKNRP	15	
PTOV1	PTOV1-12	PIGPS**L**PGLTLGGLAVSEHR	6	PIGPS**S**PGLTLGGLAVSEHR
	PTOV1-38	GARVFGALGPIGPS**L**PGLTL	15	GARVFGALGPIGPS**S**PGLTL
NIF3L1	NIF3L1-13	ERLVI**L**ALENRVGIYSPHTA	6	ERLVI**R**ALENRVGIYSPHTA
	NIF3L1-39	KRITWNTWKERLVI**L**ALENR	15	KRITWNTWKERLVI**R**ALENR
MAFF	MAFF-14	ALMGL**L**VRELNRHLRGLSAE	6	ALMGL**S**VRELNRHLRGLSAE
	MAFF-15	MGL**L**VRELNRHLRGLSAEEV	4	MGL**S**VRELNRHLRGLSAEEV
	MAFF-40	ENTPHLSDEALMGL**L**VRELN	15	ENTPHLSDEALMGL**S**VRELN
FBLN1	FBLN1-16	CEYSL**I**VGYQCGQVFQACCV	6	NA
	FBLN1-41	RAAQAQGQSCEYSL**I**VGYQC	15	
NAALADL2	NAALADL2-17	QYLDN**N**DLQATALDLEWDME	6	NA
	NAALADL2-42	ADQRAPGHSQYLDN**N**DLQAT	15	

KIAA0408	KIAA0408-46<br>KIAA0408-43	ALRRTTHNYTISL**Q**SEALMV<br>PALRRTTHNYTISL**Q**SEALM	14<br>15	ALRRTTHNYTISL**R**SEALMV<br>PALRRTTHNYTISL**R**SEALM
MAMDC4	MAMDC4-18<br>MAMDC4-44	GTTDF**Q**SPEAGGWEDASVGR<br>AGGEDEQACGTTDF**Q**SPEAG	6<br>15	NA
PHF8	PHF8-19<br>PHF8-45	CVGVE**Q**EKAADIDLYHCPNC<br>MCQDWFHGSCVGVE**Q**EKAAD	6<br>15	CVGVE**E**EKAADIDLYHCPNC<br>MCQDWFHGSCVGVE**E**EKAAD

Supplementary Table S2. List of observed non-synonymous mutations for the primary tumour and metastasis –see pdf

Supplementary Table S3. Patient's HLA Genotype

Loci Name		
HLA A	A*02:01:01:01	A*32:01:01:01
HLA B	B*15:01:01	B*27:05:02
HLA C	C*02:02:02	C*03:03:01
HLA DRB1	DRB1*12:01:01	DRB1*13:01:01
HLA DQB1	DQB1*03:01:01	DQB1*06:03:01
HLA DPB1	DPB1*03:01:01G	DPB1*04:01:01G

Supplementary Table S4. Summary of $CD8^+$ and $CD4^+$ TCR clonotype sharing between MUT and WT

		Total unique CDR3 Vβ recovered (MUT)	Total unique CDR3 Vβ recovered (WT)	No. shared by MUT and WT	No. shared that are virus-reactive
**CD8**	MAFF	323	425	174 (23%)	4 (2%)
	POC1B	121	96	24 (11%)	1 (4%)
	NIF3L1	276	365	111 (17%)	3 (3%)
	KEAP1	443	317	181 (23%)	3 (2%)
	KIAA0408	444	NA	NA	NA
**CD4**	MAFF	241	251	19 (4%)	0 (0%)
	POC1B	111	173	1 (<1%)	0 (0%)
	NIF3L1	268	361	18 (3%)	0 (0%)
	KEAP1	293	NA	NA	NA
	KIAA0408	315	NA	NA	NA

Supplementary Table S5. List of KIAA0408 MUT-specific CD8⁺ and CD4⁺ TCR clonotypes –see pdf

Supplementary Table S6. List of neoantigen-specific CD8⁺ and CD4⁺ TCR clonotypes shared with tumour tissue –see pdf

Supplementary Table S7 MHC Class I epitope prediction

Gene Symbol	HLA allele	Specificity	Predicted MHC Class I epitope	Length	Predictive Score[a]	Corresponding Peptide ID
POC1B	HLA-A*02:01	MUT/WT	MLWNFKPHA	9	11.64	POC1B-28, -29
	HLA-A*02:01	MUT/WT	FLMLWNFKPHA	11	37.17	POCB1-29
	HLA-A*02:01	MUT/WT	FLMLWNFKP	9	88.34	POC1B-29
	HLA-A*02:01	MUT/WT	LMLWNFKPHA	10	92.68	POC1B-29
	HLA-A*02:01	WT	MLWNFKPHAR	10	353.19	POC1B-28, -29
	HLA-A*02:01	MUT	MLWNFKPHAK	10	356.99	POC1B-28, -29
	HLA-A*32:01	MUT/WT	DTFLMLWNF	9	103.42	POC1B-29
	HLA-B*15:01	WT	WNFKPHARAY	10	398.53	POC1B-28, -29
	HLA-B*15:01	MUT	WNFKPHAKAY	10	459.38	POC1B-28, -29
	HLA-B*27:05	WT	ARAYRYVGHK	10	80.12	POC1B-4, -28
KEAP1	HLA-A*02:01	MUT	**RLTSRTVPV**	9	23.30	KEAP1-25
	HLA-A*02:01	MUT	**SPMGSIIVFV**	10	33.57	KEAP1-21
	HLA-A*02:01	MUT	**YSPMGSIIVFV**	11	36.09	KEAP-21
	HLA-A*02:01	MUT	**FLGQKYSPM**	9	145.50	KEAP1-20
	HLA-A*02:01	MUT	**SRLTSRTVPV**	10	151.33	KEAP1-25
	HLA-A*02:01	MUT	**PMGSIIVFV**	9	285.07	KEAP1-21
	HLA-A*02:01	MUT	**MGSIIVFV**	8	286.59	KEAP1-21
	HLA-A*32:01	MUT	**RLTSRTVPV**	9	123.10	KEAP1-25
	HLA-A*32:01	MUT	RSGVGVAV**PW**	10	452.18	KEAP1-34
	HLA-B*15:01	MUT	**YSPMGSIIVF**	10	9.55	KEAP1-21
	HLA-B*15:01	MUT	**GQKYSPMGSI**	10	215.88	KEAP1-20, -21
	HLA-B*15:01	MUT	**FLGQKYSPM**	9	216.82	KEAP1-20
	HLA-B*15:01	MUT	**LFLGQKYSPM**	10	260.96	KEAP1-20
	HLA-B*15:01	MUT	**RTVPVEALLF**	10	279.22	KEAP1-25
	HLA-B*15:01	WT	RSGVGVAVTM	10	282.26	KEAP1-34
	HLA-B*27:05	MUT	**SRLTSRTVPV**	10	257.66	KEAP1-25
PTOV1	HLA-A*02:01	MUT	ALGPIGPSL	9	120.20	PTOV1-38
	HLA-A*02:01	MUT	SLPGLTLGGL	10	364.88	PTOV1-12
	HLA-A*02:01	MUT/WT	RVFGALGPI	9	459.41	PTOV1-38
	HLA-A*32:01	MUT/WT	RVFGALGPI	9	13.01	PTOV1-38
	HLA-B*15:01	MUT/WT	RVFGALGPI	9	498.08	PTOV1-38
	HLA-B*27:05	MUT/WT	ARVFGALGPI	10	72.99	PTOV1-38
NIF3L1	HLA-A*02:01	MUT	VILALENRV	9	382.35	NIF3L1-13
	HLA-A*32:01	WT	RALENRVGI	9	227.12	NIF3L1-13
	HLA-B*27:05	MUT/WT	KRITWNTWK	9	45.20	NIF3L1-39
	HLA-B*27:05	MUT/WT	KRITWNTWKE	10	92.72	NIF3L1-39
	HLA-B*27:05	WT	IRALENRVGI	10	188.43	NIF3L1-13
	HLA-B*27:05	MUT/WT	KRITWNTWKE	11	374.67	NIF3L1-39
MAFF	HLA-A*02:01	MUT	ALMGLLVREL	10	14.04	MAFF-14, -40
	HLA-A*02:01	WT	ALMGLSVREL	10	26.94	MAFF-14, -40
	HLA-A*02:01	MUT	EALMGLLVREL	11	98.38	MAFF-40
	HLA-A*02:01	MUT/WT	HLSDEALMGL	10	128.35	MAFF-40
	HLA-A*02:01	WT	EALMGLSVREL	11	269.92	MAFF-40
	HLA-A*02:01	MUT	ALMGLLVRELN	11	315.27	MAFF-14, -40

	HLA-B*27:05	MUT/WT	LNRHLRGLSA	10	296.44	MAFF-14, -15
	HLA-B*27:05	MUT/WT	NRHLRGLSA	9	470.15	MAFF-14, -15
	HLA-A*02:01	MUT	SL**Q**SEALMV	9	81.58	KIAA0408-46
	HLA-A*02:01	MUT	ISL**Q**SEALMV	10	409.57	KIAA0408-46
	HLA-A*32:01	MUT/WT	TTHNYTISL	9	59.77	KIAA0408-43, -46
	HLA-A*32:01	MUT/WT	RTTHNYTISL	10	136.33	KIAA0408-43, -46
	HLA-A*32:01	MUT/WT	RTTHNYTI	8	223.51	KIAA0408-43, -46
	HLA-A*32:01	MUT/WT	RTTHNYTISL	9	473.45	KIAA0408-43, -46
KIAA0408	HLA-B*15:01	MUT/WT	ALRRTTHNY	9	87.89	KIAA0408-43, -46
	HLA-B*15:01	MUT	YTISL**Q**SEAL	10	268.63	KIAA0408-43, -46
	HLA-B*15:01	WT	YTISLRSEAL	10	276.68	KIAA0408-43, -46
	HLA-B*27:05	MUT/WT	RRTTHNYTI	9	59.78	KIAA0408-43, -46
	HLA-B*27:05	MUT/WT	RRTTHNYTISL	11	60.73	KIAA0408-43, -46
	HLA-B*27:05	MUT/WT	RRTTHNYTIS	9	108.59	KIAA0408-43, -46
	HLA-B*27:05	MUT/WT	LRRTTHNYTI	10	323.46	KIAA0408-43, -46
PHF8	-	-	-	-	-	-

[a];Predicted epitopes with an IC_{50} value ≤500nM (IEDB NetMHCpan BA 4.0) are displayed.
