# Supplementary material for: Targeting the tumor mutanome for personalized vaccination in a TMB low non-small cell lung cancer
Source: J Immunother Cancer. 2022 Mar 30;10(3):e003821. doi: 10.1136/jitc-2021-003821 (PMC8971766; doi:10.1136/jitc-2021-003821)
Supplement: Supplementary data [file jitc-2021-003821supp002.pdf]

Primary tumour (TP01)
Non-synonymous mutations, n= 102

	Variant_ID	Variant_Type	Chromosome	Position	Ref_Seq	Alt_Seq	dbSNP_ID	Tum_Genotype	Tum_Genotype_Code	Effect	Gene_Name	Gene_ID	Feature_Type	Feature_ID	Transcript_Biotype	hgvs_DNA	hgvs_Protein	Protein_Ref	Protein_Alt	cDNA_Position	cds_Position	Protein_Position
1	906425	snp	chr7	116412043	G	A	.	G/A	'0/1	missense_variant&splice_reg	MET	ENSG00000105976	transcript	ENST00000397752	protein_coding	c.3028G>A	p.Asp1010Asn	Asp	Asn	'3228/6635	'3028/4173	'1010/1390
2	914596	snp	chr7	142723874	C	T	.	C/T	'0/1	missense_variant	OR9A2	ENSG00000179468	transcript	ENST00000350513	protein_coding	c.346G>A	p.Val116Met	Val	Met	'409/1038	'346/933	'116/310
3	901237	snp	chr7	100373361	T	A	.	T/A	'0/1	missense_variant	ZAN	ENSG00000146839	transcript	ENST00000546213	protein_coding	c.1628T>A	p.Ile543Asn	Ile	Asn	'6045/8036	'1628/3555	'543/1184
4	248042	snp	chr13	41373234	A	T	.	A/T	'0/1	missense_variant	SLC25A15	ENSG00000102743	transcript	ENST00000417731	protein_coding	c.97A>T	p.Met33Leu	Met	Leu	'166/468	'97/399	'33/132
5	482538	snp	chr19	21713480	C	A	rs532407132	C/A	'0/1	missense_variant	ZNF429	ENSG00000197013	transcript	ENST00000597078	protein_coding	c.220C>A	p.Pro74Thr	Pro	Thr	'388/1043	'220/369	'74/122
6	149259	snp	chr11	33308043	T	C	.	T/C	'0/1	missense_variant	HIPK3	ENSG00000110422	transcript	ENST00000456517	protein_coding	c.83T>C	p.Val28Ala	Val	Ala	'445/4478	'83/3585	'28/1194
7	959535	snp	chr8	143998537	C	T	.	C/T	'0/1	missense_variant	CYP11B2	ENSG00000179142	transcript	ENST00000323110	protein_coding	c.333G>A	p.Met111Ile	Met	Ile	'336/2936	'333/1512	'111/503
8	398915	snp	chr17	37566375	G	T	.	G/T	'0/1	missense_variant	MED1	ENSG00000125686	transcript	ENST00000300651	protein_coding	c.2099C>A	p.Pro700Gln	Pro	Gln	'2323/5844	'2099/4746	'700/1581
9	921885	del	chr8	3076899	AG	A	.	AG/A	'0/1	frameshift_variant	CSMD1	ENSG00000183117	transcript	ENST00000539096	protein_coding	c.4549delC	p.Leu1517fs	Leu	fs	'4549/6330	'4549/6330	'1517/2109
10	572009	snp	chr2	201757010	G	T	.	G/T	'0/1	missense_variant	NIF3L1	ENSG00000196290	transcript	ENST00000409588	protein_coding	c.344G>T	p.Arg115Leu	Arg	Leu	'371/1269	'344/858	'115/285
11	82441	snp	chr1	230819343	T	C	.	T/C	'0/1	missense_variant	COG2	ENSG00000135775	transcript	ENST00000546013	protein_coding	c.257T>C	p.Leu86Ser	Leu	Ser	'312/1400	'257/1284	'86/427
12	464252	del	chr19	10597378	TG	T	.	TG/T	'0/1	frameshift_variant	KEAP1	ENSG00000079999	transcript	ENST00000393623	protein_coding	c.1824delC	p.Val608fs	Val	fs	'2071/2648	'1824/1875	'608/624
13	152937	snp	chr11	47198391	G	C	.	G/C	'0/1	missense_variant	ARFGAP2	ENSG00000149182	transcript	ENST00000530596	protein_coding	c.14C>G	p.Pro5Arg	Pro	Arg	'29/534	'14/519	'5/172
14	764980	snp	chr5	5463702	G	A	.	G/A	'0/1	missense_variant	KIAA0947	ENSG00000164151	transcript	ENST00000296564	protein_coding	c.4255G>A	p.Glu1419Lys	Glu	Lys	'4477/7927	'4255/6801	'1419/2266
15	364840	snp	chr16	67208095	C	T	.	C/T	'0/1	missense_variant	NOL3	ENSG00000140939	transcript	ENST00000568146	protein_coding	c.23C>T	p.Pro8Leu	Pro	Leu	'76/1351	'23/660	'8/219
16	1042108	snp	chrX	100608329	C	T	.	C/T	'0/1	missense_variant	BTK	ENSG00000010671	transcript	ENST00000372880	protein_coding	c.1233G>A	p.Met411Ile	Met	Ile	'1546/2203	'1233/1452	'411/483
17	647609	snp	chr22	38610488	C	T	.	C/T	'0/1	missense_variant	MAFF	ENSG00000185022	transcript	ENST00000407965	protein_coding	c.98C>T	p.Ser33Leu	Ser	Leu	'301/2325	'98/495	'33/164
18	1036381	snp	chrX	54049260	C	G	.	C/G	'0/1	missense_variant	PHF8	ENSG00000172943	transcript	ENST00000445025	protein_coding	c.115G>C	p.Glu39Gln	Glu	Gln	'297/475	'115/293	'39/96
19	505745	snp	chr19	49950714	C	T	.	C/T	'0/1	missense_variant	PIH1D1	ENSG00000104872	transcript	ENST00000597415	protein_coding	c.459G>A	p.Met153Ile	Met	Ile	'841/874	'459/492	'153/163
20	710561	snp	chr3	195474176	G	T	.	G/T	'0/1	missense_variant	MUC4	ENSG00000145113	transcript	ENST00000475231	protein_coding	c.15954C>A	p.Phe5318Leu	Phe	Leu	'16026/16273	'15954/16083	'5318/5360
21	848795	snp	chr6	127765276	C	T	.	C/T	'0/1	missense_variant	KIAA0408	ENSG00000189367	transcript	ENST00000483725	protein_coding	c.2063G>A	p.Arg688Gln	Arg	Gln	'2400/3284	'2063/2085	'688/694
22	969228	snp	chr9	33798574	G	A	rs201773718	G/A	'0/1	missense_variant	PRSS3	ENSG00000010438	transcript	ENST00000379405	protein_coding	c.545G>A	p.Ser182Asn	Ser	Asn	'584/834	'545/744	'182/247
23	40953	snp	chr1	94522308	G	C	.	G/C	'0/1	missense_variant	ABCA4	ENSG00000198691	transcript	ENST00000370225	protein_coding	c.2231C>G	p.Thr744Ser	Thr	Ser	'2318/7309	'2231/6822	'744/2273
24	390420	snp	chr17	21319079	C	A	rs76518282	C/A	'0/1	missense_variant	KCNJ12	ENSG00000184185	transcript	ENST00000583088	protein_coding	c.425C>A	p.Thr142Asn	Thr	Asn	'1320/5420	'425/1302	'142/433
25	221447	snp	chr12	89891065	C	T	.	C/T	'0/1	missense_variant	POC1B	ENSG00000139323	transcript	ENST00000549035	protein_coding	c.29G>A	p.Arg10Lys	Arg	Lys	'499/2038	'29/1311	'10/436
26	40008	snp	chr1	91844029	C	A	.	C/A	'0/1	missense_variant	HFM1	ENSG00000162669	transcript	ENST00000370424	protein_coding	c.204G>T	p.Trp68Cys	Trp	Cys	'434/3675	'204/3345	'68/1114
27	793368	snp	chr5	132158781	C	G	.	C/G	'0/1	missense_variant	SHROOM1	ENSG00000164403	transcript	ENST00000378676	protein_coding	c.2059G>C	p.Glu687Gln	Glu	Gln	'2059/2352	'2059/2352	'687/783
28	898445	ins	chr7	96635420	A	AGCC	rs559903070	AGCC/AGCC	'1/1	disruptive_inframe_insertion	DLX6	ENSG00000006377	transcript	ENST00000518156	protein_coding	c.158_160dupCGC	p.Pro53dup	Pro	dup	'591/2304	'161/882	'54/293
29	653926	snp	chr22	49246651	G	A	.	G/A	'0/1	missense_variant	FAM19A5	ENSG00000219438	transcript	ENST00000336769	protein_coding	c.472G>A	p.Asp158Asn	Asp	Asn	'473/546	'472/545	'158/180
30	1004854	snp	chr9	139750198	G	C	.	G/C	'0/1	missense_variant	MAMDC4	ENSG00000177943	transcript	ENST00000317446	protein_coding	c.1486G>C	p.Glu496Gln	Glu	Gln	'1536/3658	'1486/3414	'496/1137
31	203710	snp	chr12	48880492	G	A	.	G/A	'0/1	missense_variant	C12orf54	ENSG00000177627	transcript	ENST00000548364	protein_coding	c.118G>A	p.Glu40Lys	Glu	Lys	'175/687	'118/384	'40/127
32	604285	snp	chr20	43057062	A	G	.	A/G	'0/1	missense_variant	HNF4A	ENSG00000101076	transcript	ENST00000415691	protein_coding	c.1217A>G	p.Asn406Ser	Asn	Ser	'1289/2302	'1217/1395	'406/464
33	933507	snp	chr8	39009041	G	T	.	G/T	'0/1	missense_variant	ADAM32	ENSG00000197140	transcript	ENST00000522506	protein_coding	c.499G>T	p.Asp167Tyr	Asp	Tyr	'502/557	'499/554	'167/183
34	448386	snp	chr19	1912926	C	G	.	C/G	'0/1	missense_variant	ADAT3	ENSG00000213638	transcript	ENST00000602400	protein_coding	c.832C>G	p.Leu278Val	Leu	Val	'1060/1578	'832/1056	'278/351
35	387446	snp	chr17	16843707	C	G	.	C/G	'0/1	missense_variant	TNFRSF13B	ENSG00000240505	transcript	ENST00000583789	protein_coding	c.426G>C	p.Lys142Asn	Lys	Asn	'438/859	'426/744	'142/247
36	652346	snp	chr22	45923798	G	T	.	G/T	'0/1	missense_variant	FBLN1	ENSG00000077942	transcript	ENST00000451475	protein_coding	c.213G>T	p.Met71Ile	Met	Ile	'213/544	'213/544	'71/180
37	510655	snp	chr19	54745989	G	C	rs1052963	G/C	'0/1	missense_variant	LILRA6	ENSG00000244482	transcript	ENST00000245621	protein_coding	c.268C>G	p.Gln90Glu	Gln	Glu	'268/1395	'268/1395	'90/464
38	796410	snp	chr5	140221195	G	C	rs199713478	G/C	'0/1	missense_variant	PCDHA8	ENSG00000204962	transcript	ENST00000378123	protein_coding	c.289G>C	p.Gly97Arg	Gly	Arg	'289/2445	'289/2445	'97/814
39	97877	snp	chr10	29783908	A	G	rs78773460	A/G	'0/1	missense_variant	SVIL	ENSG00000197321	transcript	ENST00000538146	protein_coding	c.152T>C	p.Met51Thr	Met	Thr	'234/2211	'152/1758	'51/585
40	704363	snp	chr3	174814651	G	A	.	G/A	'0/1	missense_variant	NAALADL2	ENSG00000177694	transcript	ENST00000434257	protein_coding	c.64G>A	p.Asp22Asn	Asp	Asn	'293/574	'64/345	'22/114
41	375132	snp	chr16	88791458	G	A	rs11645197	G/A	'0/1	missense_variant	PIEZO1	ENSG00000103335	transcript	ENST00000301015	protein_coding	c.4193C>T	p.Pro1398Leu	Pro	Leu	'4440/8072	'4193/7566	'1398/2521
42	203961	snp	chr12	49229958	G	A	.	G/A	'0/1	missense_variant	DDX23	ENSG00000174243	transcript	ENST00000308025	protein_coding	c.1328C>T	p.Ala443Val	Ala	Val	'1408/3248	'1328/2463	'443/820
43	137831	snp	chr11	1016609	C	G	rs73403291	C/G	'0/1	missense_variant	MUC6	ENSG00000184956	transcript	ENST00000421673	protein_coding	c.6192G>C	p.Met2064Ile	Met	Ile	'6243/8006	'6192/7320	'2064/2439
44	764901	snp	chr5	5303424	G	A	rs34586473	G/A	'0/1	missense_variant	ADAMTS16	ENSG00000145536	transcript	ENST00000274181	protein_coding	c.2833G>A	p.Gly945Arg	Gly	Arg	'2971/4979	'2833/3675	'945/1224
45	458781	del	chr19	7696610	TC	T	.	TC/T	'0/1	frameshift_variant	PCP2	ENSG00000174788	transcript	ENST00000598935	protein_coding	c.327delG	p.Arg109fs	Arg	fs	'343/457	'327/363	'109/120
46	637449	snp	chr22	22707564	G	C	rs539733792	G/C	'0/1	missense_variant	IGLV5-48	ENSG00000211647	transcript	ENST00000390293	IG_V_gene	c.152G>C	p.Ser51Thr	Ser	Thr	'152/369	'152/369	'51/122
47	914226	snp	chr7	142247546	C	A	rs575564328	C/A	'0/1	missense_variant	TRBV7-3	ENSG00000211714	transcript	ENST00000390361	TR_V_gene	c.20G>T	p.Cys7Phe	Cys	Phe	'70/397	'20/347	'7/114
48	719960	snp	chr4	16597450	C	A	.	C/A	'0/1	missense_variant	LDB2	ENSG00000169744	transcript	ENST00000506732	protein_coding	c.212G>T	p.Gly71Val	Gly	Val	'286/567	'212/493	'71/163
49	506321	snp	chr19	50357709	C	T	.	C/T	'0/1	missense_variant	PTOV1	ENSG00000104960	transcript	ENST00000600793	protein_coding	c.47C>T	p.Ser16Leu	Ser	Leu	'47/426	'47/426	'16/141
50	901752	complex	chr7	100616227	AGG	TGC	.	AGG/TGC	'0/1	missense_variant	MUC12	ENSG00000205277	transcript	ENST00000379442	protein_coding	c.190_192delAGGin	p.Arg64Cys	Arg	Cys	'190/16737	'190/16437	'64/5478
51	914144	snp	chr7	142206770	A	G	.	A/G	'0/1	missense_variant	TRBV10-2	ENSG00000229769	transcript	ENST00000426318	TR_V_gene	c.85T>C	p.Tyr29His	Tyr	His	'136/395	'85/344	'29/113
52	1044457	snp	chrX	118605001	G	T	rs73213195	G/T	'0/1	stop_gained	SLC25A5	ENSG00000005022	transcript	ENST00000317881	protein_coding	c.877G>T	p.Glu293*	Glu	*	'993/1274	'877/897	'293/298
53	6176	del	chr1	8716321	CTCTTTG	C	rs3831914	CTCTTTG/C	'0/1	disruptive_inframe_deletion	RERE	ENSG00000142599	transcript	ENST00000468247	protein_coding	c.30_35delCAAAGA	p.Asp10_Lys11del	Asp	_Lys11del	'344/414	'30/105	'10/34
54	914143	snp	chr7	142206764	T	C	.	T/C	'0/1	missense_variant	TRBV10-2	ENSG00000229769	transcript	ENST00000426318	TR_V_gene	c.91A>G	p.Ile31Val	Ile	Val	'142/395	'91/344	'31/113
55	227413	snp	chr12	109017374	T	G	rs201674915	T/G	'0/1	missense_variant	SELPLG	ENSG00000110876	transcript	ENST00000550948	protein_coding	c.710A>C	p.Glu237Ala	Glu	Ala	'935/2256	'710/1239	'237/412
56	505347	ins	chr19	49657889	T	TTCC	rs57199624	TTCC/TTCC	'1/1	disruptive_inframe_insertion	HRC	ENSG00000130528	transcript	ENST00000595625	protein_coding	c.603_605dupGGA	p.Glu202dup	Glu	dup	'611/2044	'605/2031	'202/676
57	968944	complex	chr9	33386465	AGACC	GGACT	.	AGACC/GGACT	'0/1	missense_variant	AQP7	ENSG00000165269	transcript	ENST00000379503	protein_coding	c.147_151delGGTC	p.ProValTyr49ProVal	ProValTyr	ProValHis	'193/677	'147/582	'49/193
58	1036320	snp	chrX	53652846	G	T	rs74381511	G/T	'0/1	missense_variant	HUWE1	ENSG00000086758	transcript	ENST00000424562	protein_coding	c.424C>A	p.Pro142Thr	Pro	Thr	'425/682	'424/681	'142/226
59	137827	complex	chr11	1016579	GGT	CGC	.	GGT/CGC	'0/1	missense_variant	MUC6	ENSG00000184956	transcript	ENST00000421673	protein_coding	c.6220_6222delACC	p.Thr2074Ala	Thr	Ala	'6273/8006	'6220/7320	'2074/2439
60	10972	snp	chr1	16894493	G	C	rs3932639	G/C	'0/1	missense_variant	NBPF1	ENSG00000219481	transcript	ENST00000430580	protein_coding	c.2647C>G	p.Gln883Glu	Gln	Glu	'3535/5932	'2647/3420	'883/1139
61	10972	snp	chr1	16894493	G	C	rs3932639	G/C	'0/1	missense_variant	NBPF1	ENSG00000219481	transcript	ENST00000432949	protein_coding	c.857C>G	p.Pro286Arg	Pro	Arg	'857/1069	'857/900	'286/299
62	500901	ins	chr19	46265047	A	ATCCAGC	rs59054027	ATCCAGC/ATCCAGC	'1/1	disruptive_inframe_insertion	AC074212.3	ENSG00000237452	transcript	ENST00000457052	protein_coding	c.1389_1394dupCA	p.Ser464_Ser465dup	Ser	_Ser465dup	'1811/2749	'1395/1917	'465/638
63	1044456	mnp	chrX	118604969	TG	CA	.	TG/CA	'0/1	missense_variant	SLC25A5	ENSG00000005022	transcript	ENST00000317881	protein_coding	c.845_846delTGins	p.Met282Thr	Met	Thr	'961/1274	'845/897	'282/298
64	481901	snp	chr19	21366439	G	T	.	G/T	'0/1	missense_variant	ZNF431	ENSG00000196705	transcript	ENST00000311048	protein_coding	c.1333G>T	p.Ala445Ser	Ala	Ser	'1477/3843	'1333/1731	'445/576
65	1044455	snp	chrX	118604954	A	G	rs74778762	A/G	'0/1	missense_variant	SLC25A5	ENSG00000005022	transcript	ENST00000317881	protein_coding	c.830A>G	p.Asn277Ser	Asn	Ser	'946/1274	'830/897	'277/298
66	10983	snp	chr1	16902844	G	T	rs199969012	G/T	'0/1	missense_variant	NBPF1	ENSG00000219481	transcript	ENST00000287968	protein_coding	c.132C>A	p.Asp44Glu	Asp	Glu	'132/694	'132/492	'44/163
67	495641	snp	chr19	41103231	A	C	.	A/C	'0/1	missense_variant	LTBP4	ENSG00000090006	transcript	ENST00000308370	protein_coding	c.91A>C	p.Ser31Arg	Ser	Arg	'91/5142	'91/4872	'31/1623
68	901754	snp	chr7	100616320	G	A	rs200695576	G/A	'0/1	missense_variant	MUC12	ENSG00000205277	transcript	ENST00000379442	protein_coding	c.283G>A	p.Val95Met	Val	Met	'283/16737	'283/16437	'95/5478
69	822841	snp	chr6	32610495	A	G	.	A/G	'0/1	missense_variant	HLA-DQA1	ENSG00000196735	transcript	ENST00000374949	protein_coding	c.722A>G	p.Gln241Arg	Gln	Arg	'761/1060	'722/768	'241/255
70	914145	mnp	chr7	142206799	CT	AC	.	CT/AC	'0/1	missense_variant	TRBV10-2	ENSG00000229769	transcript	ENST00000426318	TR_V_gene	c.55_56delAGinsGT	p.Arg19Val	Arg	Val	'107/395	'55/344	'19/113
71	249389	snp	chr13	46170839	G	T	.	G/T	'0/1	missense_variant	FAM194B	ENSG00000165837	transcript	ENST00000298738	protein_coding	c.302C>A	p.Ala101Glu	Ala	Glu	'467/2421	'302/2091	'101/696
72	554816	snp	chr2	130951754	G	T	rs13000249	G/T	'0/1	missense_variant	TUBA3E	ENSG00000152086	transcript	ENST00000312988	protein_coding	c.661C>A	p.Arg221Ser	Arg	Ser	'762/1540	'661/1353	'221/450
73	901753	complex	chr7	100616310	GAGT	CAGA	.	GAGT/CAGA	'0/1	missense_variant	MUC12	ENSG00000205277	transcript	ENST00000379442	protein_coding	c.273_276delGAGTi	p.GluSer91AspArg	GluSer	AspArg	'273/16737	'273/16437	'91/5478
74	135564	snp	chr10	135012492	A	C	.	A/C	'0/1	missense_variant	KNDC1	ENSG00000171798	transcript	ENST00000368571	protein_coding	c.2285A>C	p.His762Pro	His	Pro	'2575/3925	'2285/3426	'762/1141
75	55359	snp	chr1	152188847	A	G	rs145667921	A/G	'0/1	missense_variant	HRNR	ENSG00000197915	transcript	ENST00000368801	protein_coding	c.5258T>C	p.Val1753Ala	Val	Ala	'5334/9623	'5258/8553	'1753/2850
76	243861	complex	chr13	25670776	CACAC	TGCAG	.	CACAC/TGCAG	'0/1	missense_variant	PABPC3	ENSG00000151846	transcript	ENST00000281589	protein_coding	c.440_444delCACA	p.ThrHis147MetGln	ThrHis	MetGln	'477/3090	'440/1896	'147/631
77	206849	snp	chr12	53069233	C	A	.	C/A	'0/1	missense_variant	KRT1	ENSG00000167768	transcript	ENST00000252244	protein_coding	c.1679G>T	p.Gly560Val	Gly	Val	'1738/2451	'1679/1935	'560/644
78	299070	snp	chr15	23685647	C	T	rs368864824	C/T	'0/1	missense_variant	GOLGA6L2	ENSG00000174450	transcript	ENST00000567107	protein_coding	c.1975G>A	p.Glu659Lys	Glu	Lys	'2028/3030	'1975/2730	'659/909
79	544610	snp	chr2	96557455	T	A	.	T/A	'0/1	missense_variant	ANKRD36C	ENSG00000174501	transcript	ENST00000295246	protein_coding	c.1078A>T	p.Asn360Tyr	Asn	Tyr	'1820/2560	'1078/1818	'360/605
80	901750	snp	chr7	100616140	G	T	rs78186722	G/T	'0/1	missense_variant&splice_reg	MUC12	ENSG00000205277	transcript	ENST00000379442	protein_coding	c.103G>T	p.Gly35Cys	Gly	Cys	'103/16737	'103/16437	'35/5478
81	298798	del	chr15	23086364	GGCC	G	rs531550505	GGCC/G	'0/1	disruptive_inframe_deletion	NIPA1	ENSG00000170113	transcript	ENST00000337435	protein_coding	c.45_47delGGC	p.Ala16del	Ala	del	'72/6567	'45/990	'15/329
82	901756	snp	chr7	100616344	G	A	.	G/A	'0/1	missense_variant	MUC12	ENSG00000205277	transcript	ENST00000379442	protein_coding	c.307G>A	p.Gly103Arg	Gly	Arg	'307/16737	'307/16437	'103/5478
83	901755	complex	chr7	100616332	TTGTGGA	CTGTAGG	.	TTGTGGA/CTGTAGG	'0/1	stop_gained	MUC12	ENSG00000205277	transcript	ENST00000379442	protein_coding	c.295_301delTTGTG	p.LeuTrpMet99*	LeuTrpMet	*	'295/16737	'295/16437	'99/5478
84	901751	complex	chr7	100616146	TTGTGG	CTGAGA	.	TTGTGG/CTGAGA	'0/1	missense_variant	MUC12	ENSG00000205277	transcript	ENST00000379442	protein_coding	c.109_114delTTGTG	p.LeuTrp37LeuArg	LeuTrp	LeuArg	'109/16737	'109/16437	'37/5478
85	400199	snp	chr17	39254133	G	T	rs200462175	G/T	'0/1	missense_variant	KRTAP4-8	ENSG00000204880	transcript	ENST00000333822	protein_coding	c.204C>A	p.Ser68Arg	Ser	Arg	'261/1161	'204/558	'68/185
86	368681	snp	chr16	74425769	C	T	rs200486331	C/T	'0/1	missense_variant	NPIPB15	ENSG00000196436	transcript	ENST00000429990	protein_coding	c.1123C>T	p.Arg375Trp	Arg	Trp	'1219/1428	'1123/1332	'375/443
87	710921	snp	chr3	195512117	C	A	.	C/A	'0/1	missense_variant	MUC4	ENSG00000145113	transcript	ENST00000475231	protein_coding	c.6334G>T	p.Ala2112Ser	Ala	Ser	'6406/16273	'6334/16083	'2112/5360
88	368687	snp	chr16	74425826	G	A	.	G/A	'0/1	missense_variant	NPIPB15	ENSG00000196436	transcript	ENST00000429990	protein_coding	c.1180G>A	p.Ala394Thr	Ala	Thr	'1276/1428	'1180/1332	'394/443
89	17117	snp	chr1	26608852	G	A	rs61775089	G/A	'0/1	missense_variant	UBXN11	ENSG00000158062	transcript	ENST00000374222	protein_coding	c.1501C>T	p.Pro501Ser	Pro	Ser	'1966/2043	'1501/1563	'501/520
90	71734	snp	chr1	201180172	G	A	rs12022770	G/A	'0/1	missense_variant	IGFN1	ENSG00000163395	transcript	ENST00000335211	protein_coding	c.6151G>A	p.Gly2051Ser	Gly	Ser	'6281/11810	'6151/11127	'2051/3708
91	513273	snp	chr19	56663058	C	G	rs373247932	C/G	'0/1	missense_variant	AC024580.1	ENSG00000204533	transcript	ENST00000376271	protein_coding	c.193G>C	p.Ala65Pro	Ala	Pro	'193/717	'193/717	'65/238
92	368683	snp	chr16	74425787	G	T	rs375776693	G/T	'0/1	stop_gained	NPIPB15	ENSG00000196436	transcript	ENST00000429990	protein_coding	c.1141G>T	p.Glu381*	Glu	*	'1237/1428	'1141/1332	'381/443
93	445436	complex	chr19	1037679	CCG	TCA	.	CCG/TCA	'0/1	missense_variant	CNN2	ENSG00000064666	transcript	ENST00000565096	protein_coding	c.677_679delCCGin	p.ThrAsp226IleAsn	ThrAsp	IleAsn	'720/1067	'677/897	'226/298
94	1008089	snp	chrGL000193.1	88270	T	A	.	T/A	'0/1	missense_variant	MGC39584	ENSG00000215689	transcript	ENST00000400767	protein_coding	c.8A>T	p.Lys3Ile	Lys	Ile	'106/1117	'8/165	'3/54
95	710831	snp	chr3	195510707	T	G	rs142559357	G/G	'1/1	missense_variant	MUC4	ENSG00000145113	transcript	ENST00000475231	protein_coding	c.7744A>C	p.Thr2582Pro	Thr	Pro	'7816/16273	'7744/16083	'2582/5360
96	584286	ins	chr2	238499863	A	CTTCAGGAG	rs146534321	AATTCTTCAGGAGAC	'0/1	disruptive_inframe_insertion	AC104667.3	ENSG00000234949	transcript	ENST00000452801	protein_coding	c.52_53insATTCTTC	p.Pro17_Ser18insAsr	Pro	18insAsnSerSerGlyA	'54/1301	'53/264	'18/87
97	445435	snp	chr19	1037670	A	G	rs199840457	A/G	'0/1	missense_variant	CNN2	ENSG00000064666	transcript	ENST00000565096	protein_coding	c.668A>G	p.Lys223Arg	Lys	Arg	'711/1067	'668/897	'223/298
98	423188	del	chr17	80274141	GCAGAGGCT	G	.	GGCAGAGGCTGCTG	'0/1	disruptive_inframe_deletion	CD7	ENSG00000173762	transcript	ENST00000578509	protein_coding	c.215_241delACCCG	p.Asp72_Pro80del	Asp	_Pro80del	'634/925	'215/366	'72/121
99	423188	del	chr17	80274141	GCAGAGGCT	G	.	GGCAGAGGCTGCTG	'0/1	disruptive_inframe_deletion	CD7	ENSG00000173762	transcript	ENST00000584284	protein_coding	c.515_541delACCCG	p.Asp172_Pro180del	Asp	_Pro180del	'623/2021	'515/666	'172/221
100	629035	snp	chr21	44836924	A	C	.	A/C	'0/1	missense_variant	SIK1	ENSG00000142178	transcript	ENST00000270162	protein_coding	c.2050T>G	p.Phe684Val	Phe	Val	'2183/4712	'2050/2352	'684/783
101	205492	snp	chr12	50746514	G	C	rs71466905	G/C	'0/1	missense_variant	FAM186A	ENSG00000185958	transcript	ENST00000543111	protein_coding	c.4101C>G	p.His1367Gln	His	Gln	'4239/8200	'4101/7047	'1367/2348
102	445439	complex	chr19	1037715	TGGG	CAGT	.	TGGG/CAGT	'0/1	missense_variant	CNN2	ENSG00000064666	transcript	ENST00000565096	protein_coding	c.713_716delTGGG	p.MetGly238ThrVal	MetGly	ThrVal	'756/1067	'713/897	'238/298

Metastasis (TP02)
Non-synonymous mutations, n= 105

	Variant_ID	Variant_Type	Chromosome	Position	Ref_Seq	Alt_Seq	dbSNP_ID	Tum_Genotype	Tum_Genotype_Code	Effect	Gene_Name	Gene_ID	Feature_Type	Feature_ID	Transcript_Biotype	hgvs_DNA	hgvs_Protein	Protein_Ref	Protein_Alt	cDNA_Position	cds_Position	Protein_Position
1	706385	snp	chr7	116412043	G	A	.	G/A	'0/1	missense_variant&splice_reg	'MET	ENSG00000105976	transcript	ENST00000397752	protein_coding	c.3028G>A	p.Asp1010Asn	Asp	Asn	'3228/6635	'3028/4173	'1010/1390
2	804992	snp	chrMT	13004	A	T	.	A/T	'0/1	missense_variant	'MT-ND5	ENSG00000198786	transcript	ENST00000361567	protein_coding	c.668A>T	p.Lys223Ile	Lys	Ile	'668/1812	'668/1812	'223/603
3	713089	snp	chr7	142723874	C	T	.	C/T	'0/1	missense_variant	'OR9A2	ENSG00000179468	transcript	ENST00000350513	protein_coding	c.346G>A	p.Val116Met	Val	Met	'409/1038	'346/933	'116/310
4	362061	snp	chr19	21713480	C	A	rs532407132	C/A	'0/1	missense_variant	'ZNF429	ENSG00000197013	transcript	ENST00000597078	protein_coding	c.220C>A	p.Pro74Thr	Pro	Thr	'388/1043	'220/369	'74/122
5	479636	snp	chr21	47545888	G	A	rs145140058	G/A	'0/1	missense_variant	'COL6A2	ENSG00000142173	transcript	ENST00000397763	protein_coding	c.2159G>A	p.Arg720His	Arg	His	'2186/3101	'2159/2757	'720/918
6	702392	snp	chr7	100373361	T	A	.	T/A	'0/1	missense_variant	'ZAN	ENSG00000146839	transcript	ENST00000546213	protein_coding	c.1628T>A	p.Ile543Asn	Ile	Asn	'6045/8036	'1628/3555	'543/1184
7	194691	snp	chr13	41373234	A	T	.	A/T	'0/1	missense_variant	'SLC25A15	ENSG00000102743	transcript	ENST00000417731	protein_coding	c.97A>T	p.Met33Leu	Met	Leu	'166/468	'97/399	'33/132
8	718924	del	chr8	3076899	AG	A	.	AG/A	'0/1	frameshift_variant	'CSMD1	ENSG00000183117	transcript	ENST00000539096	protein_coding	c.4549delC	p.Leu1517fs	Leu	fs	'4549/6330	'4549/6330	'1517/2109
9	63092	snp	chr1	230819343	T	C	.	T/C	'0/1	missense_variant	'COG2	ENSG00000135775	transcript	ENST00000546013	protein_coding	c.257T>C	p.Leu86Ser	Leu	Ser	'312/1400	'257/1284	'86/427
10	117923	snp	chr11	33308043	T	C	.	T/C	'0/1	missense_variant	'HIPK3	ENSG00000110422	transcript	ENST00000456517	protein_coding	c.83T>C	p.Val28Ala	Val	Ala	'445/4478	'83/3585	'28/1194
11	811979	snp	chrX	54049260	C	G	.	C/G	'0/1	missense_variant	'PHF8	ENSG00000172943	transcript	ENST00000445025	protein_coding	c.115G>C	p.Glu39Gln	Glu	Gln	'297/475	'115/293	'39/96
12	562370	snp	chr4	62936529	G	A	.	G/A	'0/1	missense_variant	'LPHN3	ENSG00000150471	transcript	ENST00000502815	protein_coding	c.2657G>A	p.Arg886His	Arg	His	'2658/4284	'2657/2754	'886/917
13	713173	snp	chr7	143048720	G	A	.	G/A	'0/1	missense_variant	'CLCN1	ENSG00000188037	transcript	ENST00000343257	protein_coding	c.2629G>A	p.Gly877Arg	Gly	Arg	'2716/3172	'2629/2967	'877/988
14	341992	snp	chr19	1912926	C	G	.	C/G	'0/1	missense_variant	'ADAT3	ENSG00000213638	transcript	ENST00000602400	protein_coding	c.832C>G	p.Leu278Val	Leu	Val	'1060/1578	'832/1056	'278/351
15	120874	snp	chr11	47198391	G	C	.	G/C	'0/1	missense_variant	'ARFGAP2	ENSG00000149182	transcript	ENST00000530596	protein_coding	c.14C>G	p.Pro5Arg	Pro	Arg	'29/534	'14/519	'5/172
16	31156	snp	chr1	94522308	G	C	.	G/C	'0/1	missense_variant	'ABCA4	ENSG00000198691	transcript	ENST00000370225	protein_coding	c.2231C>G	p.Thr744Ser	Thr	Ser	'2318/7309	'2231/6822	'744/2273
17	280999	snp	chr16	67208095	C	T	.	C/T	'0/1	missense_variant	'NOL3	ENSG00000140939	transcript	ENST00000568146	protein_coding	c.23C>T	p.Pro8Leu	Pro	Leu	'76/1351	'23/660	'8/219
18	305562	snp	chr17	37566375	G	T	.	G/T	'0/1	missense_variant	'MED1	ENSG00000125686	transcript	ENST00000300651	protein_coding	c.2099C>A	p.Pro700Gln	Pro	Gln	'2323/5844	'2099/4746	'700/1581
19	383107	snp	chr19	57166539	G	C	.	G/C	'0/1	missense_variant	'SMIM17	ENSG00000268182	transcript	ENST00000598409	protein_coding	c.315G>C	p.Leu105Phe	Leu	Phe	'481/1076	'315/357	'105/118
20	383107	snp	chr19	57166539	G	C	.	G/C	'0/1	missense_variant	'SMIM17	ENSG00000268182	transcript	ENST00000600547	protein_coding	c.238G>C	p.Val80Leu	Val	Leu	'386/573	'238/312	'80/103
21	30380	snp	chr1	91844029	C	A	.	C/A	'0/1	missense_variant	'HFM1	ENSG00000162669	transcript	ENST00000370424	protein_coding	c.204G>T	p.Trp68Cys	Trp	Cys	'434/3675	'204/3345	'68/1114
22	351328	del	chr19	10597378	TG	T	.	TG/T	'0/1	frameshift_variant	'KEAP1	ENSG00000079999	transcript	ENST00000393623	protein_coding	c.1824delC	p.Val608fs	Val	fs	'2071/2648	'1824/1875	'608/624
23	378240	snp	chr19	49950714	C	T	.	C/T	'0/1	missense_variant	'PIH1D1	ENSG00000104872	transcript	ENST00000597415	protein_coding	c.459G>A	p.Met153Ile	Met	Ile	'841/874	'459/492	'153/163
24	383108	mnp	chr19	57166556	GG	AA	.	GG/AA	'0/1	missense_variant	'SMIM17	ENSG00000268182	transcript	ENST00000598409	protein_coding	c.332_333delGGins	p.Gly111Glu	Gly	Glu	'498/1076	'332/357	'111/118
25	383108	mnp	chr19	57166556	GG	AA	.	GG/AA	'0/1	missense_variant	'SMIM17	ENSG00000268182	transcript	ENST00000600547	protein_coding	c.255_256delGGins	p.GlyAsp85GlyAsn	GlyAsp	GlyAsn	'403/573	'255/312	'85/103
26	816389	snp	chrX	100608329	C	T	.	C/T	'0/1	missense_variant	'BTK	ENSG00000010671	transcript	ENST00000372880	protein_coding	c.1233G>A	p.Met411Ile	Met	Ile	'1546/2203	'1233/1452	'411/483
27	377951	ins	chr19	49657889	T	TTCC	rs57199624	TTCC/TTCC	'1/1	disruptive_inframe_insertion	'HRC	ENSG00000130528	transcript	ENST00000595625	protein_coding	c.603_605dupGGA	p.Glu202dup	Glu	dup	'611/2044	'605/2031	'202/676
28	587470	snp	chr4	191003242	G	A	.	G/A	'0/1	missense_variant	'DUX4L4	ENSG00000258834	transcript	ENST00000554637	protein_coding	c.970G>A	p.Glu324Lys	Glu	Lys	'970/1369	'970/1269	'324/422
29	491038	snp	chr22	38610488	C	T	.	C/T	'0/1	missense_variant	'MAFF	ENSG00000185022	transcript	ENST00000407965	protein_coding	c.98C>T	p.Ser33Leu	Ser	Leu	'301/2325	'98/495	'33/164
30	159996	snp	chr12	48880492	G	A	.	G/A	'0/1	missense_variant	'C12orf54	ENSG00000177627	transcript	ENST00000548364	protein_coding	c.118G>A	p.Glu40Lys	Glu	Lys	'175/687	'118/384	'40/127
31	457606	snp	chr20	43057062	A	G	.	A/G	'0/1	missense_variant	'HNF4A	ENSG00000101076	transcript	ENST00000415691	protein_coding	c.1217A>G	p.Asn406Ser	Asn	Ser	'1289/2302	'1217/1395	'406/464
32	657860	snp	chr6	127765276	C	T	.	C/T	'0/1	missense_variant	'KIAA0408	ENSG00000189367	transcript	ENST00000483725	protein_coding	c.2063G>A	p.Arg688Gln	Arg	Gln	'2400/3284	'2063/2085	'688/694
33	173774	snp	chr12	89891065	C	T	.	C/T	'0/1	missense_variant	'POC1B	ENSG00000139323	transcript	ENST00000549035	protein_coding	c.29G>A	p.Arg10Lys	Arg	Lys	'499/2038	'29/1311	'10/436
34	495245	snp	chr22	45923798	G	T	.	G/T	'0/1	missense_variant	'FBLN1	ENSG00000077942	transcript	ENST00000451475	protein_coding	c.213G>T	p.Met71Ile	Met	Ile	'213/544	'213/544	'71/180
35	757750	snp	chr9	33798574	G	A	rs201773718	G/A	'0/1	missense_variant	'PRSS3	ENSG00000010438	transcript	ENST00000379405	protein_coding	c.545G>A	p.Ser182Asn	Ser	Asn	'584/834	'545/744	'182/247
36	700624	ins	chr7	96635420	A	AGCC	rs559903070	AGCC/AGCC	'1/1	disruptive_inframe_insertion	'DLX6	ENSG00000006377	transcript	ENST00000518156	protein_coding	c.158_160dupCGC	p.Pro53dup	Pro	dup	'591/2304	'161/882	'54/293
37	109504	snp	chr11	1017955	C	T	rs113926343	C/T	'0/1	missense_variant	'MUC6	ENSG00000184956	transcript	ENST00000421673	protein_coding	c.4846G>A	p.Val1616Ile	Val	Ile	'4897/8006	'4846/7320	'1616/2439
38	613714	snp	chr5	132158781	C	G	.	C/G	'0/1	missense_variant	'SHROOM1	ENSG00000164403	transcript	ENST00000378676	protein_coding	c.2059G>C	p.Glu687Gln	Glu	Gln	'2059/2352	'2059/2352	'687/783
39	728317	snp	chr8	39009041	G	T	.	G/T	'0/1	missense_variant	'ADAM32	ENSG00000197140	transcript	ENST00000522506	protein_coding	c.499G>T	p.Asp167Tyr	Asp	Tyr	'502/557	'499/554	'167/183
40	381543	complex	chr19	54745989	GTGTC	CTGTT	.	GTGTC/CTGTT	'0/1	missense_variant	'LILRA6	ENSG00000244482	transcript	ENST00000245621	protein_coding	c.264_268delGACA	p.MetThrGln88IleThr	MetThrGln	IleThrGlu	'268/1395	'264/1395	'88/464
41	712706	snp	chr7	142148963	C	T	rs371195527	C/T	'0/1	missense_variant	'TRBV5-5	ENSG00000211725	transcript	ENST00000390372	TR_V_gene	c.308G>A	p.Gly103Glu	Gly	Glu	'315/350	'308/343	'103/113
42	377950	del	chr19	49657710	ACAT	A	rs66501117	A/A	'1/1	disruptive_inframe_deletion	'HRC	ENSG00000130528	transcript	ENST00000595625	protein_coding	c.782_784delATG	p.Asp261del	Asp	del	'790/2044	'782/2031	'261/676
43	587465	snp	chr4	191003198	G	T	.	G/T	'0/1	missense_variant	'DUX4L4	ENSG00000258834	transcript	ENST00000554637	protein_coding	c.926G>T	p.Trp309Leu	Trp	Leu	'926/1369	'926/1269	'309/422
44	299098	snp	chr17	21319079	C	A	rs76518282	C/A	'0/1	missense_variant	'KCNJ12	ENSG00000184185	transcript	ENST00000583088	protein_coding	c.425C>A	p.Thr142Asn	Thr	Asn	'1320/5420	'425/1302	'142/433
45	150946	snp	chr12	11286807	T	C	rs113282241	T/C	'0/1	missense_variant	'TAS2R30	ENSG00000256188	transcript	ENST00000539585	protein_coding	c.37A>G	p.Ile13Val	Ile	Val	'437/1687	'37/960	'13/319
46	160178	snp	chr12	49229958	G	A	.	G/A	'0/1	missense_variant	'DDX23	ENSG00000174243	transcript	ENST00000308025	protein_coding	c.1328C>T	p.Ala443Val	Ala	Val	'1408/3248	'1328/2463	'443/820
47	483540	snp	chr22	22707564	G	C	rs539733792	G/C	'0/1	missense_variant	'IGLV5-48	ENSG00000211647	transcript	ENST00000390293	IG_V_gene	c.152G>C	p.Ser51Thr	Ser	Thr	'152/369	'152/369	'51/122
48	109498	snp	chr11	1017307	G	A	rs372717767	G/A	'0/1	missense_variant	'MUC6	ENSG00000184956	transcript	ENST00000421673	protein_coding	c.5494C>T	p.Pro1832Ser	Pro	Ser	'5545/8006	'5494/7320	'1832/2439
49	288762	snp	chr16	88791458	G	A	rs11645197	G/A	'0/1	missense_variant	'PIEZO1	ENSG00000103335	transcript	ENST00000301015	protein_coding	c.4193C>T	p.Pro1398Leu	Pro	Leu	'4440/8072	'4193/7566	'1398/2521
50	712707	mnp	chr7	142148969	AA	TC	.	AA/TC	'0/1	missense_variant	'TRBV5-5	ENSG00000211725	transcript	ENST00000390372	TR_V_gene	c.301_302delTTins	p.Leu101Glu	Leu	Glu	'309/350	'301/343	'101/113
51	378646	snp	chr19	50357709	C	T	.	C/T	'0/1	missense_variant	'PTOV1	ENSG00000104960	transcript	ENST00000600793	protein_coding	c.47C>T	p.Ser16Leu	Ser	Leu	'47/426	'47/426	'16/141
52	616184	snp	chr5	140221195	G	C	rs199713478	G/C	'0/1	missense_variant	'PCDHA8	ENSG00000204962	transcript	ENST00000378123	protein_coding	c.289G>C	p.Gly97Arg	Gly	Arg	'289/2445	'289/2445	'97/814
53	348160	del	chr19	7696610	TC	T	.	TC/T	'0/1	frameshift_variant	'PCP2	ENSG00000174788	transcript	ENST00000598935	protein_coding	c.327delG	p.Arg109fs	Arg	fs	'343/457	'327/363	'109/120
54	150945	snp	chr12	11286797	A	G	.	A/G	'0/1	missense_variant	'TAS2R30	ENSG00000256188	transcript	ENST00000539585	protein_coding	c.47T>C	p.Ile16Thr	Ile	Thr	'447/1687	'47/960	'16/319
55	549567	snp	chr4	16597450	C	A	.	C/A	'0/1	missense_variant	'LDB2	ENSG00000169744	transcript	ENST00000506732	protein_coding	c.212G>T	p.Gly71Val	Gly	Val	'286/567	'212/493	'71/163
56	496610	snp	chr22	49246651	G	A	.	G/A	'0/1	missense_variant	'FAM19A5	ENSG00000219438	transcript	ENST00000336769	protein_coding	c.472G>A	p.Asp158Asn	Asp	Asn	'473/546	'472/545	'158/180
57	712789	mnp	chr7	142224021	GCA	TTG	.	GCA/TTG	'0/1	missense_variant	'TRBV11-1	ENSG00000211720	transcript	ENST00000390367	TR_V_gene	c.144_146delTGCin	p.HisAla48HisAsn	HisAla	HisAsn	'163/364	'144/347	'48/114
58	7964	snp	chr1	16918473	G	A	rs60465633	G/A	'0/1	missense_variant	'NBPF1	ENSG00000219481	transcript	ENST00000430580	protein_coding	c.44C>T	p.Thr15Met	Thr	Met	'932/5932	'44/3420	'15/1139
59	811925	snp	chrX	53652846	G	T	rs74381511	G/T	'0/1	missense_variant	'HUWE1	ENSG00000086758	transcript	ENST00000424562	protein_coding	c.424C>A	p.Pro142Thr	Pro	Thr	'425/682	'424/681	'142/226
60	517281	snp	chr3	75787702	C	G	rs62250110	C/G	'0/1	missense_variant	'ZNF717	ENSG00000227124	transcript	ENST00000400845	protein_coding	c.1051G>C	p.Glu351Gln	Glu	Gln	'1339/3012	'1051/2724	'351/907
61	565485	snp	chr4	79369398	A	T	.	A/T	'0/1	stop_gained	'FRAS1	ENSG00000138759	transcript	ENST00000512123	protein_coding	c.886A>T	p.Lys296*	Lys	*	'887/9869	'886/6723	'296/2240
62	211701	ins	chr14	24769849	A	AGAGGAG	rs71119069	GAGGAG/AGAGGA	'1/1	disruptive_inframe_insertion	'NOP9	ENSG00000196943	transcript	ENST00000396802	protein_coding	c.504_509dupGGA	p.Glu168_Glu169dup	Glu	_Glu169dup	'586/1942	'510/1608	'170/535
63	712828	snp	chr7	142247546	C	A	rs575564328	C/A	'0/1	missense_variant	'TRBV7-3	ENSG00000211714	transcript	ENST00000390361	TR_V_gene	c.20G>T	p.Cys7Phe	Cys	Phe	'70/397	'20/347	'7/114
64	517280	snp	chr3	75787620	T	C	rs75737034	T/C	'0/1	missense_variant	'ZNF717	ENSG00000227124	transcript	ENST00000400845	protein_coding	c.1133A>G	p.His378Arg	His	Arg	'1421/3012	'1133/2724	'378/907
65	76117	snp	chr10	29783908	A	G	rs78773460	A/G	'0/1	missense_variant	'SVIL	ENSG00000197321	transcript	ENST00000538146	protein_coding	c.152T>C	p.Met51Thr	Met	Thr	'234/2211	'152/1758	'51/585
66	589303	snp	chr5	5303424	G	A	rs34586473	G/A	'0/1	missense_variant	'ADAMTS16	ENSG00000145536	transcript	ENST00000274181	protein_coding	c.2833G>A	p.Gly945Arg	Gly	Arg	'2971/4979	'2833/3675	'945/1224
67	375218	ins	chr19	46265047	A	ATCCAGC	rs59054027	ATCCAGC/ATCCAGC	'1/1	disruptive_inframe_insertion	'AC074212.3	ENSG00000237452	transcript	ENST00000457052	protein_coding	c.1389_1394dupCA	p.Ser464_Ser465dup	Ser	_Ser465dup	'1811/2749	'1395/1917	'465/638
68	319278	snp	chr17	74017771	G	A	.	G/A	'0/1	missense_variant	'EVPL	ENSG00000167880	transcript	ENST00000301607	protein_coding	c.899C>T	p.Ala300Val	Ala	Val	'1153/6614	'899/6102	'300/2033
69	258148	complex	chr16	3119304	A	GG	.	A/GG	'0/1	frameshift_variant&missense	'IL32	ENSG00000008517	transcript	ENST00000548246	protein_coding	c.395delAinsGG	p.Asp132fs	Asp	fs	'395/447	'395/447	'132/148
70	297664	del	chr17	18380163	GGA	G	rs376412531	GGA/G	'0/1	frameshift_variant&start_los	'LGALS9C	ENSG00000171916	transcript	ENST00000584941	protein_coding	c.-1_1delGA	p.Met1fs	Met	fs	'57/1104	'1/705	'1/234
71	757550	complex	chr9	33386465	AGACC	GGACT	.	AGACC/GGACT	'0/1	missense_variant	'AQP7	ENSG00000165269	transcript	ENST00000379503	protein_coding	c.147_151delGGTC	p.ProValTyr49ProVal	ProValTyr	ProValHis	'193/677	'147/582	'49/193
72	349747	snp	chr19	9009652	C	T	.	C/T	'0/1	missense_variant	'MUC16	ENSG00000181143	transcript	ENST00000397910	protein_coding	c.39074G>A	p.Gly13025Glu	Gly	Glu	'39278/43816	'39074/43524	'13025/14507
73	381540	snp	chr19	54745682	C	T	rs111666280	C/T	'0/1	missense_variant	'LILRA6	ENSG00000244482	transcript	ENST00000245621	protein_coding	c.428G>A	p.Arg143Gln	Arg	Gln	'428/1395	'428/1395	'143/464
74	322701	del	chr17	80274141	GCAGAGGCT	G	.	GGCAGAGGCTGCTG	'0/1	disruptive_inframe_deletion	'CD7	ENSG00000173762	transcript	ENST00000578509	protein_coding	c.215_241delACCCG	p.Asp72_Pro80del	Asp	_Pro80del	'634/925	'215/366	'72/121
75	322701	del	chr17	80274141	GCAGAGGCT	G	.	GGCAGAGGCTGCTG	'0/1	disruptive_inframe_deletion	'CD7	ENSG00000173762	transcript	ENST00000584284	protein_coding	c.515_541delACCCG	p.Asp172_Pro180del	Asp	_Pro180del	'623/2021	'515/666	'172/221
76	445818	snp	chr20	4893560	G	T	.	G/T	'0/1	missense_variant	'SLC23A2	ENSG00000089057	transcript	ENST00000424750	protein_coding	c.173C>A	p.Ala58Glu	Ala	Glu	'556/2375	'173/1611	'58/536
77	383606	snp	chr19	58385748	G	A	rs145250945	G/A	'0/1	missense_variant	'ZNF814	ENSG00000204514	transcript	ENST00000435989	protein_coding	c.1010C>T	p.Ala337Val	Ala	Val	'1245/3146	'1010/2568	'337/855
78	4184	del	chr1	8716321	CTCTTTG	C	rs3831914	CTCTTTG/C	'0/1	disruptive_inframe_deletion	'RERE	ENSG00000142599	transcript	ENST00000468247	protein_coding	c.30_35delCAAAGA	p.Asp10_Lys11del	Asp	_Lys11del	'344/414	'30/105	'10/34
79	361389	snp	chr19	20989236	G	T	rs557992660	G/T	'0/1	missense_variant	'ZNF66	ENSG00000160229	transcript	ENST00000425625	protein_coding	c.968G>T	p.Arg323Ile	Arg	Ile	'968/1338	'968/1338	'323/445
80	417838	snp	chr2	130951754	G	T	rs13000249	G/T	'0/1	missense_variant	'TUBA3E	ENSG00000152086	transcript	ENST00000312988	protein_coding	c.661C>A	p.Arg221Ser	Arg	Ser	'762/1540	'661/1353	'221/450
81	820688	complex	chrX	153006137	GTCGT	ATCGA	.	GTCGT/ATCGA	'0/1	missense_variant	'ABCD1	ENSG00000101986	transcript	ENST00000218104	protein_coding	c.1744_1748delGTC	p.ValVal582IleGlu	ValVal	IleGlu	'2143/3664	'1744/2238	'582/745
82	108177	snp	chr10	135012492	A	C	.	A/C	'0/1	missense_variant	'KNDC1	ENSG00000171798	transcript	ENST00000368571	protein_coding	c.2285A>C	p.His762Pro	His	Pro	'2575/3925	'2285/3426	'762/1141
83	209004	snp	chr14	19553795	G	A	rs149181731	G/A	'0/1	missense_variant	'POTEG	ENSG00000222036	transcript	ENST00000409832	protein_coding	c.379G>A	p.Ala127Thr	Ala	Thr	'431/7062	'379/1527	'127/508
84	54797	snp	chr1	201179089	G	A	rs11576220	G/A	'0/1	missense_variant	'IGFN1	ENSG00000163395	transcript	ENST00000335211	protein_coding	c.5068G>A	p.Ala1690Thr	Ala	Thr	'5198/11810	'5068/11127	'1690/3708
85	542669	snp	chr3	195515470	T	G	rs199696565	T/G	'0/1	missense_variant	'MUC4	ENSG00000145113	transcript	ENST00000475231	protein_coding	c.2981A>C	p.His994Pro	His	Pro	'3053/16273	'2981/16083	'994/5360
86	623861	snp	chr5	173035291	G	A	rs74729929	G/A	'0/1	missense_variant	'BOD1	ENSG00000145919	transcript	ENST00000285908	protein_coding	c.367C>T	p.Arg123Cys	Arg	Cys	'519/1286	'367/390	'123/129
87	623861	snp	chr5	173035291	G	A	rs74729929	G/A	'0/1	missense_variant	'BOD1	ENSG00000145919	transcript	ENST00000480951	protein_coding	c.242C>T	p.Thr81Met	Thr	Met	'459/814	'242/258	'81/85
88	418749	snp	chr2	132912341	C	T	rs201696527	C/T	'0/1	missense_variant&splice_reg	'ANKRD30BL	ENSG00000163046	transcript	ENST00000409867	protein_coding	c.508G>A	p.Ala170Thr	Ala	Thr	'758/1251	'508/777	'170/258
89	12536	snp	chr1	26608852	G	A	rs61775089	G/A	'0/1	missense_variant	'UBXN11	ENSG00000158062	transcript	ENST00000374222	protein_coding	c.1501C>T	p.Pro501Ser	Pro	Ser	'1966/2043	'1501/1563	'501/520
90	820687	mnp	chrX	153006092	CA	TG	.	CA/TG	'0/1	stop_gained	'ABCD1	ENSG00000101986	transcript	ENST00000218104	protein_coding	c.1699_1700delCAi	p.Gln567*	Gln	*	'2098/3664	'1699/2238	'567/745
91	120528	snp	chr11	46369238	C	A	rs76656670	C/A	'0/1	missense_variant	'DGKZ	ENSG00000149091	transcript	ENST00000318201	protein_coding	c.29C>A	p.Ala10Asp	Ala	Asp	'101/3213	'29/2721	'10/906
92	67809	snp	chr1	248458717	C	G	rs12745228	C/G	'0/1	missense_variant	'OR2T12	ENSG00000177201	transcript	ENST00000317996	protein_coding	c.164G>C	p.Arg55Thr	Arg	Thr	'164/963	'164/963	'55/320
93	637116	snp	chr6	32610495	A	G	.	A/G	'0/1	missense_variant	'HLA-DQA1	ENSG00000196735	transcript	ENST00000374949	protein_coding	c.722A>G	p.Gln241Arg	Gln	Arg	'761/1060	'722/768	'241/255
94	418186	snp	chr2	132021946	G	A	rs62178369	G/A	'0/1	missense_variant	'POTEE	ENSG00000188219	transcript	ENST00000356920	protein_coding	c.2918G>A	p.Gly973Asp	Gly	Asp	'3012/4031	'2918/3228	'973/1075
95	340333	complex	chr19	1037679	CCG	TCA	.	CCG/TCA	'0/1	missense_variant	'CNN2	ENSG00000064666	transcript	ENST00000565096	protein_coding	c.677_679delCCGin	p.ThrAsp226IleAsn	ThrAsp	IleAsn	'720/1067	'677/897	'226/298
96	39932	snp	chr1	145361463	C	A	rs201261741	C/A	'0/1	missense_variant	'NBPF10	ENSG00000163386	transcript	ENST00000342960	protein_coding	c.9367C>A	p.Gln3123Lys	Gln	Lys	'9402/10697	'9367/10662	'3123/3553
97	233662	del	chr15	23086364	GGCC	G	rs531550505	GGCC/G	'0/1	disruptive_inframe_deletion	'NIPA1	ENSG00000170113	transcript	ENST00000337435	protein_coding	c.45_47delGGC	p.Ala16del	Ala	del	'72/6567	'45/990	'15/329
98	340336	complex	chr19	1037715	TGGG	CAGT	.	TGGG/CAGT	'0/1	missense_variant	'CNN2	ENSG00000064666	transcript	ENST00000565096	protein_coding	c.713_716delTGGG	p.MetGly238ThrVal	MetGly	ThrVal	'756/1067	'713/897	'238/298
99	340332	snp	chr19	1037670	A	G	rs199840457	A/G	'0/1	missense_variant	'CNN2	ENSG00000064666	transcript	ENST00000565096	protein_coding	c.668A>G	p.Lys223Arg	Lys	Arg	'711/1067	'668/897	'223/298
100	340339	snp	chr19	1037801	A	C	rs78797154	A/C	'0/1	missense_variant	'CNN2	ENSG00000064666	transcript	ENST00000565096	protein_coding	c.799A>C	p.Thr267Pro	Thr	Pro	'842/1067	'799/897	'267/298
101	542423	snp	chr3	195507332	A	T	rs200058677	A/T	'0/1	missense_variant	'MUC4	ENSG00000145113	transcript	ENST00000475231	protein_coding	c.11119T>A	p.Ser3707Thr	Ser	Thr	'11191/16273	'11119/16083	'3707/5360
102	787393	snp	chrGL000193.1	88206	C	A	.	C/A	'0/1	missense_variant	'MGC39584	ENSG00000215689	transcript	ENST00000400767	protein_coding	c.72G>T	p.Leu24Phe	Leu	Phe	'170/1117	'72/165	'24/54
103	252637	del	chr15	90320120	GGGGCAAGG	G	rs200021459	AGGGGCAAGGGCA	'0/1	disruptive_inframe_deletion	'MESP2	ENSG00000188095	transcript	ENST00000341735	protein_coding	c.558_581delGGGG	p.Gly187_Gln194del	Gly	_Gln194del	'558/1552	'558/1194	'186/397
104	542440	snp	chr3	195507827	G	A	rs373469782	G/A	'0/1	missense_variant	'MUC4	ENSG00000145113	transcript	ENST00000475231	protein_coding	c.10624C>T	p.Leu3542Phe	Leu	Phe	'10696/16273	'10624/16083	'3542/5360
105	479737	snp	chr21	47613570	T	C	rs5025856	T/C	'0/1	missense_variant	'AP001468.1	ENSG00000235878	transcript	ENST00000594486	protein_coding	c.104A>G	p.His35Arg	His	Arg	'104/804	'104/804	'35/267
