# Supplementary material for: Targeting the tumor mutanome for personalized vaccination in a TMB low non-small cell lung cancer
Source: J Immunother Cancer. 2022 Mar 30;10(3):e003821. doi: 10.1136/jitc-2021-003821 (PMC8971766; doi:10.1136/jitc-2021-003821)
Supplement: Supplementary data [file jitc-2021-003821supp003.pdf]

CD8 KIAA0408 MUT - specific CDR3 Vβ				
**CDR3 nucleotide sequence**	**Vβ**	**TCR reads**	**Primary**	**Metastasis**
TGTGCCAGTAGTATAGGTGGCAGCTCCTACAATGAGCAGTTCTTC	TRBV19*01	15		1
TGCAGTGCTAGCAGACTAGCAGGGGACAATGAGCAGTTCTTC	TRBV20-1*01	1		1
TGTGCCAGCAGTTACCAGGGGCCCACTTATGGCTACACCTTC	TRBV6-6*01	1		1
TGTGCCAGCAGCGTCGGGACAGGGGTCCACGAGCAGTACTTC	TRBV9*01	1		1
TGTGCCAGCAGCCCAGGGGTGGGCTACACCTTC	TRBV27*01	1	1	
TGTGCCAGCAGTCCGTGGGCGGGAGCAGATACGCAGTATTTT	TRBV12-3*01,TRBV12-4*01	1	1	
TGCGCCAGCAGCCAAGATCGAAGGGGGGGAAACTACGAGCAGTACTTC	TRBV4-3*01	1	1	
TGCGCCAGCAGCGGTGCGGGGGGGTTGGAGACCCAGTACTTC	TRBV5-1*01	1	7	2
TGTGCCAGCAGTTATTCCGGGACTAGCGGGATTGCCGATGAGCAGTTCTTC	TRBV6-6*01	27		
TGTGCCAGCAGCCAAGATTGGGTAGAGCAGTTCTTC	TRBV14*01	13		
TGTGCCAGCTCACTAGACCTGAACACTGAAGCTTTCTTT	TRBV18*01	5		
TGTGCCAGCAGCTTCTCCGTGGGGACAGGTCAAAGCGAGCAGTACTTC	TRBV13*01	5		
TGTGCCAGCAGCGCCGGGACAGGGGCCTACGAGCAGTACTTC	TRBV2*01	4		
TGTGCCAGTAGTTTGGGACCGATTAACACTGAAGCTTTCTTT	TRBV19*01	4		
TGCAGTGCTAGATCTCTGGGAGCAATAGTGGAGCAGTACTTC	TRBV20-1*01	4		
TGTGCCAGCAGCCTTGTGTCCGAGCAGTACTTC	TRBV11-2*01	3		
TGTGCCAGCAGCTTTGCCGGGACAGGGATGTCCTACGAGCAGTACTTC	TRBV5-6*01	2		
TGCAGTGCTAGAGATTCAGGACTAGCGATCTCCTACGAGCAGTACTTC	TRBV20-1*01	2		
TGTGCCAGCAGCCCCGTTAGCGAAAATGAGCAGTTCTTC	TRBV7-2*01	2		
TGTGCCAGCAGCGGGGAGCGGGAGTGGGCCGGGGAGCTGTTTTTT	TRBV7-8*01	2		
TGTGCCAGCAGCTTAGCGGGAGGGGCCCTCCGCTACAATGAGCAGTTCTTC	TRBV7-2*01	2		
TGCAGCGTTGAACCTAGCGGGAATACCGGGGAGCTGTTTTTT	TRBV29-1*01	2		
TGCAGTGCCCATCGACTAGAGCAGTTCTTC	TRBV20-1*01	2		
TGCAGTGCGGACAGTGGGACCGGGACAGGGGGTTCACCCTACGAGCAGTACTTC	TRBV20-1*01	2		
TGCAGTGCTAGAGATGGAACAGGGGGCGGTGAGCAGTTCTTC	TRBV20-1*01	2		
TGCAGTGCTAGAGCAGTCAGCTCCTACGAGCAGTACTTC	TRBV20-1*01	2		
TGTGCCAGCAGCCAAGGGGGAACTGAAGCTTTCTTT	TRBV16*01	2		
TGTGCCAGCAGTTATTCCGGGACTAGCGGGATTGCCGATGAGCAGTTCTTC	TRBV6-9*01	2		
TGTGCCAGCAGTTTGGCTAGTTATACAGATACGCAGTATTTT	TRBV28*01	2		
TGTGCCAGCGCACGGAGCTCCTACAATGAGCAGTTCTTC	TRBV7-2*01	2		
TGTGCCAGCAGCCAAACCCCCCACGGGGAGGAGGGACCGGGGAGCTGTTTTTT	TRBV6-5*01	2		
TGTGCCAGCAGCCAGATAGCGGGAGAAATCTACACCGGGGAGCTGTTTTTT	TRBV2*01	2		
TGTGCCAGCAGCCCCGGACAGGGGGGGAGGCCAGTACTTC	TRBV3-1*01	2		
TGCAGCGTTGAATTCAGTGGGAGCTCCTACGAGCAGTACTTC	TRBV29-1*01	2		
TGTGCCAGCACCGTTAGGCGGTCTTGGCAGCCCCAGCATTTT	TRBV7-3*01	2		
TGTGCCAGCAGTTACGAAGCCCGGGCATTCGATGAGCTGTTTTTT	TRBV6-5*01	2		
TGCGCCAGCAGCTTGGCAGGTCATAACACCGGGGAGCTGTTTTTT	TRBV5-1*01	1		
TGCAGCACTGGGGGGGGGAACACCGGGGAGCTGTTTTTT	TRBV29-1*01	1		
TGTGCCAGCAGCTACGGTACGGGGGAGCAGTACTTC	TRBV3-1*01	1		
TGTGCCAGCAGTGATTATGATACCAGCAATCAGCCCCAGCATTTT	TRBV6-1*01	1		
TGCAGTGCTAGAGAAGGGGGCCCCAAAAACATTCAGTACTTC	TRBV20-1*01	1		
TGCGCCAGCAGCATAGGGTTAACCGGGGAGCTGTTTTTT	TRBV5-1*01	1		
TGCGCCAGCAGCCATGAGCTAGCCCCAGATACGCAGTATTTT	TRBV5-1*01	1		
TGTGCCAGTAGTATTTGGAGCGGGGATTACAATGAGCAGTTCTTC	TRBV19*01	1		
TGTGCCAGCAGCCAAGCCGGGACAGGATTCCACGAGCAGTACTTC	TRBV3-1*01	1		
TGCGCCAGCAGCCAAGATGCCAGTACCTCCTACAATGAGCAGTTCTTC	TRBV4-3*01	1		
TGCGCCAGCAGCCCCCCCATGGGGAGGGCGGAAAATGAGCAGTTCTTC	TRBV4-1*01	1		
TGCGCCAGCAGCTCGGGACGCAACTCCTACGAGCAGTACTTC	TRBV5-1*01	1		
TGCGCCGGGAGCGATCCGAACACCGGGGAGCTGTTTTTT	TRBV5-1*01	1		
TGTGCCAGCAGCCACCGCCTGGGTAGTAGCTCCTACAATGAGCAGTTCTTC	TRBV7-9*01	1		
TGTGCCAGCAGCGTAGCTACCGGGGAGCTGTTTTTT	TRBV9*01	1		
TGTGCCAGCAGCTTATCGGGCGGGCCCCAAGAGACCCAGTACTTC	TRBV11-3*01	1		
TGCAGCGTAGCGGGAGTCGGCTACGAGCAGTACTTC	TRBV29-1*01	1		
TGCAGCGTCCTTAACAGGAACCTTAACTATGGCTACACCTTC	TRBV29-1*01	1		
TGCAGCGTGAGGCTGGCCGGGACTAGCGGGAGGAGTGATGAGCAGTTCTTC	TRBV29-1*01	1		
TGCAGCGTTCCAATGAACACTGAAGCTTTCTTT	TRBV29-1*01	1		
TGCAGCGTTGAACGGGGGGCCTATGAGCAGTTCTTC	TRBV29-1*01	1		
TGCAGCGTTGACGGGGGGGTGGCAGGGGATCCCCTTGGAGGCTACACCTTC	TRBV29-1*01	1		
TGCAGCGTTGAGGATCGGGAACTCTCTGGAAACACCATATATTTT	TRBV29-1*01	1		
TGCAGCGTTGAGGGCAGGGGGTTGGACACCGGGGAGCTGTTTTTT	TRBV29-1*01	1		
TGCAGCGTTGAGGGGGGCCGCAACTACGAGCAGTACTTC	TRBV29-1*01	1		
TGCAGCGTTGAGGGGGGGCTGGACAGGGGGCTTAATGAGCAGTTCTTC	TRBV29-1*01	1		
TGCAGCGTTGCGACTAGCGGGGAATTCGGGGAGCTGTTTTTT	TRBV29-1*01	1		
TGCAGCGTTGTCGAGCCTTACTCCTACGAGCAGTACTTC	TRBV29-1*01	1		
TGCAGCGTTGTCGGATATGGGAGGGAGGGACCCGGGGAGCTGTTTTTT	TRBV29-1*01	1		
TGCAGCGTTGTGGGTGAGGGGGGCTGGTACGAGCAGTACTTC	TRBV29-1*01	1		
TGCAGCGTTTTCCTTAGCGGGGGCCGTAGCACAGATACGCAGTATTTT	TRBV29-1*01	1		
TGCAGTGCCCTTCCGGACAGTGGCTACGAGCAGTACTTC	TRBV20-1*01	1		
TGCAGTGCTACGACTAGCGGAACCTACAATGAGCAGTTCTTC	TRBV20-1*01	1		
TGCAGTGCTAGAGATATCCTAGCGCCTACCTACGAGCAGTACTTC	TRBV20-1*01	1		
TGCAGTGCTAGAGATATGGCAGGGGGTGACTACGAGCAGTACTTC	TRBV20-1*01	1		
TGCAGTGCTAGAGATCTAATAGCGTCTTACGAGCAGTACTTC	TRBV20-1*01	1		
TGCAGTGCTAGAGGCTGGACTAGCGGCCAAGAGACCCAGTACTTC	TRBV20-1*01	1		
TGCAGTGCTAGGTTGGCAGGGAGCTCCTACGAGCAGTACTTC	TRBV20-1*01	1		
TGCAGTGCTAGTCTTGGACTAGCTGACGAGCAGTACTTC	TRBV20-1*01	1		
TGCAGTGCTCCCGGACTAGCGGAGGGAGTTGACTACAATGAGCAGTTCTTC	TRBV20-1*01	1		

TGCAGTGCTTTATTAGATACGCAGTATTTT	TRBV20-1*01	1		
TGCGCCAGAGGAAACACCATATATTTT	TRBV10-1*01	1		
TGCGCCAGCACCGACAGGGAAGGGGAAAACATTCAGTACTTC	TRBV5-1*01	1		
TGCGCCAGCAGCAGTACAGGGAATGCCTACGAGCAGTACTTC	TRBV5-1*01	1		
TGCGCCAGCAGCCAAGAATGGGCATTGAACACTGAAGCTTTCTTT	TRBV4-1*01	1		
TGCGCCAGCAGCCAAGAGCCCAGCACAGATACGCAGTATTTT	TRBV4-1*01	1		
TGCGCCAGCAGCCAAGAGGCGACTAGCGCCTACGAGCAGTACTTC	TRBV4-1*01	1		
TGCGCCAGCAGCCAAGATCGGGCGACTCCTGGGCTGGTGGGCTACACCTTC	TRBV4-3*01	1		
TGCGCCAGCAGCCAAGATGGGGTGAATGTTAATTCACCCCTCCACTTT	TRBV4-3*01	1		
TGCGCCAGCAGCCAAGCCAGGGGGCGCGCCGAGCAGTACTTC	TRBV4-1*01	1		
TGCGCCAGCAGCCAAGCCCAGGGGGCCGGAGAGCAGTACTTC	TRBV4-3*01	1		
TGCGCCAGCAGCCAAGGAGGCAGCTCTGGAAACACCATATATTTT	TRBV4-3*01	1		
TGCGCCAGCAGCCAAGGCGGACTAGCGGGAGTTTCAGATACGCAGTATTTT	TRBV4-1*01	1		
TGCGCCAGCAGCCAGGCTTACCAGCAAAATGAGCAGTTCTTC	TRBV4-3*01	1		
TGCGCCAGCAGCCAGGGGCCATTCGATGAGCAGTTCTTC	TRBV4-1*01	1		
TGCGCCAGCAGCCATAGGACAGGGGAGTTTGAAGCTTTCTTT	TRBV4-3*01	1		
TGCGCCAGCAGCCCCACCGGGCCCTGGGGCTACAATGAGCAGTTCTTC	TRBV5-1*01	1		
TGCGCCAGCAGCCCTTGGGAGACGGGGTGGGCGGAGACCCAGTACTTC	TRBV4-1*01	1		
TGCGCCAGCAGCCGCTCGACAGGGGGCGGGGAGACCCAGTACTTC	TRBV4-1*01	1		
TGCGCCAGCAGCCTCCAGGGGGCTGAAGCTTTCTTT	TRBV4-1*01	1		
TGCGCCAGCAGCCTGACAGGGGGCACCGGGGAGCTGTTTTTT	TRBV4-1*01	1		
TGCGCCAGCAGCTATCACTGGGAGGATAGCGGGGCAAACTACGAGCAGTACTTC	TRBV4-3*01	1		
TGCGCCAGCAGCTCCATAGGACAGGGGATGGACAATGAGCAGTTCTTC	TRBV5-1*01	1		
TGCGCCAGCAGCTCCCAGGGAGTGAGGGGCTACACCTTC	TRBV5-1*01	1		
TGCGCCAGCAGCTCCTACAATGAGCAGTTCTTC	TRBV5-1*01	1		
TGCGCCAGCAGCTCGACTAGCAGTGAGCAGTTCTTC	TRBV5-1*01	1		
TGCGCCAGCAGCTGGAGCGGAACAGATACGCAGTATTTT	TRBV5-1*01	1		
TGCGCCAGCAGCTTCAGCGGGGGAGCCTTGGAGCAGTACTTC	TRBV5-1*01	1		
TGCGCCAGCAGCTTCGGGGGGCACACTGAAGCTTTCTTT	TRBV5-1*01	1		
TGCGCCAGCAGCTTGCGGGAGGGCCCGAGCGAGCAGTACTTC	TRBV5-1*01	1		
TGCGCCAGCAGCTTGGAAGGCATCCCCAATCAGCCCCAGCATTTT	TRBV5-1*01	1		
TGCGCCAGCAGCTTGGAAGGGGTCCCCGGGGAGCTGTTTTTT	TRBV5-1*01	1		
TGCGCCAGCAGCTTGGGACAGGGGTATAACAATGAGCAGTTCTTC	TRBV5-1*01	1		
TGCGCCAGCAGCTTGGGACTTGACAGTACAGAAGAGACCCAGTACTTC	TRBV5-1*01	1		
TGCGCCAGCAGCTTGGGAGGACTAGCAAATGAGCAGTTCTTC	TRBV5-1*01	1		
TGCGCCAGCAGCTTGGTTCTGAACGACGAGCAGTACTTC	TRBV5-1*01	1		
TGCGCCAGCAGCTTGTTGCCAGGGACTACCTACAATGAGCAGTTCTTC	TRBV5-1*01	1		
TGCGCCAGCAGCTTGTTGGGATCCGGAAACACCATATATTTT	TRBV5-1*01	1		
TGCGCCAGCAGTCAATCCGGGGCTCGCCACGAGCAGTACTTC	TRBV4-3*01	1		
TGCGCCAGCAGTCATTCATTCACACAGGGGGAAAACGAAGCTTTCTTT	TRBV5-1*01	1		
TGTGCCACCACCTCGACGGGTTCCAACACTGAAGCTTTCTTT	TRBV6-6*01	1		
TGTGCCACCAGCAGAGATAGAGGGACAGGGGGCTACACCGGGGAGCTGTTTTTT	TRBV15*01	1		
TGTGCCACCAGCAGAGGAGCGGGTTCCTACAATGAGCAGTTCTTC	TRBV15*01	1		
TGTGCCACCAGCAGAGGGGACAGGGGAGACGAGCAGTACTTC	TRBV15*01	1		
TGTGCCACCAGCAGAGTTAGCTCCTACGAGCAGTACTTC	TRBV15*01	1		
TGTGCCACCAGCAGCGACAGGGGAAACACCATATATTTT	TRBV15*01	1		
TGTGCCACCAGTCGACAGGGGTACAATGAGCAGTTCTTC	TRBV24-1*01	1		
TGTGCCACCAGTGAAGGGACACCTACCTACAATGAGCAGTTCTTC	TRBV24-1*01	1		
TGTGCCACCAGTGATTCCGACGGGAACACTGAAGCTTTCTTT	TRBV24-1*01	1		
TGTGCCACCAGTGATTCGAGAACCGGGGAGCTGTTTTTT	TRBV24-1*01	1		
TGTGCCACCAGTGATTTGTGGGGGAAGCAGTACTTC	TRBV24-1*01	1		
TGTGCCAGCAAACCTACTAGCACAGATACGCAGTATTTT	TRBV2*01	1		
TGTGCCAGCAAATCAGACGGGGGGCCCGGGGAGCTGTTTTTT	TRBV6-1*01	1		
TGTGCCAGCAACATCGGACGGTGGACTGAAGCTTTCTTT	TRBV12-3*01,TRBV12-4*01	1		
TGTGCCAGCACCACGACAGGGGGCGGTGAGCAGTTCTTC	TRBV28*01	1		
TGTGCCAGCACCCAAGTCGGGTTAGAAAAACTGTTTTTT	TRBV6-5*01	1		
TGTGCCAGCACCCTTTCGACAGCTTCTAGCACAGATACGCAGTATTTT	TRBV7-9*01	1		
TGTGCCAGCACCTTAACCCCCGACTCCTACGAGCAGTACTTC	TRBV7-2*01	1		
TGTGCCAGCACTAGCGGGGCTAGCTCCTACAATGAGCAGTTCTTC	TRBV6-5*01	1		
TGTGCCAGCACTCCTGGACTAGCGGGGGGCGAGCAGTTCTTC	TRBV12-3*01,TRBV12-4*01	1		
TGTGCCAGCAGACAATTAACAGGGCCCACTGAAGCTTTCTTT	TRBV7-9*01	1		
TGTGCCAGCAGACCTCGGGTGGCCACCGGGGAGCTGTTTTTT	TRBV27*01	1		
TGTGCCAGCAGATCAGGACAGGGCTCGCGGGGCAATCAGCCCCAGCATTTT	TRBV6-2*01,TRBV6-3*01	1		
TGTGCCAGCAGATTAGGGGGGGCATGAGCAGTTCTTC	TRBV7-9*01	1		
TGTGCCAGCAGCACCCTGACAGGGCTGCCTGGCTACACCTTC	TRBV5-5*01	1		
TGTGCCAGCAGCACCGGGACGGACACCTACGAGCAGTACTTC	TRBV7-9*01	1		
TGTGCCAGCAGCAGCCGGGACTCTTCAAACACCGGGGAGCTGTTTTTT	TRBV3-1*01	1		
TGTGCCAGCAGCCAAGATTGGGCCGGTAGGGCCTTTGAGCAGTTCTTC	TRBV14*01	1		
TGTGCCAGCAGCCAAGCAGGGACCTATGGCTACACCTTC	TRBV14*01	1		
TGTGCCAGCAGCCAAGGGAGACCGGGACAGTACTACGAGCAGTACTTC	TRBV3-1*01	1		
TGTGCCAGCAGCCACCGGGGGATATACTCCTACGAGCAGTACTTC	TRBV14*01	1		
TGTGCCAGCAGCCAGGAAGGTGTCTATGGCTACACCTTC	TRBV4-2*01	1		
TGTGCCAGCAGCCAGGGGAATGGGGCCTACGAGCAGTACTTC	TRBV4-2*01	1		
TGTGCCAGCAGCCAGGGGACAGTAAATCAGCCCCAGCATTTT	TRBV6-6*01	1		
TGTGCCAGCAGCCATCCGGGACTAGTTAATGAGCAGTTCTTC	TRBV7-8*01	1		
TGTGCCAGCAGCCCACCACAGGGGAGACTTGAAAAACTGTTTTTT	TRBV7-9*01	1		
TGTGCCAGCAGCCCCCAGATTATTACCGGGGAGCTGTTTTTT	TRBV12-3*01,TRBV12-4*01	1		
TGTGCCAGCAGCCCCGTTAGCGAAAATGAGGAGTTCTTC	TRBV7-2*01	1		

TGTGCCAGCAGCCCCTTCGGCGGGGGGGCATCAAGGTACAATGAGCAGTTCTTC	TRBV11-2*01	1		
TGTGCCAGCAGCCCTTATCAGTACTTC	TRBV6-1*01	1		
TGTGCCAGCAGCCGCCTAGCGGGGTGGAATGAGCAGTTCTTC	TRBV7-9*01	1		
TGTGCCAGCAGCCGTTGGGGACAGCCCTACGAGCAGTACTTC	TRBV7-4*01	1		
TGTGCCAGCAGCCTCAATCCTAGCGGGAGTACTGGCAATGAGCAGTTCTTC	TRBV7-2*01	1		
TGTGCCAGCAGCCTCCCGGGACAGGGTCACAATGAGCAGTTCTTC	TRBV7-9*01	1		
TGTGCCAGCAGCCTCGGACTAGCGGGGGGGAGCTCCTACGAGCAGTACTTC	TRBV7-9*01	1		
TGTGCCAGCAGCCTCTCCCTGGCCGGGGAGCTGTTTTTT	TRBV7-6*01	1		
TGTGCCAGCAGCCTGACAGGGGAAGGCTCCGGGGAGCTGTTTTTT	TRBV5-6*01	1		
TGTGCCAGCAGCGACCGGCTAGCGGGGTACGAGCAGTACTTC	TRBV7-9*01	1		
TGTGCCAGCAGCGCAACGTCCGGGACTAGCGGTTCGGAGACCCAGTACTTC	TRBV7-9*01	1		
TGTGCCAGCAGCGGGGACAGGGGGCTGGAGACCCAGTACTTC	TRBV11-3*01	1		
TGTGCCAGCAGCGTAGATGGGAGGGGGACTAGCCGTAAAGGGCAGTTCTTC	TRBV9*01	1		
TGTGCCAGCAGCGTAGGCGCAGGGGCAACTAATGAAAAACTGTTTTTT	TRBV9*01	1		
TGTGCCAGCAGCGTAGTTGAGGATTGGTGGTATGGCTACACCTTC	TRBV9*01	1		
TGTGCCAGCAGCTCAGAAGCGGGGGGGCGACTTGGGGAGCTGTTTTTT	TRBV7-9*01	1		
TGTGCCAGCAGCTCCCCTACGGAACAAGGAGGAGATACGCAGTATTTT	TRBV12-3*01,TRBV12-4*01	1		
TGTGCCAGCAGCTCCGAAGGACTTCTTGATACGCAGTATTTT	TRBV7-2*01	1		
TGTGCCAGCAGCTCCGGGGGCAATGAGCAGTTCTTC	TRBV11-2*01	1		
TGTGCCAGCAGCTCGCCCGGCCGGTCGGAGACCCAGTACTTC	TRBV27*01	1		
TGTGCCAGCAGCTCTAGCGGGAGTTCTGATACGCAGTATTTT	TRBV7-9*01	1		
TGTGCCAGCAGCTTAAGCCTAGCGGACCCCACCGGGGAGCTGTTTTTT	TRBV7-3*01	1		
TGTGCCAGCAGCTTAGAAATTCTAGCGGGGGGCGAGCAGTACTTC	TRBV7-9*01	1		
TGTGCCAGCAGCTTAGAGCAGGGAGGAGACTATGGCTACACCTTC	TRBV7-9*01	1		
TGTGCCAGCAGCTTAGAGGGAGGGAGAGCCAATGAGCAGTTCTTC	TRBV7-9*01	1		
TGTGCCAGCAGCTTAGCACTAGCGGGAGTCGTAACCGGGGAGCTGTTTTTT	TRBV7-2*01	1		
TGTGCCAGCAGCTTAGCGGGAGCGGGAGGGCGGGCTGAGCAGTTCTTC	TRBV7-8*01	1		
TGTGCCAGCAGCTTAGGAACTACCCCCAATGAGCAGTTCTTC	TRBV7-7*01	1		
TGTGCCAGCAGCTTAGGCGCTTTAAAAGATACGCAGTATTTT	TRBV13*01	1		
TGTGCCAGCAGCTTAGGGAGCTGGCGGGATACGGGAGAGACCCAGTACTTC	TRBV11-2*01	1		
TGTGCCAGCAGCTTAGGGCCAGGGCTTTCCTACGAGCAGTACTTC	TRBV7-2*01	1		
TGTGCCAGCAGCTTAGGGGGATGGGCAGGAGCTTTCTTT	TRBV7-2*01	1		
TGTGCCAGCAGCTTAGTCCCTACGTGGGGCTACACCTTC	TRBV11-3*01	1		
TGTGCCAGCAGCTTAGTGAGCCAAGAGACCCAGTACTTC	TRBV7-9*01	1		
TGTGCCAGCAGCTTATCGACTAGCGGGGCTAGCACAGATACGCAGTATTTT	TRBV7-9*01	1		
TGTGCCAGCAGCTTATTCCTAGCGGTTAGCTCCTACAATGAGCAGTTCTTC	TRBV11-3*01	1		
TGTGCCAGCAGCTTCACCTTCCTGACGGATAACTATGGCTACACCTTC	TRBV7-2*01	1		
TGTGCCAGCAGCTTCAGCGGGAGCTCCTACGAGCAGTACTTC	TRBV5-6*01	1		
TGTGCCAGCAGCTTGAACGGGGAGCTGTTTTTT	TRBV5-4*01	1		
TGTGCCAGCAGCTTGGCGAACTCCTACGAGCAGTACTTC	TRBV7-2*01	1		
TGTGCCAGCAGCTTGGGGGGCAATCAGCCCCAGCATTTT	TRBV7-8*01	1		
TGTGCCAGCAGCTTGGTGGGGGGAGGGAGCGGGAGCACCGGGGAGCTGTTTTTT	TRBV5-5*01	1		
TGTGCCAGCAGCTTGTACAGCCAGACAGATACGCAGTATTTT	TRBV5-4*01	1		
TGTGCCAGCAGCTTGTATCGTTCGGACTTGAACACTGAAGCTTTCTTT	TRBV5-5*01	1		
TGTGCCAGCAGCTTTTCTCCGGACAGGGCGGACACCGGGGAGCTGTTTTTT	TRBV11-2*01	1		
TGTGCCAGCAGGCCCGGGGGCAGCCATACGAAGGGCTACACCTTC	TRBV3-1*01	1		
TGTGCCAGCAGGGGAGGGGGTGAAGAGCAGTACTTC	TRBV6-2*01,TRBV6-3*01	1		
TGTGCCAGCAGGTCCACCGGGACTAGCGTGAGCTCCTACAATGAGCAGTTCTTC	TRBV3-1*01	1		
TGTGCCAGCAGTCCAAGGACAGGGGGTAACGAGCAGTACTTC	TRBV27*01	1		
TGTGCCAGCAGTCCCGGACACTACAATCAGCCCCAGCATTTT	TRBV28*01	1		
TGTGCCAGCAGTCCGCTAGCGGGAGGATCCAAAAACATTCAGTACTTC	TRBV2*01	1		
TGTGCCAGCAGTCCGGACGGCTACGAGCAGTACTTC	TRBV6-5*01	1		
TGTGCCAGCAGTCCGGGCCCCGGTGGGGGCTACACCTTC	TRBV12-3*01,TRBV12-4*01	1		
TGTGCCAGCAGTCTCTCAGGGTGGGGTGAGCAGTTCTTC	TRBV6-1*01	1		
TGTGCCAGCAGTGAAGCCGGCGGTAAGAACACTGAAGCTTTCTTT	TRBV2*01	1		
TGTGCCAGCAGTGAAGGGCTTAGATGGACAGATACGCAGTATTTT	TRBV6-1*01	1		
TGTGCCAGCAGTGAATGGACAGGGGCTAACTATGGCTACACCTTC	TRBV2*01	1		
TGTGCCAGCAGTGACATGACAGGGGGAGATTATGGCTACACCTTC	TRBV2*01	1		
TGTGCCAGCAGTGACCACATGACTGAAGCTTTCTTT	TRBV25-1*01	1		
TGTGCCAGCAGTGAGCTAGCGGACTCCTACGAGCAGTACTTC	TRBV2*01	1		
TGTGCCAGCAGTGATGGCGGGAGGGCCGATGATGAGCAGTTCTTC	TRBV27*01	1		
TGTGCCAGCAGTGCCCGGGACGCCTCCTACGAGCAGTACTTC	TRBV28*01	1		
TGTGCCAGCAGTGCTCAGGGAAACCAGCCCCAGCATTTT	TRBV2*01	1		
TGTGCCAGCAGTGGACAAATTCGGGCGACACCTCAGGGCAATCAGCCCCAGCATTT	TRBV6-1*01	1		
TGTGCCAGCAGTGGCCTAGAGGGGACTGCGGATGGCTACACCTTC	TRBV6-6*01	1		
TGTGCCAGCAGTGTGACAGGGGGAGAGACCCAGTACTTC	TRBV6-5*01	1		
TGTGCCAGCAGTTACTCGACAGCTGCAACTAATGAAAAACTGTTTTTT	TRBV6-5*01	1		
TGTGCCAGCAGTTACTCGGGATCGGGCTCACCCCTCCACTTT	TRBV6-5*01	1		
TGTGCCAGCAGTTACTTTGGCGTCCATGAGCAGTTCTTC	TRBV6-5*01	1		
TGTGCCAGCAGTTCCAGAGATTTGGCCCGCAATCAGCCCCAGCATTTT	TRBV27*01	1		
TGTGCCAGCAGTTGGAACAGGGGCTCCTACGAGCAGTACTTC	TRBV12-3*01,TRBV12-4*01	1		
TGTGCCAGCAGTTGGGACAGGGACTACAATGAGCAGTTCTTC	TRBV27*01	1		
TGTGCCAGCAGTTTAGGCGGCGGGACTGCAGATACGCAGTATTTT	TRBV12-3*01,TRBV12-4*01	1		
TGTGCCAGCAGTTTAGGGCCCACTGAAGCTTTCTTT	TRBV28*01	1		
TGTGCCAGCAGTTTAGGGCGGGGGGGGCCCTACAATGAGCAGTTCTTC	TRBV28*01	1		
TGTGCCAGCAGTTTATCGAGTCTCTACAATGAGCAGTTCTTC	TRBV27*01	1		
TGTGCCAGCAGTTTATCTTGGGGGTATAGCAATCAGCCCCAGCATTTT	TRBV27*01	1		
TGTGCCAGCAGTTTCAGGGTTAGCAATCAGCCCCAGCATTTT	TRBV28*01	1		

TGTGCCAGCAGTTTGGGGCAAAAGAATCAGCCCCAGCATTTT	TRBV28*01	1		
TGTGCCAGCAGTTTTGGGACAGGGGCCTACGAGCAGTACTTC	TRBV28*01	1		
TGTGCCAGCCCCTCAGAGGGGCCCAGGTCCTACAATGAGCAGTTCTTC	TRBV2*01	1		
TGTGCCAGCCCGAACAGACTAGCGGCAGATACGCAGTATTTT	TRBV6-5*01	1		
TGTGCCAGCGAGGAGGTCGGCAGGGGAGGCTATGGCTACACCTTC	TRBV7-9*01	1		
TGTGCCAGCGGCCCGTGGGGGAATCAGCCCCAGCATTTT	TRBV7-2*01	1		
TGTGCCAGCGGCGGGGGGGGCTATGGCTACACCTTC	TRBV6-1*01	1		
TGTGCCAGCTCACCACCGGAGGGAAATGAGCAGTTCTTC	TRBV18*01	1		
TGTGCCAGCTCACCAGAGTTCGGCCAGGGGAACCATGAGCAGTTCTTC	TRBV18*01	1		
TGTGCCAGCTCAGAAGGGACAGCAAATATGAACACTGAAGCTTTCTTT	TRBV18*01	1		
TGTGCCAGTACCCAACCGAGGAGCAATCAGCCCCAGCATTTT	TRBV19*01	1		
TGTGCCAGTACCCCCTGGGCAACTAATGAAAAACTGTTTTTT	TRBV19*01	1		
TGTGCCAGTAGGAGCGACAGCTCTGGGGCCAACGTCCTGACTTTC	TRBV19*01	1		
TGTGCCAGTAGTAATCCGGGACAGTATAGCAATCAGCCCCAGCATTTT	TRBV19*01	1		
TGTGCCAGTAGTACGCCGGGACAGAGAGCCTACGAGCAGTACTTC	TRBV19*01	1		
TGTGCCAGTAGTAGTGAGCAGAACACTGAAGCTTTCTTT	TRBV19*01	1		
TGTGCCAGTAGTATAATGGGGTGGACCGGTGACTCTGGGGCCAACGTCCTGACTT	TRBV19*01	1		
TGTGCCAGTAGTATAGATCCCAGTGAAGCTTTCTTT	TRBV19*01	1		
TGTGCCAGTAGTCAGGGACTAGCGGTTTACGAGCAGTACTTC	TRBV19*01	1		
TGTGCCAGTAGTGCCCGAAATAGCAATCAGCCCCAGCATTTT	TRBV19*01	1		
TGTGCCAGTCTACTAGCGGCCAATGAGCAGTTCTTC	TRBV6-1*01	1		
TGTGCCAGTGGGACAGGGGGCTACGAGCAGTACTTC	TRBV6-2*01,TRBV6-3*01	1		
TGTGCCATCAGGGACAACTATGGCTACACCTTC	TRBV10-3*01	1		
TGTGCCATCAGTGAGACAGGGACCCACGAGCAGTACTTC	TRBV10-3*01	1		
TGTGCCATCAGTGAGACCATCAGGGGCGCCTACAATGAGCAGTTCTTC	TRBV10-3*01	1		
TGTGCCTGGACCCCCGGGACTAGCAACGAGCAGTACTTC	TRBV30*01	1		
TGTGCCTGGAGTGTAGCTAGCGGATTCAGCTCCTACAATGAGCAGTTCTTC	TRBV30*01	1		
TGTGCCTGGAGTGTTTTGGGTGAAGCTTTCTTT	TRBV30*01	1		
TGTGCCAGTAGTATCTTAGGAGGGGCCGGGGAGCTGTTTTTT	TRBV19*01	1		
TGCAGCGTCAAACCCGACCGCATCCTAAACACTGAAGCTTTCTTT	TRBV29-1*01	1		
TGCAGCGTTGAAGGGGTGACTAGCGGGAGGAGCTCCTACAATGAGCAGTTCTTC	TRBV29-1*01	1		
TGCAGTGCTAGAGACCCCGTTGGAGCACTCAATCAGCCCCAGCATTTT	TRBV20-1*01	1		
TGCAGTGCTAGGGGGCAGGGGTTGGGCACTGAAGCTTTCTTT	TRBV20-1*01	1		
TGCGCCAGCAGAGAAGGAACGGGGAACACTGAAGCTTTCTTT	TRBV10-1*01	1		
TGCGCCAGCAGCCGGAACAGGGGGACTGGAAACACCATATATTTT	TRBV5-1*01	1		
TGCGCCAGCAGCTTGGCCGGCGGGAGGGCCAACAATGAGCAGTTCTTC	TRBV5-1*01	1		
TGCGCCAGCAGTGTGACAGACGAGCAGTACTTC	TRBV10-2*01	1		
TGTGCCACCAGAGGACGATTAGCTGGAGCAGATACGCAGTATTTT	TRBV24-1*01	1		
TGTGCCACCAGCAGAGGACACAGCAATCAGCCCCAGCATTTT	TRBV15*01	1		
TGTGCCACCAGTGACGGGATGGTGGTGGCGGAGACCCAGTACTTC	TRBV24-1*01	1		
TGTGCCAGCAAGGGCGACAGTTCGGCCTCGGAGACCCAGTACTTC	TRBV6-6*01	1		
TGTGCCAGCAATTCGGCAGGCACAGATACGCAGTATTTT	TRBV28*01	1		
TGTGCCAGCAGCAAATTCACGTATGAGGGGGTTACCAATCAGCCCCAGCATTTT	TRBV7-4*01	1		
TGTGCCAGCAGCCAAGATCGGGGGTTAGGCACCGGGGAGCTGTTTTTT	TRBV4-2*01	1		
TGTGCCAGCAGCCAAGCGACAGGGAGCTCCTACGAGCAGTACTTC	TRBV4-2*01	1		
TGTGCCAGCAGCCAAGGGACTAGCGGGAGAACCACAGATACGCAGTATTTT	TRBV14*01	1		
TGTGCCAGCAGCCAAGGGGGACATTTGAACATTCAGTACTTC	TRBV3-1*01	1		
TGTGCCAGCAGCCAGACTAGCGGGCCATACTACGAGCAGTACTTC	TRBV3-1*01	1		
TGTGCCAGCAGCCGAGCGGGACTAGCTCTCACCGGGGAGCTGTTTTTT	TRBV7-2*01	1		
TGTGCCAGCAGCCTGAACCATATGAACACTGAAGCTTTCTTT	TRBV7-9*01	1		
TGTGCCAGCAGCGCAGGACTTGGGGCTTTCTTT	TRBV9*01	1		
TGTGCCAGCAGCGGCCAACTATCATACGAGCAGTACTTC	TRBV9*01	1		
TGTGCCAGCAGCTTAGCGTTGCTAGCGGTTCCTTTCAATGAGCAGTTCTTC	TRBV7-8*01	1		
TGTGCCAGCAGCTTCCAGCCTAGCAGATACGAGCAGTACTTC	TRBV13*01	1		
TGTGCCAGCAGGAGGGAGGGGCAGGAGACCCAGTACTTC	TRBV7-9*01	1		
TGTGCCAGCAGGGGGGGACGGTTCCTACGAGCAGTACTTC	TRBV11-3*01	1		
TGTGCCAGCAGTCCTCACCTCTCGGGCAATGAGCAGTTCTTC	TRBV12-3*01,TRBV12-4*01	1		
TGTGCCAGCAGTTCGCGTAGCGTCCAAGAGACCCAGTACTTC	TRBV6-6*01	1		
TGTGCCAGCATCGGCGCAGAATCGGGCGAGCAGTACTTC	TRBV2*01	1		
TGTGCCAGCGATATGACAGGTGGATACACTGAAGCTTTCTTT	TRBV2*01	1		
TGTGCCAGTAGACGGAACATGAACACTGAAGCTTTCTTT	TRBV19*01	1		
TGTGCCAGTAGTATGGGGATCAGGGAGACCCAGTACTTC	TRBV19*01	1		
TGTGCTAGTTTACGGGGAGGTCAGCCCCAGCATTTT	TRBV12-5*01	1		
TGCAGTATGGGACAGGGGGGTTACGAGCAGTACTTC	TRBV20-1*01	1		
TGTGCCAGCAGCTCTAGGCAGAACACCGGGGAGCTGTTTTTT	TRBV11-3*01	1		
TGCAGTGCCCCCCCCCGAGGCACAGATACGCAGTATTTT	TRBV20-1*01	1		
TGCGCCAGCAGCGAAGCGACAGGGAACACCGGGGAGCTGTTTTTT	TRBV5-1*01	1		
TGCGCCAGCAGCTCGGAAGCGGGGGGAATCAATGAGCAGTTCTTC	TRBV5-1*01	1		
TGCGCCAGCAGTGTAGGGCAGGTCGGTCAGCCCCAGCATTTT	TRBV10-2*01	1		
TGTGCCACCGGAACGAGCAATCAGCCCCAGCATTTT	TRBV6-6*01	1		
TGTGCCAGCAGCCAAGATTGGGCTAGCGGGGAGCTGTTTTTT	TRBV3-1*01	1		
TGTGCCAGCAGCGCTCCCACAGATACGCAGTATTTT	TRBV9*01	1		
TGTGCCAGCAGCTTAGCGGGGGGGATGAACACTGAAGCTTTCTTT	TRBV7-2*01	1		
TGTGCCTGGAGTGTGACAGCGAGCTACGAGCAGTACTTC	TRBV30*01	1		
TGTGCCAGCAGACAATACGAGCAGTACTTC	TRBV2*01	1		
TGTGCCAGCAGCTCCGGGGGGGCGGGAGATACGCAGTATTTT	TRBV7-3*01	1		
TGTGCCAGTAGCGCCAGGGACGGCCTCAATCAGCCCCAGCATTTT	TRBV19*01	1		
TGTGCCAGCACCGCTCCTGAGCAGTTCTTC	TRBV11-2*01	1		

TGTGCCAGCAGCCAAGTCCCTGGACAGGGGAATAATTCACCCCTCCACTTT	TRBV4-2*01	1		
TGTGCCAGCAGCAGCTACATTCACAGGCCGGATCCTAGCGGGAGGGTCGACAATG	TRBV13*01	1		
TGTGCCAGCAGCTTGACAGGGATCTCCTACGAGCAGTACTTC	TRBV13*01	1		
TGCAGCGTGCCGGGATTAGCGGACTTC	TRBV29-1*01	1		
TGTGCCAGCAGCCAATCAATAAACAGGGTGCACGAGCAGTACTTC	TRBV7-2*01	1		
TGTGCCAGCAGCCAGGCTGGGGCCAGGGCGCTCCACTTT	TRBV14*01	1		
TGTGCCAGCAGCTTATCAGTAATGAACACTGAAGCTTTCTTT	TRBV7-2*01	1		
TGTGCCACCAGCAGAGGGTCTCAGGGAAAAGAGACCCAGTACTTC	TRBV15*01	1		
TGCGCCAGCAGCCAAGTGAAGGCCCTTTTAAACACTGAAGCTTTCTTT	TRBV4-3*01	1		
TGTGCCAGCAGCCAAGATGGAGGAAACACTGAAGCTTTCTTT	TRBV4-2*01	1		
TGTGCCAGCAGTTCCTCCAGGGGCGGGACCCAGTACTTC	TRBV6-5*01	1		

CD4 KIAA0408 MUT - specific CDR3 Vβ

CDR3 nucleotide sequence	Vβ	TCR reads	Primary	Metastasis
TGTGCCAGCTCACCCCCCCACGGGGCGAACACCGGGGAGCTGTTTTTT	TRBV18*01	4		1
TGTGCCAGCAGTTACCTGTCGCGCGGCACTAATGAAAAACTGTTTTTT	TRBV6-5*01	2		1
TGTGCCAGCAGCCAAGATGCTAGCGGGGTCTACAATGAGCAGTTCTTC	TRBV4-2*01	1		1
TGCGCCAGCAGCTTCACAGGGGACCCGAACACCGGGGAGCTGTTTTTT	TRBV5-1*01	1		1
TGCGCCAGCAGCTTGGACACCCTTGGCTCCTACGAGCAGTACTTC	TRBV5-1*01	1		1
TGTGCCAGCAGCCAAGATGGGGCGGGGGGCCCCACAGATACGCAGTATTTT	TRBV3-1*01	1		1
TGTGCCAGCAGCCAAGGGGATTCACCCCTCCACTTT	TRBV7-6*01	1		1
TGTGCCAGCAGCCCGGACGGACTAGCGGGAGTGGAGACCCAGTACTTC	TRBV11-2*01	1		1
TGTGCCAGCAGCCCTAGTGGGAGAGAAGAGACCCAGTACTTC	TRBV11-3*01	1		1
TGTGCCAGCAGCTTAGGGGCTAGCGGGAGTGAGACCCAGTACTTC	TRBV11-3*01	1		1
TGTGCCAGCAGTAGCAGGACGGGTGCAACTAATGAAAAACTGTTTTTT	TRBV27*01	1		1
TGTGCCAGCAGTGTATCCGGGACAGCGAACACTGAAGCTTTCTTT	TRBV27*01	1		1
TGTGCCCTCAAGATGGGGGCCAGCACTGAAGCTTTCTTT	TRBV10-3*01	1		1
TGCGCCAGCAGCCTAGCAGGGATGAACACTGAAGCTTTCTTT	TRBV5-1*01	1	1	
TGTGCCAGCAGCTTAGACAGGGCAACCCCTGGCTACACCTTC	TRBV7-2*01	37		
TGTGCCAGCAGCACTCGGACAGGATCGGGAAACACCATATATTTT	TRBV7-2*01	34		
TGCGCCAGCAGCCCCCAGCCCGACAGTCGAGAGACCCAGTACTTC	TRBV5-1*01	26		
TGTGCCAGCAGCTCTTTTGGGAATGAGCAGTTCTTC	TRBV5-5*01	18		
TGTGCCAGCAGCCCACCCGGGACAGGGTTTGACACTGAAGCTTTCTTT	TRBV28*01	11		
TGCGCCAGCAGCGCAAACACAGGGGGCTCGAGCACTGAAGCTTTCTTT	TRBV5-1*01	10		
TGTGCCAGCACAATATTACCGGGGGCGGGAGGCTACACCTTC	TRBV2*01	10		
TGTGCCAGCTCCACTAATAATTCACCCCTCCACTTT	TRBV18*01	10		
TGTGCCAGCAGTCAGGGGGCGGTATGGGAGACCCAGTACTTC	TRBV6-6*01	9		
TGCAGTGCTAGAGTACCGGGGATTGCCAAAAACATTCAGTACTTC	TRBV20-1*01	8		
TGTGCCAGCTCACGGCTGACTAGCGGTTCTAGCACAGATACGCAGTATTTT	TRBV18*01	8		
TGTGCCAGCAGCAATCGGGGACTAGCGGGGGAGAAGTTCTTC	TRBV7-2*01	7		
TGTGCCAGCTCACCATCAACGTTTGAAGCTTTCTTT	TRBV18*01	5		
TGCAGTGCTAGGGACAGAGGCCCAGGGACCCAGTACTTC	TRBV20-1*01	4		
TGCGCCAGCAGCCTCCTAGGGACAGGGGATCTCCGTGATGGCTACACCTTC	TRBV5-1*01	4		
TGTGCCAGCAGCTTAGCCCTAGCGGGCTCTAGCACAGATACGCAGTATTTT	TRBV11-2*01	4		
TGCAGTGCGAGACAGGGCGGGAATAGCAATCAGCCCCAGCATTTT	TRBV20-1*01	3		
TGCAGTGCTAGGGGGGGCACCTAATCAGCCCCAGCATTTT	TRBV20-1*01	3		
TGTGCCAGCAAGTTAGGAAAAAGCTACGAGCAGTACTTC	TRBV28*01	3		
TGTGCCAGCAGACCCGGACAGGGGGCATGGAGCACTGAAGCTTTCTTT	TRBV12-3*01,TRBV12-4*01	3		
TGTGCCAGCAGCCCCGGACAGGGGTTGAACACTGAAGCTTTCTTT	TRBV2*01	3		
TGTGCCAGCAGCTCGGGACTAGCGGGGGAGACCCAGTACTTC	TRBV5-6*01	3		
TGTGCCAGCAGTTACTTGTCGGGGCCCTACGAGCAGTACTTC	TRBV6-6*01	3		
TGTGCCAGTAGTATGCCCCCGGGTACCACTGAAGCTTTCTTT	TRBV19*01	3		
TGTGCCAGCTCACCACCGATAAGCGGCACTGAAGCTTTCTTT	TRBV18*01	2		
TGCGCCAGCAGCTTCGGCTCTGCAGGACAAGAGACCCAGTACTTC	TRBV5-1*01	2		
TGCAGCGCAGTCCGTCCTCAAGGCATGAACACTGAAGCTTTCTTT	TRBV29-1*01	2		
TGCAGCGTTGATCTGGGGGGGAGCGGCACTGAAGCTTTCTTT	TRBV29-1*01	2		
TGCAGTGCTAGAGAGACAGGGTCCTATGGCTACACCTTC	TRBV20-1*01	2		
TGCGCCAGCAGAGAAGGGTGGACTGAAGCTTTCTTT	TRBV5-1*01	2		
TGTGCCAGCAGCACTTTAGCGGGGCGCACAGATACGCAGTATTTT	TRBV7-9*01	2		
TGTGCCAGCAGCTTAAGCGGGACCTACGAGCAGTACTTC	TRBV7-2*01	2		
TGTGCCAGCAGCTTAGACAGGGCAACCCCTGGCTACACCTTC	TRBV11-3*01	2		
TGTGCCAGCAGCTTATATTTACAGCCCCAGCATTTT	TRBV7-2*01	2		
TGTGCCAGCAGCTTGGGGGGAATCAGGGGGAGCGTTTACGAGCAGTACTTC	TRBV5-4*01	2		
TGTGCCAGCAGTGGACAGGTCTCCAGTCAGCCCCAGCATTTT	TRBV2*01	2		
TGTGCCAGCCAGGGACAGGCATATGGCTACACCTTC	TRBV25-1*01	2		
TGTGCCAGCTCTGCCCAGGGAGGAAATTCACCCCTCCACTTT	TRBV12-3*01,TRBV12-4*01	2		
TGTGCCAGCAGCTTAGTGGGATCCCTAGCAGATACGCAGTATTTT	TRBV7-8*01	1		
TGTGCCAGCAGTGACCCCGGGGAGGGGGTGACCCAGTACTTC	TRBV6-5*01	1		
TGTGCCAGCAGTTATTCCGGGACTAGCGGGATTGCCGATGAGCAGTTCTTC	TRBV6-6*01	1		
TGCAGTGCTCCAGAACGCGGTGGAAACACCGGGGAGCTGTTTTTT	TRBV20-1*01	1		
TGTGCCAGCAGTTACTTGGGGAGCACTGAAGCTTTCTTT	TRBV6-2*01,TRBV6-3*01	1		
TGCAGTGCCGTTGGCGGGACAGGGGACACTGAAGCTTTCTTT	TRBV20-1*01	1		
TGTGCCAGCACTCCCGGGGCTAGCGGGGTCCTAGGCCAGTACTTC	TRBV6-5*01	1		
TGCGCCAGCAGCTTGGCCCGACTAGCGGGAGCCGGGAGCTCCTACAATGAGCAGTTCTTC	TRBV5-1*01	1		
TGTGCCAGCAGAGTGGGACAGGTAATCTACGAGCAGTACTTC	TRBV6-1*01	1		
TGTGCCAGCAGCGGACGAGCGGGGGGGCCTAAAAATGAGCAGTTCTTC	TRBV7-2*01	1		
TGTGCCAGCAGCTTAGGGACAGGGGGCCAAGGCGGGGAGCTGTTTTTT	TRBV7-6*01	1		
TGTGCCAGTAGTATAGATGAGGGGTCACCCCTCCACTTT	TRBV19*01	1		
TGTGCCAGCACCGGATGGACAGGGGTCCCATTGAATTCACCCCTCCACTTT	TRBV6-2*01,TRBV6-3*01	1		
TGTGCCAGCAGCGCCGCCGGACAGGAATATTTCGGCTACACCTTC	TRBV7-9*01	1		
TGTGCCAGCAGTTCTAGGGTTGGCACTGAAGCTTTCTTT	TRBV28*01	1		
TGCAGCACAACCCGGGAAGCGGGGGGTGAAACAGATACGCAGTATTTT	TRBV29-1*01	1		
TGCAGCGCAGCTAGCGGGAGTGGGGAGACCCAGTACTTC	TRBV29-1*01	1		
TGCAGCGTAAGGAGTCCCTACGAGCAGTACTTC	TRBV29-1*01	1		
TGCAGCGTCCCGGGACATAACACCGGGGAGCTGTTTTTT	TRBV29-1*01	1		
TGCAGCGTCGCCTTAGGATCCACCTACGAGCAGTACTTC	TRBV29-1*01	1		
TGCAGCGTGGCACAGGGGACTCCGAACACCGGGGAGCTGTTTTTT	TRBV29-1*01	1		
TGCAGCGTGGCGGGGGCCTACAATGAGCAGTTCTTC	TRBV29-1*01	1		
TGCAGCGTTACGAATCAGGGAGTCTCCAAAAACATTCAGTACTTC	TRBV29-1*01	1		
TGCAGCGTTGAAGTACAGAGCTACGAGCAGTACTTC	TRBV29-1*01	1		
TGCAGCGTTGAGGGGGGGCGCCCCGGGGAGCTGTTTTTT	TRBV29-1*01	1		
TGCAGCGTTGATAGGGGGTCCCAAGAGACCCAGTACTTC	TRBV29-1*01	1		
TGCAGCGTTGATCCGGGGCACACAGATACGCAGTATTTT	TRBV29-1*01	1		
TGCAGCGTTGGTTCACGGACCACCTACGAGCAGTACTTC	TRBV29-1*01	1		
TGCAGTGCAGGGGACCCCCCCGGGAAGGGTACTTC	TRBV20-1*01	1		
TGCAGTGCCCATCAGGGGACTAGCGGGGGGGGCTTCACAGATACGCAGTATTTT	TRBV20-1*01	1		
TGCAGTGCCCCCGACACCTATGGCTACACCTTC	TRBV20-1*01	1		
TGCAGTGCCTACAAGACTAGCGGTCTTGCTGGGCAGTTCTTC	TRBV20-1*01	1		

TGCAGTGCGGACAGGGATAGCTCCTACAATGAGCAGTTCTTC	TRBV20-1*01	1		
TGCAGTGCTACACAGGGCCAAGAGACCCAGTACTTC	TRBV20-1*01	1		
TGCAGTGCTAGAAACAGCGGGAGGGCCCGAGTCTACGAGCAGTACTTC	TRBV20-1*01	1		
TGCAGTGCTAGAGATGCATCGACCGGGGAGCTGTTTTTT	TRBV20-1*01	1		
TGCAGTGCTAGAGATGGTGGAAACACCATATATTTT	TRBV20-1*01	1		
TGCAGTGCTAGAGCCCTCGACCGAGAGACCCAGTACTTC	TRBV20-1*01	1		
TGCAGTGCTAGAGCCGCCTGGGGACAGAAGGCCTACGAGCAGTACTTC	TRBV20-1*01	1		
TGCAGTGCTAGAGCTTCCGGGGGGAGTCAGCCCCAGCATTTT	TRBV20-1*01	1		
TGCAGTGCTAGAGGGGACAGGGGACGCACAGATACGCAGTATTTT	TRBV20-1*01	1		
TGCAGTGCTAGATATCCGGGACTAGATCACAATGAGCAGTTCTTC	TRBV20-1*01	1		
TGCAGTGCTAGATCCCTCTACACAGATACGCAGTATTTT	TRBV20-1*01	1		
TGCAGTGCTAGATCTCCGACGAACACTGAAGCTTTCTTT	TRBV20-1*01	1		
TGCAGTGCTAGTCCCCGGGGCAGGCCCTACGAGCAGTACTTC	TRBV20-1*01	1		
TGCAGTGCTAGTGGAGGCGCGAAAGGCTACACCTTC	TRBV20-1*01	1		
TGCAGTGCTGGCAGCGGGAGGAGCACAGATACGCAGTATTTT	TRBV20-1*01	1		
TGCAGTGCTGTACAGGGAGGGCCTAATCAGCCCCAGCATTTT	TRBV20-1*01	1		
TGCAGTGTCAGCCTGGGGGCGTATGGCTACACCTTC	TRBV20-1*01	1		
TGCGCCAGCAGAGCGACAGGGGGGACCTACGAGCAGTACTTC	TRBV5-1*01	1		
TGCGCCAGCAGCAGCGGGACTAGCGGTAACACCGGGGAGCTGTTTTTT	TRBV5-1*01	1		
TGCGCCAGCAGCCAACTCGACCGTCTCGCGACAGATACGCAGTATTTT	TRBV4-1*01	1		
TGCGCCAGCAGCCAAGATAGATCAAGCCAGTTCTTC	TRBV4-3*01	1		
TGCGCCAGCAGCCAAGGAGTAGAGACTAGCGGGATGAATACGCAGTATTTT	TRBV4-1*01	1		
TGCGCCAGCAGCCCCACCGCAACGCGGGGGCCGAATGAAAAACTGTTTTTT	TRBV4-1*01	1		
TGCGCCAGCAGCCCCCAAGGGACAGACTTCGGCTACACCTTC	TRBV4-3*01	1		
TGCGCCAGCAGCCCCGGGACAAACAATCAGCCCCAGCATTTT	TRBV5-1*01	1		
TGCGCCAGCAGCCTCCCCGGACAGCGCACTGAAGCTTTCTTT	TRBV5-1*01	1		
TGCGCCAGCAGCGCTGGGACTACCTACGAGCAGTACTTC	TRBV5-1*01	1		
TGCGCCAGCAGCGGGGACAGGGGGACGTACGAGCAGTACTTC	TRBV10-2*01	1		
TGCGCCAGCAGCGTGAACAGGGGTGGCTACACCTTC	TRBV5-1*01	1		
TGCGCCAGCAGCTCCGGGCGCGGGGAGTCCGAGCAGTACTTC	TRBV5-1*01	1		
TGCGCCAGCAGCTCGGGGGGTAACACCATATATTTT	TRBV5-1*01	1		
TGCGCCAGCAGCTCTCAGGGGTCCTACGAGCAGTACTTC	TRBV5-1*01	1		
TGCGCCAGCAGCTGGGGGGACAGGACTGGAAACACCATATATTTT	TRBV5-1*01	1		
TGCGCCAGCAGCTTAATTGGGGGCTCCTACAATGAGCAGTTCTTC	TRBV5-1*01	1		
TGCGCCAGCAGCTTACTACACACGAACTACGAGCAGTACTTC	TRBV5-1*01	1		
TGCGCCAGCAGCTTGGAACCCGCAGGGGATTCACCCCTCCACTTT	TRBV5-1*01	1		
TGCGCCAGCAGCTTGGAATCCGGCAGCAATCAGCCCCAGCATTTT	TRBV5-1*01	1		
TGCGCCAGCAGCTTGGCCCCGGGCACTGAAGCTTTCTTT	TRBV5-1*01	1		
TGCGCCAGCAGCTTGGGACTAGCGGGAGGAATAGATACGCAGTATTTT	TRBV5-1*01	1		
TGCGCCAGCAGCTTGGGACTAGCGGGGGGGGCCTCCTACGAGCAGTACTTC	TRBV5-1*01	1		
TGCGCCAGCAGCTTGGGAGGGCTCCAGGGGTACAATGAGCAGTTCTTC	TRBV5-1*01	1		
TGCGCCAGCAGCTTGGGCGACGGTGGTAATCAGCCCCAGCATTTT	TRBV5-1*01	1		
TGCGCCAGCAGCTTGGTTACAGGGATGAACACTGAAGCTTTCTTT	TRBV5-1*01	1		
TGCGCCAGCAGGCAAAGGGAGCGGGGGGGCCCCTCCTACAATGAGCAGTTCTTC	TRBV10-1*01	1		
TGCGCCAGCAGTCAGAACCCATCGGGACAGGGAGATAACTATGGCTACACCTTC	TRBV5-1*01	1		
TGCGCCAGCCTCTCGTGGACTAGCGGGCGCACAGATACGCAGTATTTT	TRBV10-2*01	1		
TGTGCCACCAGCAGAGATCTGGGCCCAGGGGGACAGGGGGCCGGGGAGCTGTTTTTT	TRBV15*01	1		
TGTGCCACCAGCAGCCGACAGGGGGGTTGGTCCTACAATGAGCAGTTCTTC	TRBV15*01	1		
TGTGCCACCAGGAGACTTAGACAGGCCTACGAGCAGTACTTC	TRBV24-1*01	1		
TGTGCCACCAGTGACACGGGACGCAGCTCCTACAATGAGCAGTTCTTC	TRBV24-1*01	1		
TGTGCCACCAGTGACATAGCGGCAAGCTCCTACAATGAGCAGTTCTTC	TRBV24-1*01	1		
TGTGCCACCAGTGATTTGCGACCGGGGAGCTCCTACAATGAGCAGTTCTTC	TRBV24-1*01	1		
TGTGCCAGCAAAAGCCCCCCTAGCCTTGGGTACTTC	TRBV6-5*01	1		
TGTGCCAGCAAACTAGGGGGCGGAGGGTCCCCCCTCTACGAGCAGTACTTC	TRBV28*01	1		
TGTGCCAGCAACAAACCCCAGGGGGACACTGAAGCTTTCTTT	TRBV27*01	1		
TGTGCCAGCAACTTGGGACGGCCCCAAGAGACCCAGTACTTC	TRBV5-6*01	1		
TGTGCCAGCACAATATTACCGGGGGCGGGAGGCTACACCTTC	TRBV13*01	1		
TGTGCCAGCACAGGGAAAGAGACCCAGTACTTC	TRBV28*01	1		
TGTGCCAGCACCAAACGACAGGGTGAAGCTTTCTTT	TRBV6-5*01	1		
TGTGCCAGCACCGAAAGGGCAGTTCAGCCCCAGCATTTT	TRBV2*01	1		
TGTGCCAGCACCGAGACCCTGGGATGGACTGAAGCTTTCTTT	TRBV2*01	1		
TGTGCCAGCACCGCCAGACTAGGGAATGAGCAGTTCTTC	TRBV6-2*01,TRBV6-3*01	1		
TGTGCCAGCACCGGGGGGGGTGCGTCCTACAATGAGCAGTTCTTC	TRBV6-2*01,TRBV6-3*01	1		
TGTGCCAGCAGAATGACAGGGGTTGTATCCTACGAGCAGTACTTC	TRBV5-6*01	1		
TGTGCCAGCAGACCTATGGGTTGGGCAGGAAACACCATATATTTT	TRBV12-3*01,TRBV12-4*01	1		
TGTGCCAGCAGACTAACAGGGTACCCTACAGATACGCAGTATTTT	TRBV6-1*01	1		
TGTGCCAGCAGACTAGGACTAGCGGGGGCAGATACGCAGTATTTT	TRBV7-9*01	1		
TGTGCCAGCAGACTGGAGGCCTCGAACACTGAAGCTTTCTTT	TRBV2*01	1		
TGTGCCAGCAGCAACTCTCTGAAGTTCTTC	TRBV7-9*01	1		
TGTGCCAGCAGCACTCGGACAGGATCGGGAAACACCATATATTTT	TRBV11-2*01	1		
TGTGCCAGCAGCCAACTCGGGGAGCTGTTTTTT	TRBV3-1*01	1		
TGTGCCAGCAGCCAAGAGGGCCTGTTAGGGACCCAGTACTTC	TRBV3-1*01	1		
TGTGCCAGCAGCCAAGAGTCAGGGGGCGGTCAGCCCCAGCATTTT	TRBV3-1*01	1		
TGTGCCAGCAGCCAAGATCGGCGGGCCCAGGGAGAGACCCAGTACTTC	TRBV14*01	1		
TGTGCCAGCAGCCAAGGGAATAGCGGGGGGATTCAGTACTTC	TRBV3-1*01	1		
TGTGCCAGCAGCCAAGGGCTAGCGGGAGGGCCGCGCGATGAGCAGTTCTTC	TRBV14*01	1		
TGTGCCAGCAGCCAAGTAGGGGGACAGGGGAACACCGGGGAGCTGTTTTTT	TRBV4-2*01	1		
TGTGCCAGCAGCCAGCTTGGGATGAAAAAAGAGACCCAGTACTTC	TRBV14*01	1		
TGTGCCAGCAGCCAGTCCCAGACTACCTCGGGCAATGAGCAGTTCTTC	TRBV14*01	1		
TGTGCCAGCAGCCCAAGCGGGGGGAACACCGGGGAGCTGTTTTTT	TRBV7-2*01	1		
TGTGCCAGCAGCCCATACGTTACATCCACCGAGCAGTACTTC	TRBV27*01	1		
TGTGCCAGCAGCCCGAGCCGGACTAGCGGGCCGAACACCGGGGAGCTGTTTTTT	TRBV9*01	1		
TGTGCCAGCAGCCCTGGGACAGTTAACTATGGCTACACCTTC	TRBV7-8*01	1		
TGTGCCAGCAGCCGAGCGGGAGGGATGTATGAGCAGTTCTTC	TRBV14*01	1		
TGTGCCAGCAGCCGAGTAACTACAACTGAAGCTTTCTTT	TRBV4-2*01	1		
TGTGCCAGCAGCCGCCTCGGGACTAGCGGGGGGCGTCACAATGAGCAGTTCTTC	TRBV28*01	1		
TGTGCCAGCAGCCGGGACAGGGGGGCCGGCTATGGCTACACCTTC	TRBV7-2*01	1		

TGTGCCAGCAGCCGGGACGTCAATCAGCCCCAGCATTTT	TRBV11-3*01	1		
TGTGCCAGCAGCCTAGAGGACCAGACTGTCACAGATACGCAGTATTTT	TRBV6-9*01	1		
TGTGCCAGCAGCCTCGGGACCGCAATGTACCTTAATCAGCCCCAGCATTTT	TRBV7-8*01	1		
TGTGCCAGCAGCCTCTACGGGAGGACCTCTGGAAACACCATATATTTT	TRBV6-6*01	1		
TGTGCCAGCAGCCTGCCCTCGCGGGGGAACACTGAAGCTTTCTTT	TRBV11-2*01	1		
TGTGCCAGCAGCCTGGACAGGTCTACTGAAGCTTTCTTT	TRBV2*01	1		
TGTGCCAGCAGCGACCGGGGCTACACCGGGGAGCTGTTTTTT	TRBV7-2*01	1		
TGTGCCAGCAGCGCGGGTAGCGGGATGGGGGCCTATGAGCAGTTCTTC	TRBV3-1*01	1		
TGTGCCAGCAGCGGATGGGTCGTGGGTGAGCAGTTCTTC	TRBV7-2*01	1		
TGTGCCAGCAGCGGCCAGGGGGTTGGTGAGCAGTTCTTC	TRBV9*01	1		
TGTGCCAGCAGCGTACAGGGGATTGGGGGGGTGCAGTACTTC	TRBV7-2*01	1		
TGTGCCAGCAGCGTAGAGGTATGGGAGACCCAGTACTTC	TRBV9*01	1		
TGTGCCAGCAGCGTAGCGACAGGGGGCAGGAGTCAGCCCCAGCATTTT	TRBV9*01	1		
TGTGCCAGCAGCGTAGGGGTAGCGGGAGTCTCTGAGCAGTTCTTC	TRBV9*01	1		
TGTGCCAGCAGCTCAGAACCCGGGACAGGGGGCGGTGAAAAACTGTTTTTT	TRBV7-2*01	1		
TGTGCCAGCAGCTCCACAGGGGGTGGGGAAGCTTTCTTT	TRBV11-2*01	1		
TGTGCCAGCAGCTCCCTCGGGGGACAGGGGTCCAAAAACATTCAGTACTTC	TRBV9*01	1		
TGTGCCAGCAGCTCCGCGGCTCTAGCGGGGGGTGCCGGGGAGCTGTTTTTT	TRBV7-3*01	1		
TGTGCCAGCAGCTCGAGGGGGCGGGGCCAGTACTTC	TRBV7-2*01	1		
TGTGCCAGCAGCTCGCCCACAGGGAAGAGCGGGGAGCTGTTTTTT	TRBV7-9*01	1		
TGTGCCAGCAGCTCGCCGGGACTAGCGGGAGGGCCTCAAGGGGCAGATACGCAGTATTTT	TRBV5-6*01	1		
TGTGCCAGCAGCTCGGACAGGTTCGACAATGAGCAGTTCTTC	TRBV7-9*01	1		
TGTGCCAGCAGCTCGGGACTAGCGGGAATTCAGTACTTC	TRBV7-7*01	1		
TGTGCCAGCAGCTTAACGTATCTCAGGAACACTGAAGCTTTCTTT	TRBV7-3*01	1		
TGTGCCAGCAGCTTAAGGCTTGGCGGGGGGGCGGTTGGTACGCAGTATTTT	TRBV7-2*01	1		
TGTGCCAGCAGCTTACGGGCGGGAGGGTTCGACGAGCAGTACTTC	TRBV7-6*01	1		
TGTGCCAGCAGCTTACGTACAGGGAGGGCCTCTGGAAACACCATATATTTT	TRBV5-4*01	1		
TGTGCCAGCAGCTTACTAGCGGGCTACGAGCAGTACTTC	TRBV7-3*01	1		
TGTGCCAGCAGCTTAGAATCGGCCCAGTACTTC	TRBV11-3*01	1		
TGTGCCAGCAGCTTAGACCGTTACACAGATACGCAGTATTTT	TRBV7-2*01	1		
TGTGCCAGCAGCTTAGATAATAATCAGCCCCAGCATTTT	TRBV7-2*01	1		
TGTGCCAGCAGCTTAGCGCGTCATAGCGGGACAGGGACCTACAATGAGCAGTTCTTC	TRBV7-2*01	1		
TGTGCCAGCAGCTTAGCGGGGGCGGATACGCAGTATTTT	TRBV11-2*01	1		
TGTGCCAGCAGCTTAGGAAACCGACAGTCCTACGAGCAGTACTTC	TRBV11-2*01	1		
TGTGCCAGCAGCTTAGGACAGGGTCGTATAAATGAGCAGTTCTTC	TRBV7-2*01	1		
TGTGCCAGCAGCTTAGGACTGAAAGGCTACACCTTC	TRBV7-2*01	1		
TGTGCCAGCAGCTTAGGAGGAGCGGGACCGTACGAGCAGTACTTC	TRBV7-9*01	1		
TGTGCCAGCAGCTTAGGGACAACCCAAGAGACCCAGTACTTC	TRBV5-5*01	1		
TGTGCCAGCAGCTTAGGGCAGGGGATTTACTATGGCTACACCTTC	TRBV11-2*01	1		
TGTGCCAGCAGCTTAGGGCAGGGGCCTTACGAGCAGTACTTC	TRBV7-2*01	1		
TGTGCCAGCAGCTTAGGTCAGGGGATGGACACTGAAGCTTTCTTT	TRBV7-2*01	1		
TGTGCCAGCAGCTTATCAGGGGACAGTAGCAATCAGCCCCAGCATTTT	TRBV7-2*01	1		
TGTGCCAGCAGCTTATGGGTCCCACAGGGTGGGAATTCACCCCTCCACTTT	TRBV7-2*01	1		
TGTGCCAGCAGCTTCGGGGGACAGCAGCGGGGCTATGGCTACACCTTC	TRBV11-3*01	1		
TGTGCCAGCAGCTTGCCCATCGAAGAGACCCAGTACTTC	TRBV5-4*01	1		
TGTGCCAGCAGCTTGGGAGTGCGGTGGTCTAACTATGGCTACACCTTC	TRBV5-6*01	1		
TGTGCCAGCAGCTTGTTCCGTCGACTAGCGGGCACAGATACGCAGTATTTT	TRBV5-6*01	1		
TGTGCCAGCAGGAGGACTAGCGGGGGAGGCCAAGAGACCCAGTACTTC	TRBV7-2*01	1		
TGTGCCAGCAGGGCCAGCGGGGGCATTCAAGAGACCCAGTACTTC	TRBV6-2*01,TRBV6-3*01	1		
TGTGCCAGCAGGGGACGGGGGACTAGCGGGTTGCAGTTCTTC	TRBV25-1*01	1		
TGTGCCAGCAGGGGGGTTCGGGCGGGGTGAAGCTTTCTTT	TRBV28*01	1		
TGTGCCAGCAGGTGGACAGCAGGAGAAGAGACCCAGTACTTC	TRBV5-6*01	1		
TGTGCCAGCAGTAAACAGACAGGAGCTAACTATGGCTACACCTTC	TRBV6-5*01	1		
TGTGCCAGCAGTATACGAGGACAGGGAAACACCGGGGAGCTGTTTTTT	TRBV11-2*01	1		
TGTGCCAGCAGTCCCGGACAGGGTCGGGCCGGGGAGCTGTTTTTT	TRBV6-2*01,TRBV6-3*01	1		
TGTGCCAGCAGTCCGGGGACAGGGCTGAGAAACACAGATACGCAGTATTTT	TRBV25-1*01	1		
TGTGCCAGCAGTCTCCGACACCAGGGCGCCGGGGAGCTGTTTTTT	TRBV12-3*01,TRBV12-4*01	1		
TGTGCCAGCAGTGAAGCAAGCCTGACCCTCGCCCCCGCGAGTTACAATGAGCAGTTCTTC	TRBV6-1*01	1		
TGTGCCAGCAGTGAGTATCGCGAGCAGTACTTC	TRBV2*01	1		
TGTGCCAGCAGTGCACAGACGAACACTGAAGCTTTCTTT	TRBV28*01	1		
TGTGCCAGCAGTGGCCGGGACGGGAACACCGGGGAGCTGTTTTTT	TRBV6-1*01	1		
TGTGCCAGCAGTGTAAGGACAGGGGGCAGTGAGCAGTTCTTC	TRBV25-1*01	1		
TGTGCCAGCAGTTACGGGGATATCGCCTACAATGAGCAGTTCTTC	TRBV6-6*01	1		
TGTGCCAGCAGTTACTCGAATACCTACAATGAGCAGTTCTTC	TRBV6-6*01	1		
TGTGCCAGCAGTTACTTCGGGGGGAAGTCCAATGAGCAGTTCTTC	TRBV6-2*01,TRBV6-3*01	1		
TGTGCCAGCAGTTATCTGAGAGGGGGCGCGTACGAGCAGTACTTC	TRBV6-5*01	1		
TGTGCCAGCAGTTATTCCCGGACAGCAAATCAGCCCCAGCATTTT	TRBV6-5*01	1		
TGTGCCAGCAGTTCCAGGACAGGGCTTTCCTACAATGAGCAGTTCTTC	TRBV12-3*01,TRBV12-4*01	1		
TGTGCCAGCAGTTCCGAGCGGACAGGGCGGAATTCACCCCTCCACTTT	TRBV6-5*01	1		
TGTGCCAGCAGTTTAACCACAATTAGTGAAGCTTTCTTT	TRBV12-3*01,TRBV12-4*01	1		
TGTGCCAGCAGTTTAAGCCGGGAGCAGTACTTC	TRBV12-3*01,TRBV12-4*01	1		
TGTGCCAGCAGTTTAGGCCAGCCCCAGCATTTT	TRBV12-3*01,TRBV12-4*01	1		
TGTGCCAGCAGTTTGGTCCTCAATGAGCAGTTCTTC	TRBV28*01	1		
TGTGCCAGCAGTTTTCTGGGGAGCTCTGGAAACACCATATATTTT	TRBV12-3*01,TRBV12-4*01	1		
TGTGCCAGCATCTCAAGGACAGGAGGGGGCTACACCTTC	TRBV28*01	1		
TGTGCCAGCATTTCCGACAGGAGCTACGAGCAGTACTTC	TRBV6-5*01	1		
TGTGCCAGCCACTCGGGACAACACTCTGGAAACACCATATATTTT	TRBV3-1*01	1		
TGTGCCAGCGGGGCCGGTCCGAAGGAGACCCAGTACTTC	TRBV2*01	1		
TGTGCCAGCTCACCACGGCCCGGGGACGGCTACACCTTC	TRBV18*01	1		
TGTGCCAGCTCACCACGGGGACAAGCGCTGGGAGAGCAGTTCTTC	TRBV18*01	1		
TGTGCCAGCTCACCAGCTAGGGACGGCTACACCTTC	TRBV18*01	1		
TGTGCCAGCTCACCAGGGGCGGCAGATACGCAGTATTTT	TRBV18*01	1		
TGTGCCAGCTCACCAGGGGGACCGCTGTGGGATGGCTACACCTTC	TRBV18*01	1		
TGTGCCAGCTCACCCGGGACAGGGGGCACTTGGGAGACCCAGTACTTC	TRBV18*01	1		
TGTGCCAGCTCACGAGATAAGGAGACTAGCATTAGGGAGACCCAGTACTTC	TRBV7-9*01	1		
TGTGCCAGCTCGCCCAACACCGGGACATCCAACACTGAAGCTTTCTTT	TRBV18*01	1		
TGTGCCAGTAGTACACAGGGTATTAATTCACCCCTCCACTTT	TRBV19*01	1		

TGTGCCAGTAGTATAGATCTATCGTCACAGGAGGATTTT	TRBV19*01	1		
TGTGCCAGTAGTATCCGACTCCGGGACATAAGCAATCAGCCCCAGCATTTT	TRBV19*01	1		
TGTGCCAGTAGTATCGGAGTGGGGGATGGCCTAAATCAGCCCCAGCATTTT	TRBV19*01	1		
TGTGCCAGTGCCAGGACAGGGGGCAACGAGCAGTACTTC	TRBV12-3*01,TRBV12-4*01	1		
TGTGCCAGTTCCGCCGAGCGGGATCGCACAGATACGCAGTATTTT	TRBV19*01	1		
TGTGCCATCAGAGGAGATACCACAGATACGCAGTATTTT	TRBV10-3*01	1		
TGTGCCATCAGCGCTAGCGGGACGAACACAGATACGCAGTATTTT	TRBV10-3*01	1		
TGTGCCATCAGTGAGCAGGGCGGGAGCTATGGCTACACCTTC	TRBV10-3*01	1		
TGTGCCATCAGTGAGTCTGTGGGAGGGCCCTCCCATGAGCAGTTCTTC	TRBV10-3*01	1		
TGTGCCATCAGTGGTCGTGGGACCCGGTCCTACGAGCAGTACTTC	TRBV10-3*01	1		
TGTGCCATTAGCGGCCCCACAGATACGCAGTATTTT	TRBV12-3*01,TRBV12-4*01	1		
TGTGCCGCCGGGGGGCGGATCTATGGCTACACCTTC	TRBV6-5*01	1		
TGTGCCTGGACCACACGGTCTAGCGGCCGGGAAACAGATACGCAGTATTTT	TRBV30*01	1		
