# Supplementary material for: Targeting the tumor mutanome for personalized vaccination in a TMB low non-small cell lung cancer
Source: J Immunother Cancer. 2022 Mar 30;10(3):e003821. doi: 10.1136/jitc-2021-003821 (PMC8971766; doi:10.1136/jitc-2021-003821)
Supplement: Supplementary data [file jitc-2021-003821supp004.pdf]

CD8 neoantigen-specific CDR3 Vβ shared with tumour tissue

Neoantigen specificity	CDR3 nucleotide sequence	Vβ	TCR reads	Primary	Metastasis
MAFF MUT	TGCAGCGTTGAAGGTGTATCTAGCGGGAGCTCCTACAATGAGCAGTTCTTC	TRBV29-1*01	1	1	
MAFF MUT	TGTGCCAGTAGTATGGGGACCGGGTTCGGCGCTTTCTTT	TRBV19*01	1	1	
MAFF MUT	TGTGCCAGCAGCATTCTCCCAGACAATGAGCAGTTCTTC	TRBV11-3*01	1		1
MAFF WT	TGTGCCAGCTCACCACCGGAGGGGGCTGAAGCTTTCTTT	TRBV18*01	1	1	
MAFF WT	TGTGCCAGCAGCCGAGGCACAGGGGGTGAGGAGGATACGCAGTATTTT	TRBV5-6*01	1		1
MAFF WT	TGTGCCAGCAGCTTTTCAGGGTCGAACACTGAAGCTTTCTTT	TRBV5-6*01	1		1
POC1B MUT	TGTGCCAGCAGTCGCCCGCCCGATACGCAGTATTTT	TRBV27*01	1	1	
POC1B MUT	TGTGCCAGCAGTTTCGGAGGGGGAGAGCAGTACTTC	TRBV28*01	1		1
NIF3L1 MUT	TGTGCCAGCAGCTCGACAGGGGGCAGGTTGGATGAGCAGTTCTTC	TRBV7-2*01	1		1
NIF3L1 MUT	TGTGCCAGCAGCCCCGGGACAGGGAACGAGCAGTACTTC	TRBV7-6*01	1		2
NIF3L1 MUT	TGTGCCCACAGGGGGGCAGGGGCCCGTGAGGGCAATCAGCCCCAGCATTTT	TRBV19*01	3		1
NIF3L1 WT	TGCGCCAGCAGCCAAGACGGGCAGGGGGACACTGAAGCTTTCTTT	TRBV4-1*01	1	1	
NIF3L1 WT	TGTGCCAGCAGTTATAGCAGGGAAAAAGAGACCCAGTACTTC	TRBV6-6*01	1		1
NIF3L1 WT	TGTGCCAGCAGCACGGACAGGGCCCTCTACGAGCAGTACTTC	TRBV7-2*01	1		2
KEAP1 MUT	TGTGCCAGCAGTTTATCTTTCGGACTCGAGGGTTACTTC	TRBV27*01	1	1	
KEAP1 MUT	TGCAGTGCTAGAATCATTCACTGGGACGGGGGGGACCCCCAAGAGACCCAGTACTTC	TRBV20-1*01	1		1
KEAP1 MUT	TGTGCCAGCAGCTCAGACCGGGACAGACCGGACACCGGGGAGCTGTTTTTT	TRBV7-8*01	1		1
KEAP1 MUT	TGTGCCAGCAGTACCACAGGCTCCTACGAGCAGTACTTC	TRBV28*01	1		1
KEAP1 MUT	TGTGCCAGCAGTTTGTCACGGGGCTATGGCTACACCTTC	TRBV27*01	1		1
KEAP1 MUT	TGTGCCAGCAGCCCGGACTCTCCTGGGTCCAATGAGCAGTTCTTC	TRBV28*01	1		2
KEAP1 WT	TGTGCCAGCAGCCCAGGGACGAGTAGCACAGATACGCAGTATTTT	TRBV7-9*01	1		1
KEAP1 WT	TGCGCCAGCAGCTCCTTGGACAGGGTTGTTGGCGGGGAGCTGTTTTTT	TRBV5-1*01	3	1	
KEAP1 WT	TGTGCCAGCAGCCAAGTATCAAGGGAACCCGTTTTTGAGACCCAGTACTTC	TRBV4-2*01	14	1	
KIAA0408 MUT	TGTGCCAGCAGCCCAGGGGTGGGCTACACCTTC	TRBV27*01	1	1	
KIAA0408 MUT	TGTGCCAGCAGTCCGTGGGCGGGAGCAGATACGCAGTATTTT	TRBV12-3*01,TRBV12-4*01	1	1	
KIAA0408 MUT	TGCGCCAGCAGCCAAGATCGAAGGGGGGGAAACTACGAGCAGTACTTC	TRBV4-3*01	1	1	
KIAA0408 MUT	TGCAGTGCTAGCAGACTAGCAGGGGACAATGAGCAGTTCTTC	TRBV20-1*01	1		1
KIAA0408 MUT	TGTGCCAGCAGTTACCAGGGGCCCACTTATGGCTACACCTTC	TRBV6-6*01	1		1
KIAA0408 MUT	TGTGCCAGCAGCGTCGGGACAGGGGTCCACGAGCAGTACTTC	TRBV9*01	1		1
KIAA0408 MUT	TGCGCCAGCAGCGGTGCGGGGGGGTTGGAGACCCAGTACTTC	TRBV5-1*01	1	7	2
KIAA0408 MUT	TGTGCCAGTAGTATAGGTGGCAGCTCCTACAATGAGCAGTTCTTC	TRBV19*01	15		1

CD4 neoantigen-specific CDR3 Vβ shared with tumour tissue					
Neoantigen specificity	CDR3 nucleotide sequence	Vβ	TCR reads	Primary	Metastasis
KIAA0408 MUT	TGCGCCAGCAGCCTAGCAGGGATGAACACTGAAGCTTTCTTT	TRBV5-1*01	1	1	NA
KIAA0408 MUT	TGTGCCAGCAGCCAAGATGCTAGCGGGGTCTACAATGAGCAGTTCTTC	TRBV4-2*01	1		1
KIAA0408 MUT	TGCGCCAGCAGCTTCACAGGGGACCCGAACACCGGGGAGCTGTTTTTT	TRBV5-1*01	1		1
KIAA0408 MUT	TGCGCCAGCAGCTTGGACACCCTTGGCTCCTACGAGCAGTACTTC	TRBV5-1*01	1		1
KIAA0408 MUT	TGTGCCAGCAGCCAAGATGGGGCGGGGGGCCCCACAGATACGCAGTATTTT	TRBV3-1*01	1		1
KIAA0408 MUT	TGTGCCAGCAGCCAAGGGGATTCACCCCTCCACTTT	TRBV7-6*01	1		1
KIAA0408 MUT	TGTGCCAGCAGCCCGGACGGACTAGCGGGAGTGGAGACCCAGTACTTC	TRBV11-2*01	1		1
KIAA0408 MUT	TGTGCCAGCAGCCCTAGTGGGAGAGAAGAGACCCAGTACTTC	TRBV11-3*01	1		1
KIAA0408 MUT	TGTGCCAGCAGCTTAGGGGCTAGCGGGAGTGAGACCCAGTACTTC	TRBV11-3*01	1		1
KIAA0408 MUT	TGTGCCAGCAGTAGCAGGACGGGTGCAACTAATGAAAAACTGTTTTTT	TRBV27*01	1		1
KIAA0408 MUT	TGTGCCAGCAGTGTATCCGGGACAGCGAACACTGAAGCTTTCTTT	TRBV27*01	1		1
KIAA0408 MUT	TGTGCCCTCAAGATGGGGGCCAGCACTGAAGCTTTCTTT	TRBV10-3*01	1		1
KIAA0408 MUT	TGTGCCAGCAGTTACCTGTCGCGCGGCACTAATGAAAAACTGTTTTTT	TRBV6-5*01	2		1
KIAA0408 MUT	TGTGCCAGCTCACCCCCCCACGGGGCGAACACCGGGGAGCTGTTTTTT	TRBV18*01	4		1
KEAP1 MUT	TGCGCCAGCCGAGAACTAGCAGGCACAGATACGCAGTATTTT	TRBV5-1*01	1	1	4
KEAP1 MUT	TGTGCCAGCAGCTTGGGCCCCGGGACACATTTGCAGTTCTTC	TRBV5-6*01	1		1
NIF3L1 WT	TGTGCCAGCAGCTTAGAGGGATTAAGCAATCAGCCCCAGCATTTT	TRBV7-2*01	1		2
NIF3L1 WT	TGCAGTGCTAGTGATTCCGGGGAGCTGTTTTTT	TRBV20-1*01	1	1	
NIF3L1 WT	TGTGCCAGCAGTTTATCTGGGACTTCTGGAAACACCATATATTTT	TRBV12-3*01,TRBV12-4*01	1		1
POC1B MUT	TGTGCCAGCAGTCCGTGGGCGGGAGCAGATACGCAGTATTTT	TRBV12-3*01,TRBV12-4*01	1	1	
POC1B MUT	TGTGCCAGCTCGGGGGACAGTAGCTACTACGAGCAGTACTTC	TRBV18*01	1	2	1
POC1B MUT	TGTGCCAGTAGTATACCAACCGGGGGGACAAATGGCTACACCTTC	TRBV19*01	1		1
POC1B MUT	TGTGCCAGCAGCCAAGAAGCAACCGGGACCATGAACACTGAAGCTTTCTTT	TRBV14*01	1		2
POC1B MUT	TGTGCCAGCAGTCGCCCGCCCGATACGCAGTATTTT	TRBV27*01	1	1	
POC1B MUT	TGTGCCAGCAGTTTCGGAGGGGGAGAGCAGTACTTC	TRBV28*01	1		1
POC1B MUT	TGTGCCAGCTCACCACCGGAGGGGGGCTGAAGCTTTCTTT	TRBV18*01	1	1	
POC1B MUT	TGTGCCATCAGTGAAAGGGGGGAGGCCTACGAGCAGTACTTC	TRBV10-3*01	2		1
POC1B MUT	TGTGCCAGCAGTGAATCGATGGACAGTAACACTGAAGCTTTCTTT	TRBV25-1*01	2	1	4
POC1B MUT	TGTGCCAGCAGTTTAATGGGGAGCACCGGGGAGCTGTTTTTT	TRBV28*01	2		6
POC1B MUT	TGTGCCAGCAGTCCCCTGCCGGGACCCATGAACACTGAAGCTTTCTTT	TRBV27*01	22		6
MAFF WT	TGTGCCAGCAGCCCACAGGGGTTGGGAGAGACCCAGTACTTC	TRBV5-6*01	1		1
MAFF WT	TGTGCCAGCAGTTGGAGGAGCTACTCTGGAAACACCATATATTTT	TRBV12-3*01,TRBV12-4*01	1		2
MAFF WT	TGCAGTGCCCAACGGGGCCCCCCGGGGCAGTTCTTC	TRBV20-1*01	1	1	
MAFF WT	TGTGCCAGCAGTTGGCCCCCTGAGACCACTGAAGCTTTCTTT	TRBV6-2*01,TRBV6-3*01	1		1
MAFF MUT	TGTGCCAGCAGTTATGATGCGGGAGTACGAAATACGCAGTATTTT	TRBV6-2*01,TRBV6-3*01	1		1
MAFF MUT	TGCGCCAGCAGCCAAGAGTTGCAGCTTAACACTGAAGCTTTCTTT	TRBV4-1*01	1	3	
MAFF MUT	TGCAGTGCTAGAGATGCGGGTTCTAGCACAGATACGCAGTATTTT	TRBV20-1*01	1		1
MAFF MUT	TGCGCCAGCAGCCAAGATCGAAGGGGGGGAAACTACGAGCAGTACTTC	TRBV4-3*01	1	1	
MAFF MUT	TGTGCCAGCAGCTTAGCAGGGGGTCAGCCCCAGCATTTT	TRBV7-2*01	1		2
